# The GJB3 correlates with the prognosis, immune cell infiltration, and therapeutic responses in lung adenocarcinoma

**DOI:** 10.1515/med-2024-0974

**Published:** 2024-08-10

**Authors:** Ruigang Dou, Rongfeng Liu, Peng Su, Xiaohui Yu, Yanzhao Xu

**Affiliations:** Department of Thoracic Surgery, The First Affiliated Hospital of Xingtai Medical College, Xingtai 054000, Hebei, P. R. China; Department of Oncology, Fourth Hospital of Hebei Medical University, Shijiazhuang 050011, Hebei, P. R. China; Department of Thoracic Surgery, Fourth Hospital of Hebei Medical University, Shijiazhuang 050011, Hebei, P. R. China; Department of Computer Science and Technology, Tangshan Normal University, Tangshan 050011, Hebei, P. R. China; Department of Thoracic Surgery, Fourth Hospital of Hebei Medical University, No. 12 Jiankang Road, Shijiazhuang 050011, Hebei, P. R. China

**Keywords:** lung adenocarcinoma, *GJB3*, transcription factors, hsa-miR-6511b-5p, immune, drug sensitivity

## Abstract

Gap junction protein beta 3 (*GJB3*) has been reported as a tumor suppressor in most tumors. However, its role in lung adenocarcinoma (LUAD) remains unknown. The purpose of this study is to explore the role of *GJB3* in the prognosis and tumor microenvironment of LUAD patients. The data used in this study were acquired from The Cancer Genome Atlas, Gene Expression Omnibus, and imvigor210 cohorts. We found that *GJB3* expression was increased in LUAD patients and correlated with LUAD stages. LUAD patients with high *GJB3* expression exhibited a worse prognosis. A total of 164 pathways were significantly activated in the *GJB3* ^high^ group. *GJB3* expression was positively associated with nine transcription factors and might be negatively regulated by hsa-miR-6511b-5p. Finally, we found that immune cell infiltration and immune checkpoint expression were different between the *GJB3* ^high^ and *GJB3* ^low^ groups. In summary. *GJB3* demonstrated high expression levels in LUAD patients, and those with elevated *GJB3* expression displayed unfavorable prognoses. Additionally, there was a correlation between *GJB3* and immune cell infiltration, as well as immune checkpoint expression in LUAD patients

## 1 Introduction

Lung cancer is a life-threatening and aggressive type of cancer worldwide, and it is a primary cause of cancer-related mortality [1]. It is estimated that lung cancer occurred for approximately 11.4% of all new cancer cases and is responsible for 1.8 million deaths (18% of all cancer-associated mortalities) in 2020 [2]. Previous studies have reported that smoking is closely associated with the incidence of lung cancer [3,4]. Higher smoking intensity and duration could increase the risk and mortality of lung cancer, while a more extended time since smoking cessation can reverse this phenomenon [5], and former smokers who have recently quit smoking have a 50–80% reduction of lung cancer risk [6]. In addition, age, genetic factors, ethnicity, and race are also correlated with the incidence of lung cancer [7]. In recent years, the therapy and diagnosis of lung cancer have required some improvements, but the prognosis and outcome of lung cancer patients remain suboptimal [8].

According to histopathology, lung cancer is generally divided into two cell types, small cell lung cancer and non-small cell lung cancer (NSCLC). The NSCLC includes three histologic subtypes, squamous cell carcinoma, lung adenocarcinoma (LUAD), and large cell carcinoma, roughly accounting for 80–85% of lung cancer [9], and LUAD is the most common subtype of NSCLC [10]. LUAD is prone to metastasize at an early stage, and the average 5-year survival rate is less than 20% [11]. The therapeutic approaches for LUAD mainly include surgical resection, chemotherapy, radiotherapy, and immunotherapy [12]. Identifying patients with LUAD in the early stages is challenging due to metastasis occurring at the time of diagnosis [13]. Moreover, LUAD cells are able to rapidly acquire drug resistance after initial treatment and usually cannot be treated with chemotherapeutic agents [14,15]. Therefore, the 5-year overall survival rate of LUAD remains poor [8].

Gap junction protein beta 3 (*GJB3*) is a member of the connexin gene family, and it encodes the gap junction protein connexin 31(Cx31). It is reported that the mutation of *GJB3* could cause erythrokeratoderma variabilis [16] and autosomal recessive/dominant non-syndromic hearing loss [17,18]. In addition, *GJB3* has been reported as a tumor suppressor in the majority of tumors. In metastatic breast cancer of the bone, the patients with high *GJB3* expression exhibit better prognosis [19], and the high *GJB3* expression could reduce the migration, proliferation, and invasion ability of thyroid cancer cells [20]. However, the *GJB3* is up-regulated in pancreatic ductal adenocarcinoma (PDAC) liver metastasis and *GJB3* depletion could suppress the hepatic metastasis of PDAC cells [21]. However, the mechanism and function of *GJB3* in tumorigenesis and the development of LUAD remain largely unknown. Thus, we systematically explored the potential effects of *GJB3* in tumor microenvironment (TME), immunotherapy, and drug sensitivity of LUAD patients utilizing the bioinformatics methods.

## 2 Materials and methods

### 2.1 Subjects

The mRNA expression profiles of 585 samples with corresponding clinical information, including 526 LUAD and 59 normal samples, were acquired from The Cancer Genome Atlas (TCGA, https://tcga-data.nci.nih.gov/tcga/) database. Among 585 samples, 502 samples contained complete survival information. The miRNA expression profile, copy number variation, and Mutation Annotation Format files were downloaded for subsequent analyses. Cell line expression profiles were downloaded from Cancer Cell Line Encyclopedia (CCLE, https://sites.broadinstitute.org/ccle).

The GSE36471 and GSE43458 datasets were collected from Gene Expression Omnibus (GEO, https://www.ncbi.nlm.nih.gov/geo/). GSE36471 included 117 LUAD samples (114 with complete survival information), and GSE43458 contained 80 LUAD and 30 normal samples.

Moreover, the 348 immunotherapy-experienced BLCA samples containing immunotherapy response status and survival information were obtained from the imvigor210 cohort.

### 2.2 Differential gene analysis

The differential gene analysis was performed between two groups utilizing the “limma” [22] function package in the R language (version 4.2.1, the same as below). The differentially expressed genes (DEGs) were screened by |Log_2_FC| > 1 and FDR < 0.05. The DEGs were then analyzed for Gene ontology (GO, comprising biological process [BP], molecular function [MF], and cellular component [CC]) and Kyoto Encyclopedia of Genes and Genomes (KEGG) enrichment using the “clusterProfiler” function package (version 4.7.1.2) [23] in R language. Significantly enriched pathways were discovered at *P* < 0.05.

### 2.3 Gene set enrichment analysis (GSEA)

In the TCGA cohort, the patients were split into *GJB3* ^high^ and *GJB3* ^low^ groups according to the median *GJB3* expression. The GSEA was performed using the R language function package “clusterprofiler” [23]. The significantly enriched pathways were screened by *P* < 0.05.

### 2.4 Survival analysis

The overall survival of different groups was estimated using the R language “survival” (https://cran.r-project.org/package=survival) and “survminer” packages (https://cran.r-project.org/package=survminer). The significance of differences in survival rates between different groups was tested using the log-rank. The multivariate Cox regression model was used to analyze whether the target gene could predict the survival of LUAD patients independently of other factors.

### 2.5 Nomogram prognostic model establishment

R language “rms” (https://CRAN.R-project.org/package=rms, version 6.6.0) package was used to establish a nomogram using independent prognostic factors identified by multivariate Cox regression analysis to predict 1-, 3-, and 5-year overall survival of patients. To assess the accuracy of the predictions, a calibration curve was plotted to observe the relationship between the predicted probability and the actual incidence. For each patient, three lines were drawn upward to determine the points received from the predictors in the nomogram. The sum of these points was located on the “Total points” axis. A line was drawn downward to assess the likelihood of 1-, 3-, and 5-year overall survival.

### 2.6 Immune cell infiltration

The CIBERSORT software [24] was applied to calculate the relative proportions of the 22 immune cells in the samples. CIBERSORT can describe the composition of immune infiltrating cells using the 547 preset barcode genes in the deconvolution algorithm based on the gene expression matrix. The immune score of samples was calculated using the “estimate” function package (https://R-Forge.R-project.org/projects/estimate/).

### 2.7 Screening of transcription factors correlated with the GJB3 expression

In TCGA-LUAD mRNA dataset, the highly expressed transcription factors were filtered based on the expression read counts median >1 and mean >10. The significantly differentially expressed transcription factors were selected using the DEGs between LUAD and normal samples according to Log_2_FC > 1 and FDR < 0.05. The correlation of transcription factors with *GJB3* mRNA was calculated using Pearson correlation, and the transcription factors significantly correlated with *GJB3* were screened according to *P* < 0.05 and Rho > 0.3.

### 2.8 Protein–protein interaction (PPI) network analysis

The STRING (https://string-db.org/, version 11.0) database [25] was employed to analyze the functional links between proteins. The Cytoscape (version 3.7.2) [26] was applied to visualize the PPI network.

### 2.9 Statistical analysis

Drug sensitivity was predicted using the “oncopredict” package [27] in the R language. The differences in gene expression and immune cell infiltration between groups were compared by the Wilcoxon rank sum test. Pearson correlation analysis was performed using the R language “cor” function. *P* < 0.05 were considered statistically significant.

## 3 Results

### 3.1 GJB3 was highly expressed in LUAD patients and correlated with LUAD stages

**Figure 1:**
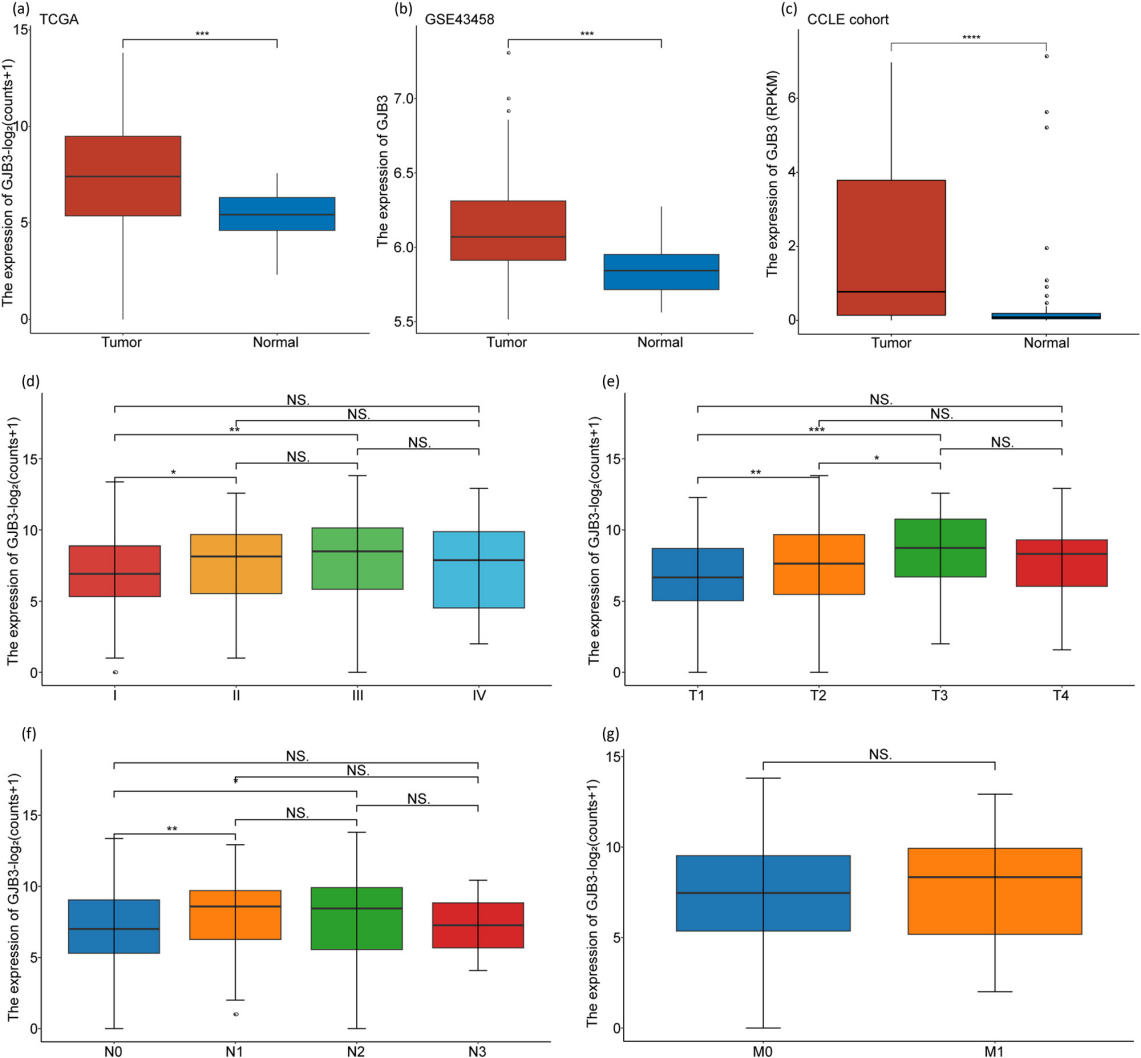
*GJB3* was highly expressed in LUAD patients. (a and b) The expression of *GJB3* between LUAD samples and normal samples in TCGA and GSE43458 cohorts. (c) The expression of *GJB3* between LUAD cells and normal cells in the CCLE cohort. (d) The expression of *GJB3* in different pathological stages of LUAD (stage I–stage Ⅳ). (e–g) The expression of *GJB3* in TNM classification of LUAD patients. The Wilcoxon rank-sum test was used to compare gene expression between two groups. A value of *P* < 0.05 was taken to indicate statistical significance.

In the TCGA and GSE43458 cohorts, we found that the *GJB3* was up-regulated in LUAD samples (LUAD vs normal, Figure 1a and b). In CCLE database, the *GJB3* was also highly expressed in lung cancer cells (Figure 1c). Next, we analyzed *GJB3* expression in different pathological stages and TNM stages of LUAD patients in the TCGA cohort. Compared to stage I, *GJB3* expression was increased in stage II and stage III (Figure 1d). Moreover, *GJB3* expression was differential between T1 and T2, T1 and T3, T2 and T3, N0 and N1, N0 and N2 (Figure 1e and f). However, there was no significant difference observed in the expression of *GJB3* between stage I and stage IV, T1 and T4, N0 and N3, which might be attributed to the relatively small sample size of patients with tumor metastasis and advanced LUAD. Additionally, the expression of *GJB3* displayed no significant difference between M0 and M1 (Figure 1g), indicating that the slightly decreased expression of *GJB3* in advanced stage was not seemingly linked to lung cancer metastasis. Moreover, in the GSE36471 dataset, we analyzed the expression of *GJB3* in different stages of LUAD patients, and obtained the same results as TCGA cohort (Figure S1).

### 3.2 LUAD patients with high GJB3 expression exhibited a worse prognosis

**Figure 2:**
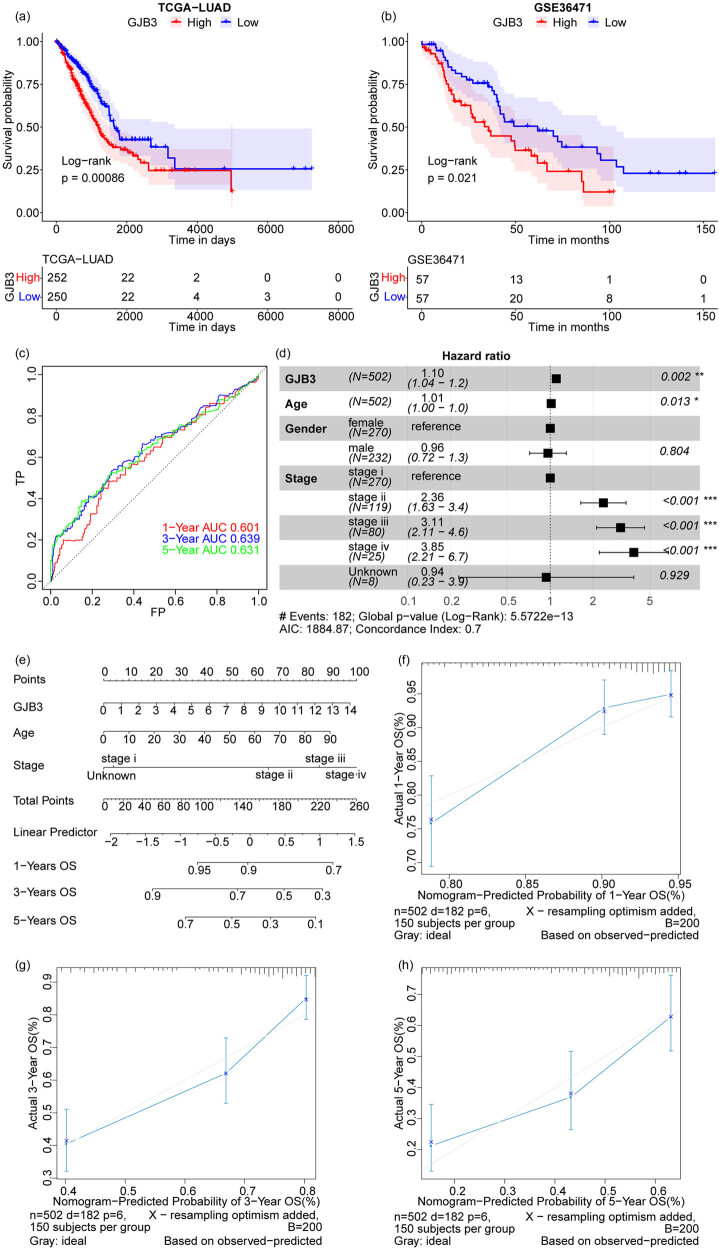
Patients with high *GJB3* expression exhibited worse prognosis in LUAD. (a and b) The KM survival curves of *GJB3* ^high^ and *GJB3* ^low^ groups in TCGA and GSE36471 cohorts. (c) The AUC for overall survival at 1-, 2-, 3-, and 5-year. (d) Forest plot of multivariate Cox regression analysis in TCGA cohort. (e) The nomogram model. (f–h) The calibration curves for predicting the probability of 1-, 3-, and 5-year overall survival in LUAD patients. The Wilcoxon rank-sum test was used to compare gene expression between two groups. A value of *P* < 0.05 was taken to indicate statistical significance.

As shown in Figure 2a and b, the *GJB3* ^high^ group was associated with a worse prognosis of LUAD patients compared to *GJB3* ^low^ group in both TCGA and GSE36471 cohorts. The time-dependent receiver operating characteristic curve analysis showed that the area under the curve (AUC) for overall survival at 1, 2, 3, and 5 years was 0.601, 0.639, and 0.631, respectively (Figure 2c). In TCGA cohort, the multivariate Cox regression analysis including *GJB3*, gender, and stage showed that *GJB3* could be an independent predictor of prognosis in LUAD patients (Figure 2d). Next, we constructed a nomogram model using *GJB3*, age, and stage (Figure 2e). The calibration curves of 1, 3, and 5 years were closer to the ideal curve (a 45° line passing through the origin of the coordinate axis with a slope of 1) in the calibration map, which indicated that the nomogram model predicted outcomes at 1, 3, and 5 years were consistent with the actual outcomes (Figure 2f–h).

### 3.3 Pathways correlated with GJB3 in LUAD

**Figure 3:**
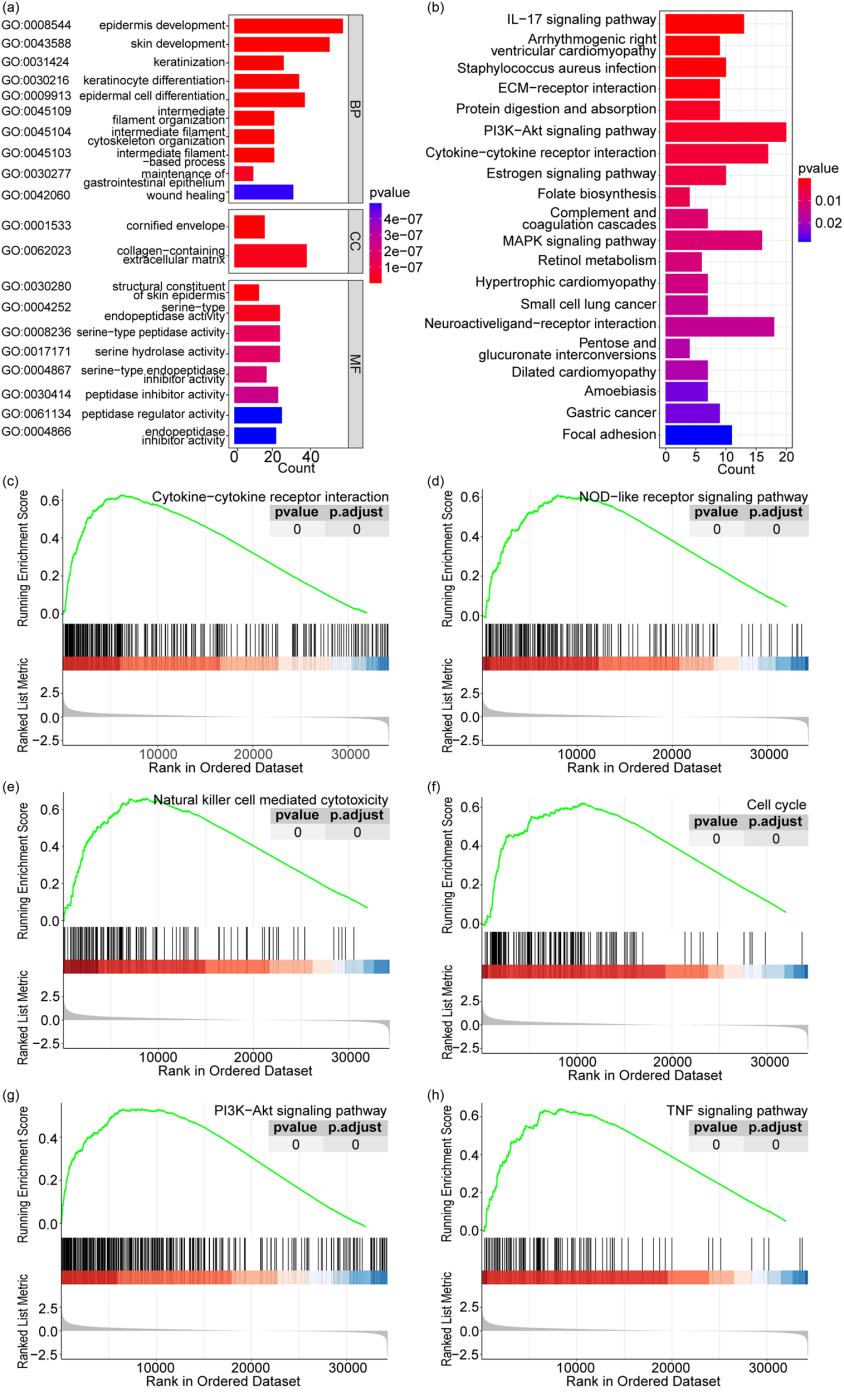
Pathways correlated with *GJB3* in LUAD. The top 20 significantly enriched GO (a) and KEGG pathways (b) of 602 DEGs. BP: biological process; CC: cellular component; MF: molecular function. (c) Cytokine–cytokine receptor interaction. (d) NLR signaling pathway. (e) Natural killer cell mediated cytotoxicity. (f) Cell cycle. (g) PI3K-Akt signaling pathway. (h) TNF signaling pathway.

In the TCGA cohort, we identified 602 DEGs between *GJB3* ^high^ and *GJB3* ^low^ groups. Enrichment analysis showed that these DEGs were significantly enriched in 217 GO and 25 KEGG pathways (Table S1). The top 20 GO terms (including 10 BP, 2 CC, and 8 MF) and KEGG pathways are presented in Figure 3a and b, respectively. Moreover, GSEA showed that a total of 164 pathways were significantly activated in *GJB3* ^high^ group compared to *GJB3* ^low^ group (Table S2). Among these 164 pathways, multiple pathways, such as cytokine–cytokine receptor interaction, nucleotide-binding oligomerization domain (NOD)-like receptor (NLR) signaling pathway, and natural killer cell-mediated cytotoxicity were correlated with the progression of tumors (Figure 3c–h).

### 3.4 Nine transcription factors were positively correlated with GJB3 in LUAD

**Figure 4:**
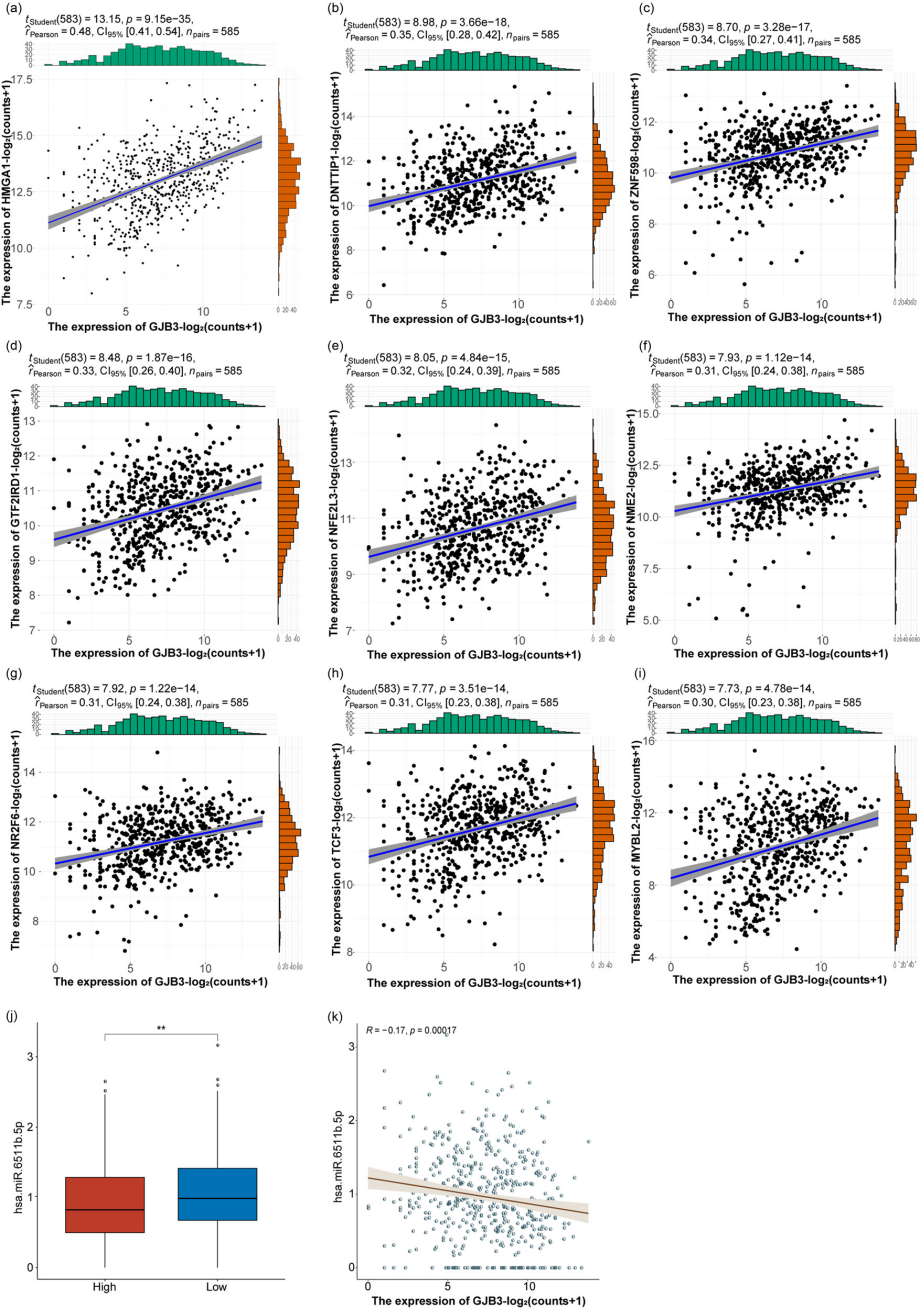
Nine transcription factors were positively correlated with *GJB3*, and the expression of *GJB3* was regulated by hsa-miR-6511b-5p in LUAD. (a–i) The correlation of nine transcription factors of *GJB3*. (j) The expression of hsa-miR-6511b-5p in *GJB3* ^high^ and *GJB3* ^low^ groups. (k) The correlation of hsa-miR-6511b-5p expression and *GJB3* expression.

We identified 55 transcription factors that were differentially expressed and more highly expressed in LUAD and calculated the correlation of 55 transcription factors with *GJB3* expression. We found that the expressions of nine transcription factors were positively associated with *GJB3* expression (Table 1, Figure 4a–i).

**Table 1:** Transcription factors associated with *GJB3* expression in LUAD patients

Transcription factors	Rho	*P*-value
HMGA1	0.478233283	9.15 × 10^−35^
DNTTIP1	0.348650593	3.66 × 10^−18^
ZNF598	0.339133563	3.28 × 10^−17^
GTF2IRD1	0.331320183	1.87 × 10^−16^
NFE2L3	0.316108808	4.84 × 10^−15^
NME2	0.312032456	1.12 × 10^−14^
NR2F6	0.311606081	1.22 × 10^−14^
TCF3	0.306398541	3.51 × 10^−14^
MYBL2	0.304855148	4.78 × 10^−14^

### 3.5 GJB3 expression might be regulated by hsa-miR-6511b-5p in LUAD

We identified 35 targeted miRNAs of *GJB3* (Table S3) utilizing the mirDIP database (http://ophid.utoronto.ca/mirDIP/). In TCGA-LUAD, we investigated the expression of 35 miRNA in *GJB3* ^high^ and *GJB3* ^low^ groups. The hsa-miR-6511b-5p expression was remarkably differential between *GJB3* ^high^ and *GJB3* ^low^ groups (Figure 4j) and exhibited a significantly negative correlation with *GJB3* expression (Figure 4k). These results indicated that *GJB3* expression might be regulated by hsa-miR-6511b-5p in LUAD.

### 3.6 GJB3 was correlated with the immune cell infiltration and immune checkpoint expression in LUAD

**Figure 5:**
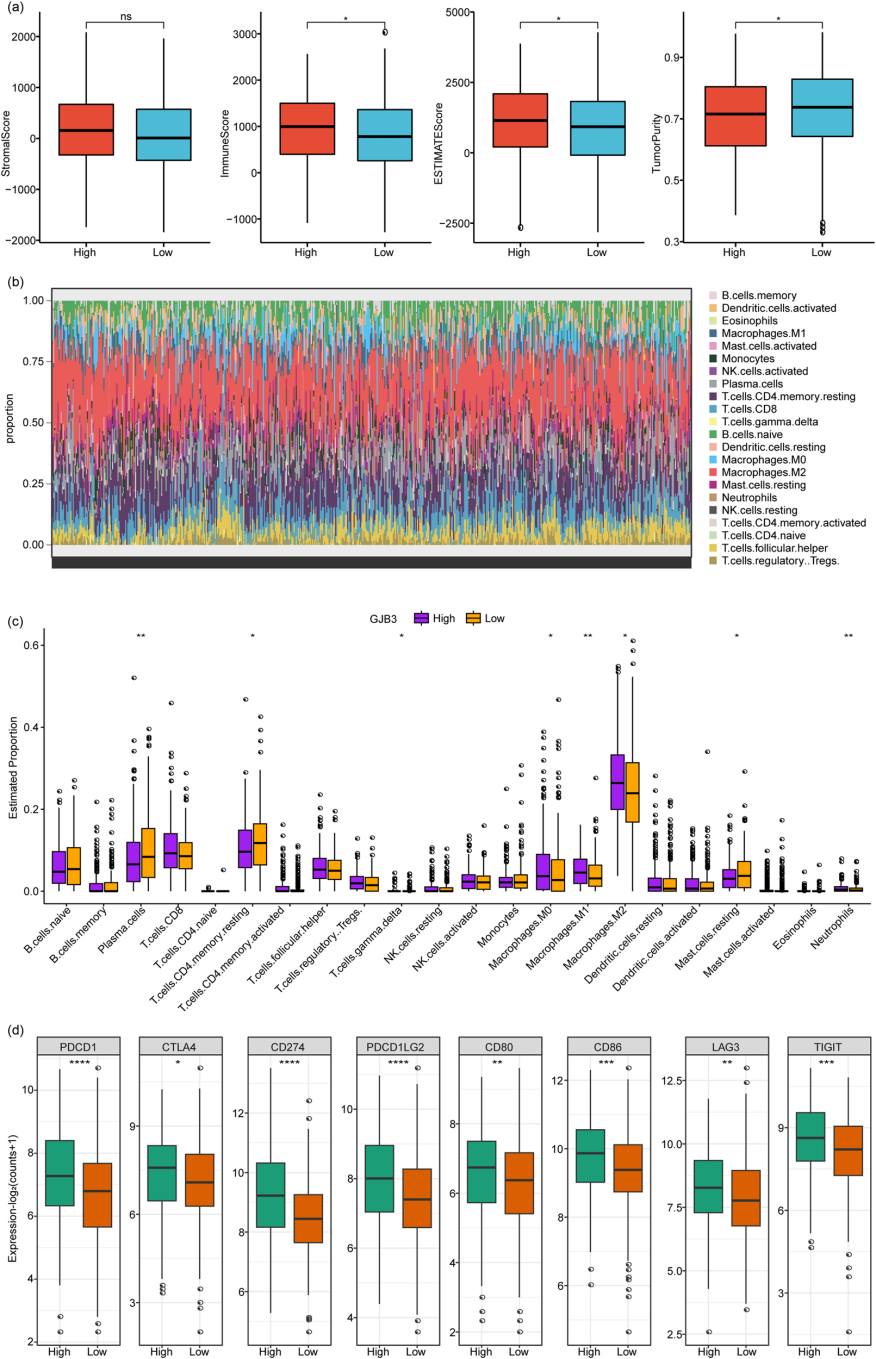
*GJB3* was associated with the immune cell infiltration and immune checkpoint expression in LUAD. (a) The level of StromalScore, ImmuneScore, ESTIMATEScore, and TumorPurity in *GJB3* ^high^ and *GJB3* ^low^ groups. (b) The relative proportions of 22 immune cell infiltration. (c) The level of immune cell infiltration in *GJB3* ^high^ and *GJB3* ^low^ groups. (d) The expression of PD-1 (PDCD1), CTLA-4, PD-L1 (CD274), PD-L2 (PDCD1LG2), CD80, CD86, LAG-3, and TIGIT in *GJB3* ^high^ and *GJB3* ^low^ groups. PD-1 (PDCD1) was immune checkpoint marker of T cells, neutrophils, NK cells, NKT cells, monocytes, DCs, B cells. PD-L1 and PD-L2 were ligands for PD-1. CTLA-4 was immune checkpoint marker of T cells. CD80 and CD86 were ligands for CTLA-4. LAG-3 was immune checkpoint marker of T cells, NK cells, DC cells, and B cells. TIGIT was immune checkpoint marker of T cells, NK cells, monocytes, and neutrophils.

Subsequently, we found that the ESTIMATE Score and Immune Score levels were observably increased and Tumor Purity level was remarkably decreased in *GJB3* ^high^ group than that in *GJB3* ^low^ group (Figure 5a). In addition, we calculated the relative proportions of 22 immune cell infiltration (Figure 5b) and analyzed the level of immune cell infiltration in *GJB3* ^high^ and *GJB3* ^low^ groups, and found that the levels of Plasma.cells, T.cells.CD4.memory.resting, T.cells.gamma.delta, Macrophages.M0, Macrophages.M1, Macrophages.M2, Mast.cells.restin, and Neutrophils were observably differential between in *GJB3* ^high^ and *GJB3* ^low^ groups (Figure 5c). Figure 5d shows that the expression of PD-1 (PDCD1), CTLA-4, PD-L1 (CD274), PD-L2 (PDCD1LG2), CD80, CD86, LAG-3, and TIGIT were significantly increased in *GJB3* ^high^ group than that in *GJB3* ^low^ groups.

### 3.7 GJB3 was correlated with drug sensitivity in LUAD

**Figure 6:**
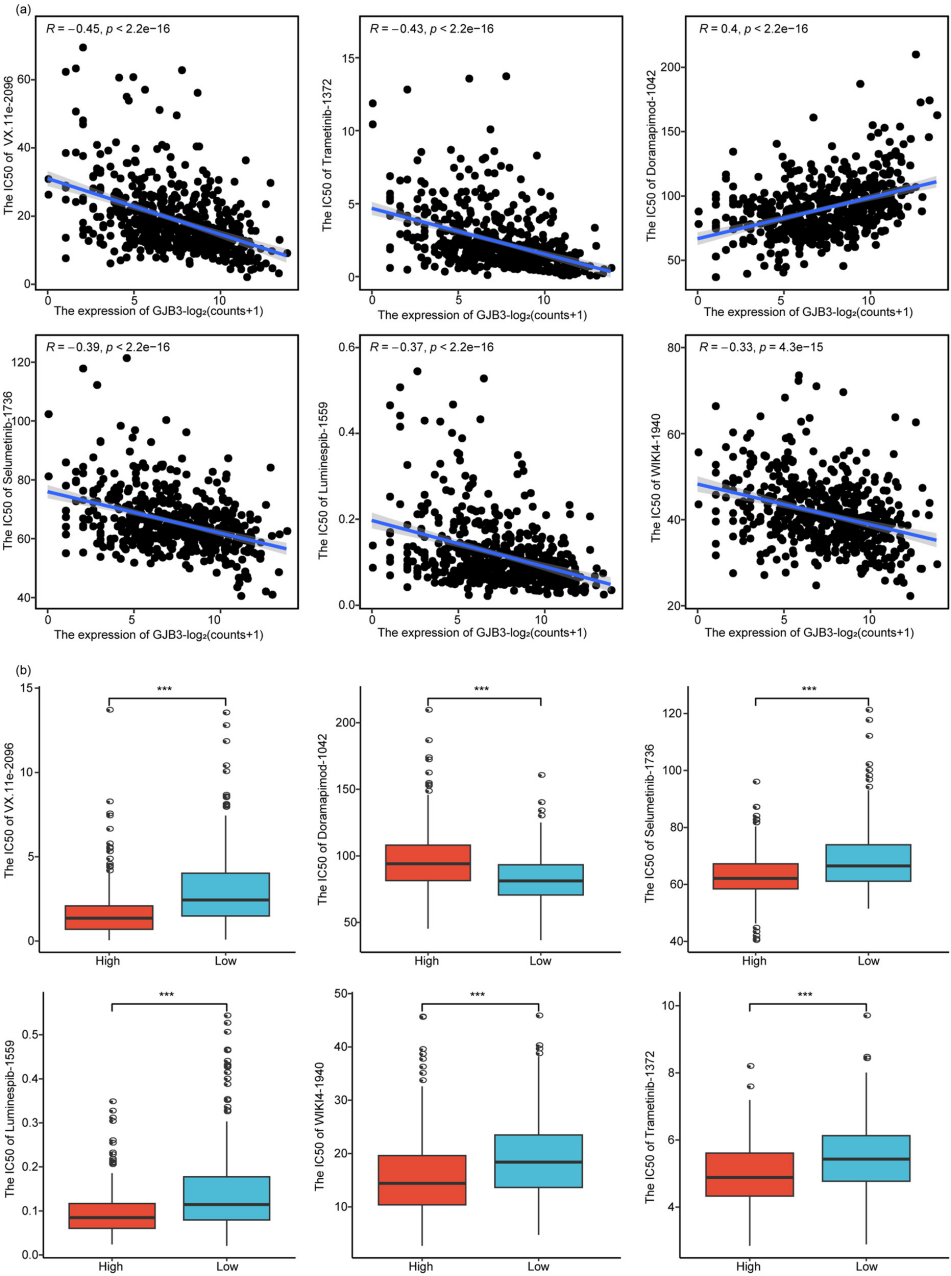
*GJB3* was correlated with drug sensitivity in LUAD. (a) The correlation of six drugs with *GJB3*. (b) The IC50 of six drugs in *GJB3* ^high^ and *GJB3* ^low^ groups.

Finally, we analyzed the correlation of *GJB3* with drug sensitivity (IC50 values of drugs) using the R language “oncoPredict” package [27]. The results showed that *GJB3* exhibited a significantly negative correlation with 86 drugs and a positive association with 53 drugs (Table S4). We selected the six drugs in which the absolute value of the correlation coefficient was greater than 0.3 (Figure 6a) and compared their IC50 between *GJB3* ^high^ and *GJB3* ^low^ groups. We found that the IC50 of negatively related drugs such as VX.11e_2096 was lower in *GJB3* ^high^ group compared to *GJB3* ^low^ group, while the positively related drugs were higher (Figure 6b). These results indicated that *GJB3* could be a promising target of lung cancer therapy.

## 4 Discussion

LUAD is a markedly heterogeneous entity and it has the highest incidence among NSCLC and is characterized by various histologic subtypes, a high rate of metastasis, and recurrence [28,29]. Common symptoms of LUAD include dyspnea, chest pain, cough, and weight loss. Previous studies have indicated that aberrant expression of genes was also important mutagenic factors of LUAD [30]. For example, the level of *NPM1* expression was remarkably increased in LUAD patients and was correlated with a prognosis of LUAD patients [31]. The expression of *FGL2* was lower in LUAD, and the patients with high *FGL2* expression displayed a better prognosis. Moreover, the high *FGL2* expression was associated with the infiltration of immune cells in LUAD [32]. *GJB3* was initially mapped to chromosome 1p35-p33, and its mutations participated in the non-syndromic hearing loss and two major conditions, erythrokeratodermia variabilis et progressive [16,17]. Moreover, several studies have suggested that *GJB3* is a tumor suppressor in many cancers. However, the potential roles of *GJB3* in LUAD were largely unknown. Thus, we explored the potential effects of *GJB3* in TME, immunotherapy, and drug sensitivity in LUAD, and found that the *GJB3* was highly expressed in LUAD patients, and the patients with high *GJB3* expression exhibited poor prognosis. Moreover, *GJB3* was associated with the immune cell infiltration, immune checkpoints expression, and drug sensitivity in LUAD.

First, we analyzed *GJB3* expression in LUAD samples in different pathological stages and TNM classification of LUAD patients. We found that the *GJB3* was up-regulated in LUAD patients, and *GJB3* was correlated with pathological stages and TNM classification. Although previous research has shown that *GJB3* expression was increased in PDAC liver metastasis [21], however, there was no significant difference observed in the expression of *GJB3* between stage I and stage IV, T1 and T4, N0 and N3, M0 and M1, which might be attributed to the small sample size of advanced LUAD patients. In addition, the patients with high *GJB3* expression exhibited a worse prognosis in LUAD. In PDAC liver metastatic mice, the GJB3 overexpression was correlated with poor prognosis, and *GJB3* knockdown could alleviate the liver metastasis [21]. These pieces of evidence showed that *GJB3* might be a cancer-promoting gene in LUAD. However, *GJB3* expression was decreased in thyroid cancer samples, and *GJB3* overexpression could reduce the proliferation, migration, and invasion of thyroid cancer cells [20]. In metastatic breast cancer of bone, the patients with high *GJB3* expression exhibit better prognosis [19]. Accordingly, *GJB3* might serve as a cancer-promoting gene in adenocarcinoma and as a tumor suppressor gene in certain cancers, such as thyroid cancer and breast cancer. We also found that *GJB3* expression was positively correlated with nine transcription factors. Among these, HMGA1 [33], NME2 [34], NR2F6 [35], and MYBL2 [36] were found to be highly expressed in LUAD patients, and high expression of HMGA1 [37], NME2 [34], and MYBL2 [38] was correlated with inferior prognosis of LUAD patients. Accordingly, HMGA1, NME2, and MYBL2 act as oncogenic transcription factors in LUAD. It has been reported that silencing HMGA1 significantly reduced cell proliferation and glycolysis while promoting cell apoptosis in LUAD [33]. Overexpression of HMGA1 strongly stimulated LUAD cell growth and metastasis, while silencing HMGA1 inhibited LUAD cell growth and metastasis [39]. It was demonstrated that the overexpressing miR-26a in a LUAD cell line inhibited cell migration, invasion, and proliferation by targeting HMGA1 [40]. MYBL2 acts as a transcriptional activator in LUAD cells [41]. It might control cell cycle genes by binding to the promoters of highly expressed genes in LUAD cells and cooperating with FOXM1 [41]. Furthermore, MYBL2 was shown to interact with the LOXL1-AS1 promoter, and MYBL2 knockdown may counterbalance miR-423-5p repression-mediated increase in the progression of LOXL1-AS1 downregulated LUAD cells [42]. We also found that *GJB3* might be regulated by hsa-miR-6511b-5p in LUAD. Some recent studies also suggested the regulator role of hsa-miR-6511b-5p in different cancers. In colorectal cancer, hsa-miR-6511b-5p could inhibit cancer cell migration and invasion through methylation of CD44 by targeting BRG1 [43]. In lymphoma, LncHOTAIR overexpression promoted the progression of cancer via regulating the hsa-miR-6511b-5p/ATG7 axis [44]. Moreover, high expressions of miR-6511b-5p at 6 h after the return of spontaneous circulation predicted poor outcomes in cardiac arrest patients [45]. Considering that GJB3 was highly expressed in LUAD and positively correlated with transcription factors HMGA1 and MYBL2, we hypothesized that hsa-miR-6511b-5p might impact the progression of LUAD via modulating the *GJB3*/HMGA1 or *GJB3*/MYBL2 pathways. Further investigation at cellular or clinical levels is required to validate this hypothesis.

The results of GO enrichment analysis revealed that DEGs between *GJB3* ^high^ and *GJB3* ^low^ groups were significantly enriched in the collagen-containing extracellular matrix (ECM). This ECM plays a critical role in the TME, with alterations in ECM being a key feature of the dysregulated microenvironment in lung cancer [46]. Collagens are the most abundant ECM proteins in the lung [47]. The abnormal production of collagen, posttranslational alterations affecting its stiffness, and the expression of collagen receptors provide a favorable microenvironment for the progression of lung cancer [46]. Studies have shown that high expression of collagen I in LUAD patients is associated with a shorter survival rate [48]. Therefore, it is suggested that GJB3 may have a significant impact on regulating BP related to collagens in LUAD. Additionally, GSEA showed that a total of 164 pathways were significantly activated in the *GJB3* ^high^ group compared to the *GJB3* ^low^ group, such as cytokine–cytokine receptor interaction, NLR signaling pathway, natural killer cell mediated cytotoxicity, PI3K-AKT signaling pathway, and TNF signaling pathway. Cytokines are important regulators and mobilizers. They could regulate the growth, proliferation differentiation, activation, and homeostasis of immune cells by binding to the corresponding receptors of cytokines [49,50]. In cancer, the cytokine receptor expression is increased, which can promote the immune evasion of tumor cells [51]. Cytokine–cytokine receptor interaction is a way in which cytokines respond to activation signals induced, and it has been demonstrated to play a critical role in tumors, especially in glioblastoma [49,52,53]. In addition, Zheng et al. have found that the hot LUAD tumors were significantly enriched in cytokine–cytokine receptor interaction [54]. NLRs play a major role in the interface between innate immunity and cancer in humans. NLR signaling pathway is also involved in the autophagy and apoptosis of inflammation-associated cancer [55]. The NLRs are categorized into five subfamily members: NLRA (CIITA), NLRB (NAIP), NLRC (NOD1, NOD2), NLRP (NLRP3), and NLRX (NLRX1) [56]. NAIP could inhibit the apoptosis induced by varieties of signals via the inactivation of CASP3, CASP7, and CASP9 [57]. NLRP3 abnormal activation was associated with inflammatory diseases. In LUAD, NLRP3 inflammasome activation could promote nicotine-induced cell proliferation and migration [58]. The NOD1 expression in T cells could impede the colitis-associated intestinal tumorigenesis through mediating the IFN-γ signaling [59], and NOD2 could suppress the tumorigenesis of colitis-associated cancer by downregulating NF-κB and MAPK pathways with the induction of IRF4 [60]. Wang et al. have suggested that the descended *NOD2* expression was correlated with poor prognosis of LUAD, and the reduction of *NOD2* expression in macrophages promotes the conversion of the M1 macrophages to M2 macrophages in LUAD [61]. These suggested that the different members of the NLRs signaling pathway play different functions in LUAD, implying that NLRs signaling pathway was activated in LUAD patients with high *GJB3* expression was reasonable. Moreover, natural killer cell mediated cytotoxicity and PI3K-AKT signaling pathway were also associated with inflammatory process immune of tumors [62–64]. Collectively, these findings showed that the *GJB3* might be involved in the immune microenvironment of LUAD by regulating multiple inflammation and immune-related pathways.

We also found that the levels of Plasma.cells, T.cells.CD4.memory.resting, T.cells.gamma.delta, Macrophages.M0, Macrophages.M1, Macrophages.M2, Mast.cells.restin, and Neutrophils were observably differential between in *GJB3* ^high^ and *GJB3* ^low^ groups. T cells were divided into CD4+T cells and the CD8+T cells [65]. CD4+T cells are auxiliary T cells, while CD8+T cells are cytotoxic T lymphocytes. CD4+T cells included several subsets, such as T helper 1 (Th1), Th2, Th9, and regulatory T cells (Tregs), CD4+T cells could indirectly involve in clearing infection via regulating the activity of macrophages, neutrophils, B cells, and other immune cells [66]. Tumor-associated macrophage infiltration plays critical roles in tumorigenesis and metastasis. Normally, macrophages contain two phenotypes (M1 macrophages and M2 macrophages). M1 macrophages are induced via the activation of lipopolysaccharide and could express pro-inflammatory cytokines. M2 macrophages are induced through IL-4 expression and could express anti-inflammatory cytokines. M1 macrophages could promote cancer cell elimination to restrain tumor progression, and M2 macrophages can induce an immunosuppressive effect to promote tumor progression [67]. The polarization of these two macrophages is a highly dynamic process. It has been found that about 85% of the macrophages in the tumor stroma are the M2 macrophages in the NSCLC patients with an average of 1-year survival [68]. M1 and M2 macrophages were significantly elevated in the NSCLC tissue than in the normal lung tissue [69]. Wang et al. discovered that the proportion of M1 macrophages was significantly higher in LUAD compared to normal tissues. Furthermore, they found that the high level of M1 macrophages was correlated with a poorer prognosis in patients with LUAD [70]. However, Jackute et al. have demonstrated that a higher level of infiltrating M2 macrophages was also correlated with inferior NSCLC prognosis [69]. In this study, we found that the high infiltration of M1 and M2 macrophages was associated with a poor prognosis of LUAD patients with high *GJB3* expression. One probable explanation might be that tumor cells release monocyte chemotactic protein-2, which can recruit circulating M1 macrophages to the tumor location [71,72]. Moreover, abnormal gene expression also could increase the infiltration of M1-type macrophage in tumor [73]. Noteworthily, previous research has been divided on the relevance of M1 macrophages in NSCLC patient survival. Welsh and colleagues have found that the density of macrophage had positive correlation with survival of patient in the tumor islets [74]. Chen et al. have discovered that high density of tumor-infiltrating macrophages may increase NSCLC angiogenesis and cause adverse outcome [75]. Dai et al. have demonstrated that macrophages in the NSCLC islets were positively associated with survival, whereas in the tumor stroma macrophages were negatively associated with survival [68]. However, some studies showed that there was no association between the macrophage number and prognosis of NSCLC patients [76,77]. The immune cell infiltration results we observed might be the product of complex interactions among all members in TME, while deepening details should be further mined in the future.

The *GJB3* might involve in the immune cell infiltration in LUAD. In addition, immune checkpoints, as negative regulators in the immune system, participated in the prevention of autoimmunity and protected tissues from immune damage [78]. Recently, the immunotherapy targeting immune checkpoint blockers, such as CTLA-4, PD-1, and PD-L1, changed the landscape of LUAD treatments [79]. The expressions of PD-1 (PDCD1), CTLA-4, PD-L1 (CD274), PD-L2 (PDCD1LG2), CD80, CD86, LAG-3, and TIGIT were significantly increased in *GJB3* ^high^ group than that in *GJB3* ^low^ group. These results suggested that *GJB3* expression was closely correlated with immunotherapy in LUAD. Finally, we found that the *GJB3* exhibited a significantly negative correlation with 86 drugs and a positive association with 53 drugs. LUAD cells are able to rapidly acquire drug resistance after initial treatment and usually cannot be treated with chemotherapeutic agents [14,15]. Xu et al. have indicated that *GJB3* expression was decreased in papillary thyroid cancer, and the ginsenoside could increase *GJB3* expression, thereby suppressing the proliferation and migration of thyroid cancer cells [20]. These studies suggested that the *GJB3* might be a drug resistance related gene in LUAD, which warrants further exploration in future studies.

Although our study had presented the role of GJB3 in the prognosis and immune microenvironment of LUAD patients, it is important to acknowledge some limitations. First, we did not explore the mechanism by which GJB3 regulates immune cell infiltration in LUAD. Second, further investigation and exploration of the role of GJB3 in the prognosis of LUAD patients should be conducted through experimental clinical trials.

## 5 Conclusion

In summary, we found that the *GJB3* was highly expressed in LUAD patients, and the patients with high *GJB3* expression exhibited poor prognoses. Moreover, the *GJB3* was associated with immune cell infiltration, immune checkpoint expression, and drug sensitivity in LUAD. Our findings might provide valuable information for further exploring the pathogenesis mechanism of LUAD and indicate that *GJB3* might serve as a promising molecular target for the diagnosis and treatment of LUAD.

## Abbreviations

AUC: area under the curve
CCLE: Cancer Cell Line Encyclopedia
CNV: copy number variation
Cx31: Connexin 31
DEGs: differentially expressed genes
ECM: extracellular matrix
*GJB3*
: gap junction protein beta 3
GSEA: gene set enrichment analysis
LUAD: lung adenocarcinoma
NOD: nucleotide-binding oligomerization domain
NSCLC: non-small cell lung cancer
PDAC: pancreatic ductal adenocarcinoma
PPI: protein–protein interaction
SCLC: small cell lung cancer
TCGA: The Cancer Genome Atlas
Th1: T helper 1
TME: tumor microenvironment
Tregs: regulatory T cells

## Supplementary Material

supplementary material
