# Supplementary material for: The GJB3 correlates with the prognosis, immune cell infiltration, and therapeutic responses in lung adenocarcinoma
Source: Open Med (Wars). 2024 Aug 10;19(1):20240974. doi: 10.1515/med-2024-0974 (PMC11317640; doi:10.1515/med-2024-0974)
Supplement: supplementary material [file med-2024-0974-sm.pdf]

Supplementary material

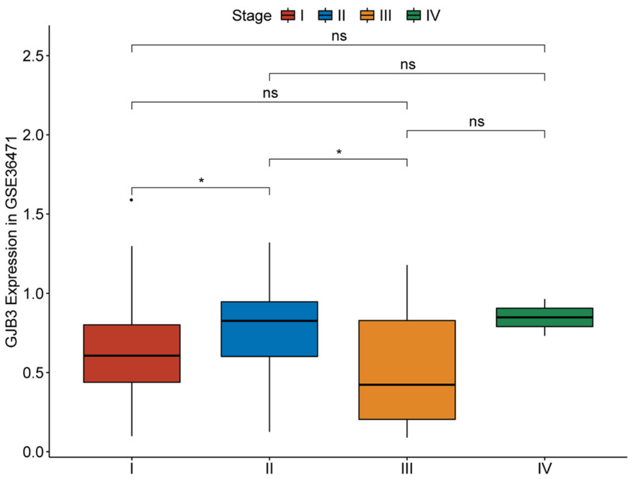

Figuer S1: The expression of GJB3 in different stages of LUAD patients in the GSE36471 dataset.

Table S1: The results of GO and KEGG enrichment analysis

	ONTOLOGY	ID	Description	Gene Ratio	Bg Ratio	*p* value	*q* value	gene ID	Count
GO:0008544	BP	GO:0008544	epidermis development	57/ 498	355/ 18800	9.06×10^{-25}	7.96×10^{-25}	GJB5/LAMC2/ KRT16/KRT6A/ KRT6B/LAMB3/ CD109/KRT6C/ ANXA1/CDH3/ SPRR1B/KRT17/ TGM5/COL17A1/ LIPK/PLEC/KRT8/ KRT7/EPHA2/ SPRR1A/KRT78/ COL7A1/SPRR3/ DKK1/EREG/SFN/ MSX2/FERMT1/ SPRR2D/KRT80/ KRT14/CST6/ LAMA3/SULT2B1/ SPRR2A/IL1A/ KRT81/KLK7/ SPRR2F/S100A7/ SCEL/WNT10A/IVL/ FOXQ1/ABCA12/ KRT15/KRT5/SOX9/ BNC1/FOXE1/GAL/ KLK5/CASP14/ KRT75/KLK14/ SLITRK6/USH1C	57
GO:0043588	BP	GO:0043588	skin development	50/ 498	296/ 18800	1.04×10^{-22}	9.1×10^{-23}	GJB3/KRT16/ KRT6A/ITGB4/ KRT6B/CD109/ KRT6C/ANXA1/ CDH3/SPRR1B/ KRT17/LIPK/PLEC/ KRT8/KRT7/EPHA2/ SPRR1A/KRT78/ SPRR3/DKK1/EREG/ SFN/ITGA3/ITGA6/ MSX2/ITGA2/ FERMT1/SPRR2D/ KRT80/ALOXE3/ SPRR2A/MET/IL1A/	

(Continued)

Table S1: *Continued*

	ONTOLOGY	ID	Description	Gene Ratio	Bg Ratio	*p* value	*q* value	gene ID	Count
								CLDN1/KRT81/SPRR2F/S100A7/SCEL/WNT10A/IVL/FOXQ1/ABCA12/KRT5/SOX9/FOXE1/GAL/KLK5/CASP14/KRT75/DACT2	
GO:0031424	BP	GO:0031424	keratinization	26/498	85/18800	8.44×10^{-18}	7.42×10^{-18}	KRT16/KRT6A/KRT6B/KRT6C/CDH3/SPRR1B/KRT17/LIPK/KRT8/KRT7/SPRR1A/KRT78/SPRR3/SFN/SPRR2D/KRT80/SPRR2A/IL1A/KRT81/SPRR2F/IVL/ABCA12/KRT5/KLK5/CASP14/KRT75	26
GO:0030216	BP	GO:0030216	keratinocyte differentiation	34/498	167/18800	1.08×10^{-17}	9.48×10^{-18}	KRT16/KRT6A/KRT6B/CD109/KRT6C/ANXA1/CDH3/SPRR1B/KRT17/LIPK/PLEC/KRT8/KRT7/EPHA2/SPRR1A/KRT78/SPRR3/EREG/SFN/MSX2/SPRR2D/KRT80/SPRR2A/IL1A/KRT81/SPRR2F/S100A7/SCEL/IVL/ABCA12/KRT5/KLK5/CASP14/KRT75	34
GO:0009913	BP	GO:0009913	epidermal cell differentiation	37/498	230/18800	7.32×10^{-16}	6.43×10^{-16}	KRT16/KRT6A/KRT6B/CD109/KRT6C/ANXA1/CDH3/SPRR1B/KRT17/LIPK/PLEC/	37

(Continued)

Table S1: *Continued*

	ONTOLOGY	ID	Description	Gene Ratio	Bg Ratio	*p* value	*q* value	gene ID	Count
								KRT8/KRT7/EPHA2/SPRR1A/KRT78/SPRR3/EREG/SFN/MSX2/SPRR2D/KRT80/SULT2B1/SPRR2A/IL1A/KRT81/SPRR2F/S100A7/SCEL/IVL/ABCA12/KRT5/KLK5/CASP14/KRT75/SLITRK6/USH1C	
GO:0045109	BP	GO:0045109	intermediate filament organization	21/498	68/18800	2.04×10^{-14}	1.79×10^{-14}	KRT16/KRT6A/KRT6B/KRT6C/KRT19/PKP2/KRT17/PLEC/KRT8/KRT7/KRT78/KRT80/KRT14/KRT81/KRT23/KRT13/KRT15/KRT5/KRT75/KRT20/PKP1	21
GO:0045104	BP	GO:0045104	intermediate filament cytoskeleton organization	21/498	88/18800	5.67×10^{-12}	4.98×10^{-12}	KRT16/KRT6A/KRT6B/KRT6C/KRT19/PKP2/KRT17/PLEC/KRT8/KRT7/KRT78/KRT80/KRT14/KRT81/KRT23/KRT13/KRT15/KRT5/KRT75/KRT20/PKP1	21
GO:0045103	BP	GO:0045103	intermediate filament-based process	21/498	89/18800	6.33×10^{-12}	5.56×10^{-12}	KRT16/KRT6A/KRT6B/KRT6C/KRT19/PKP2/KRT17/PLEC/KRT8/KRT7/KRT78/KRT80/KRT14/KRT81/KRT23/	21

(Continued)

Table S1: *Continued*

	ONTOLOGY	ID	Description	Gene Ratio	Bg Ratio	*p* value	*q* value	gene ID	Count
								KRT13/KRT15/ KRT5/KRT75/ KRT20/PKP1	
GO:0030277	BP	GO:0030277	maintenance of gastrointestinal epithelium	10/ 498	22/ 18800	3.43×10^{-8}	3.01×10^{-8}	INAVA/SERPINA3/ VSIG1/SOX9/ NEUROD1/TFF1/ TFF2/MUC2/MUC4/ MUC13	10
GO:0010951	BP	GO:0010951	negative regulation of endopeptidase activity	26/ 498	251/ 18800	1.37×10^{-6}	1.2×10^{-6}	ANXA2/ANXA8/ CD109/PLAUR/ SERPINB5/ SERPINB7/ ANXA8L1/COL7A1/ SFN/SERPINA4/ BIRC3/SERPINA5/ CST6/SERPINB4/ SERPINE1/TIMP4/ SERPINA3/PI3/ SERPINA1/ SERPINB2/ SERPINB3/A2ML1/ SPOCK1/CST4/ CST1/AMBP	26
GO:0007586	BP	GO:0007586	digestion	19/498	137/ 18800	1.41×10^{-6}	1.24×10^{-6}	INAVA/UCN2/ KCNN4/NPSR1/ PRSS3/SERPINA3/ VSIG1/PRSS2/ SOX9/NEUROD1/ TFF1/TFF2/PRSS1/ MUC2/MUC4/ MUC13/PGC/ CHIA/SST	19
GO:0052547	BP	GO:0052547	regulation of peptidase activity	36/ 498	456/ 18800	1.87×10^{-6}	1.64×10^{-6}	ANXA2/ANXA8/ CD109/PLAUR/ PERP/SERPINB5/ PPARG/PRSS22/ SERPINB7/ ANXA8L1/COL7A1/ SFN/SERPINA4/	36

(Continued)

Table S1: *Continued*

	ONTOLOGY	ID	Description	Gene Ratio	Bg Ratio	*p* value	*q* value	gene ID	Count
								ASPH/XDH/BIRC3/SERPINA5/CST6/SERPINB4/S100A9/SERPINE1/TIMP4/SERPINA3/UMODL1/PI3/NLRP12/SERPINA1/SERPINB2/AIM2/S100A8/SERPINB3/A2ML1/SPOCK1/CST4/CST1/AMBP	
GO:0010669	BP	GO:0010669	epithelial structure maintenance	10/498	32/18800	1.87×10^{-6}	1.64×10^{-6}	INAVA/SERPINA3/VSIG1/SOX9/NEUROD1/TFF1/TFF2/MUC2/MUC4/MUC13	10
GO:0022617	BP	GO:0022617	extracellular matrix disassembly	13/498	62/18800	2×10^{-6}	1.76×10^{-6}	MELTF/MMP14/FSCN1/MMP11/MMP1/KLK7/MMP10/CTSV/MMP7/PRSS2/PRSS1/KLK5/MMP3	13
GO:0010466	BP	GO:0010466	negative regulation of peptidase activity	26/498	262/18800	2.25×10^{-6}	1.97×10^{-6}	ANXA2/ANXA8/CD109/PLAUR/SERPINB5/SERPINB7/ANXA8L1/COL7A1/SFN/SERPINA4/BIRC3/SERPINA5/CST6/SERPINB4/SERPINE1/TIMP4/SERPINA3/PI3/SERPINA1/SERPINB2/SERPINB3/A2ML1/SPOCK1/CST4/CST1/AMBP	26
GO:0052548	BP	GO:0052548	regulation of endopeptidase activity	34/498	426/18800	3.05×10^{-6}	2.68×10^{-6}	ANXA2/ANXA8/CD109/PLAUR/	34

(Continued)

Table S1: *Continued*

	ONTOLOGY	ID	Description	Gene Ratio	Bg Ratio	*p* value	*q* value	gene ID	Count
								PERP/SERPINB5/PPARG/SERPINB7/ANXA8L1/COL7A1/SFN/SERPINA4/ASPH/XDH/BIRC3/SERPINA5/CST6/SERPINB4/S100A9/SERPINE1/TIMP4/SERPINA3/PI3/NLRP12/SERPINA1/SERPINB2/AIM2/S100A8/SERPINB3/A2ML1/SPOCK1/CST4/CST1/AMBP	
GO:0001894	BP	GO:0001894	tissue homeostasis	26/498	272/18800	4.31×10^{-6}	3.79×10^{-6}	SLC2A1/IL20RB/CDH3/OAS1/INAVA/NXNL2/CLDN1/SERPINA3/ADGRV1/ABCA12/ZG16B/ABCC8/VSIG1/HOXA13/CALCA/SOX9/NEUROD1/TFF1/TFF2/PIP/GJB6/MUC2/CST4/MUC4/MUC13/USH1C	26
GO:0060249	BP	GO:0060249	anatomical structure homeostasis	28/498	319/18800	7.35×10^{-6}	6.46×10^{-6}	SLC2A1/IL20RB/CDH3/OAS1/INAVA/NXNL2/CLDN1/SERPINA3/CALB2/ADGRV1/ABCA12/ZG16B/ABCC8/VSIG1/HOXA13/CALCA/SOX9/NEUROD1/TFF1/TFF2/ADORA1/PIP/GJB6/MUC2/CST4/	28

(Continued)

Table S1: *Continued*

	ONTOLOGY	ID	Description	Gene Ratio	Bg Ratio	*p* value	*q* value	gene ID	Count
								MUC4/MUC13/ USH1C	
GO:0042060	BP	GO:0042060	wound healing	31/498	429/ 18800	0.000104	9.15×10^{-5}	KRT6A/ANXA8/ CD109/PLAUR/ ANXA1/CDH3/ PLAU/PPARG/ PLEC/WNT7A/ SPRR3/EREG/ MSX2/ITGA2/ FERMT1/ TNFRSF12A/F2RL1/ TGFA/SERPINE1/ IL1A/CLDN1/SAA1/ SERPINA1/ SERPINB2/S100A8/ F5/ABCC8/HNF4A/ SFTA3/DUOX2/ CYP4F11	31
GO:0030198	BP	GO:0030198	extracellular matrix organization	25/ 498	307/ 18800	0.000138	0.000121	MYO1E/LAMB3/ MELTF/SERPINB5/ COL17A1/MMP14/ FSCN1/TGFBI/ LOXL2/MMP11/ FERMT1/MMP1/ KLK7/MMP10/ C6orf15/CTSV/ MMP7/PRSS2/ SOX9/GREM1/ PRSS1/KLK5/ MMP3/IBSP/ COL11A1	25
GO:0051346	BP	GO:0051346	negative regulation of hydrolase activity	28/ 498	371/ 18800	0.000138	0.000121	ANXA2/ANXA8/ CD109/PLAUR/ ANXA1/SERPINB5/ SERPINB7/ ANXA8L1/COL7A1/ SFN/SERPINA4/ BIRC3/SERPINA5/ CST6/SERPINB4/	28

(Continued)

Table S1: *Continued*

	ONTOLOGY	ID	Description	Gene Ratio	Bg Ratio	*p* value	*q* value	gene ID	Count
								SERPINE1/TIMP4/SERPINA3/PI3/SERPINA1/SERPINB2/SERPINB3/A2ML1/ANGPTL4/SPOCK1/CST4/CST1/AMBP	
GO:0045861	BP	GO:0045861	negative regulation of proteolysis	27/498	350/18800	0.000138	0.000121	ANXA2/ANXA8/CD109/PLAUR/SERPINB5/SERPINB7/ANXA8L1/COL7A1/SFN/SERPINA4/IL1R2/BIRC3/SERPINA5/CST6/SERPINB4/SERPINE1/TIMP4/SERPINA3/PI3/SERPINA1/SERPINB2/SERPINB3/A2ML1/SPOCK1/CST4/CST1/AMBP	27
GO:0043062	BP	GO:0043062	extracellular structure organization	25/498	308/18800	0.000138	0.000121	MYO1E/LAMB3/MELTF/SERPINB5/COL17A1/MMP14/FSCN1/TGFBI/LOXL2/MMP11/FERMT1/MMP1/KLK7/MMP10/C6orf15/CTSV/MMP7/PRSS2/SOX9/GREM1/PRSS1/KLK5/MMP3/IBSP/COL11A1	25
GO:0019730	BP	GO:0019730	antimicrobial humoral response	15/498	122/18800	0.00014	0.000123	KRT6A/PRSS3/S100A9/KLK7/S100A7/PI3/DEFB1/	15

(Continued)

Table S1: *Continued*

	ONTOLOGY	ID	Description	Gene Ratio	Bg Ratio	*p* value	*q* value	gene ID	Count
								CXCL11/PRSS2/ DEFB4A/KLK5/ CXCL5/CXCL6/ IL36RN/PGC	
GO:0045229	BP	GO:0045229	external encapsulating structure organization	25/ 498	310/ 18800	0.000143	0.000125	MYO1E/LAMB3/ MELTF/SERPINB5/ COL17A1/MMP14/ FSCN1/TGFBI/ LOXL2/MMP11/ FERMT1/MMP1/ KLK7/MMP10/ C6orf15/CTSV/ MMP7/PRSS2/ SOX9/GREM1/ PRSS1/KLK5/ MMP3/IBSP/ COL11A1	25
GO:0001704	BP	GO:0001704	formation of primary germ layer	15/498	123/ 18800	0.000143	0.000126	ITGB4/LAMB3/ MMP14/EPHA2/ TRIM15/COL7A1/ DKK1/ITGA3/ ITGA2/LHX1/ LAMA3/HMGA2/ BMP7/HOXA11/ COL11A1	15
GO:0042303	BP	GO:0042303	molting cycle	14/498	114/ 18800	0.000282	0.000248	KRT16/CD109/ CDH3/KRT17/DKK1/ MSX2/FERMT1/ KRT14/EPS8L3/ WNT10A/FOXQ1/ SOX9/FOXE1/GAL	14
GO:0042633	BP	GO:0042633	hair cycle	14/498	114/ 18800	0.000282	0.000248	KRT16/CD109/ CDH3/KRT17/DKK1/ MSX2/FERMT1/ KRT14/EPS8L3/ WNT10A/FOXQ1/ SOX9/FOXE1/GAL	14
GO:0061436	BP	GO:0061436	establishment of skin barrier	7/498	25/ 18800	0.000392	0.000345		7

(Continued)

Table S1: *Continued*

	ONTOLOGY	ID	Description	Gene Ratio	Bg Ratio	*p* value	*q* value	gene ID	Count
								KRT16/PLEC/SFN/ALOXE3/MET/CLDN1/ABCA12	
GO:0050900	BP	GO:0050900	leukocyte migration	27/498	384/18800	0.000605	0.000532	CHST4/ANXA1/PLEC/MMP14/ITGA3/ITGA6/S100A14/ITGA2/F2RL1/S100A9/SERPINE1/IL1A/SAA1/S100A7/UMODL1/NLRP12/S100A8/CXCL11/EDN2/CALCA/GREM1/CCL7/ADORA1/CXCL5/RET/LBP/CXCL6	27
GO:0048608	BP	GO:0048608	reproductive structure development	29/498	433/18800	0.000673	0.000592	GJB3/GJB5/FOSL1/PLCD3/KRT19/GJB2/ANXA1/MDFI/SERPINB5/PPARG/WNT7A/MMP14/GREB1L/EREG/PHLDA2/LHX1/SPRR2A/IL1A/NDP/UMODL1/HOXA13/BMP7/SOX9/FGF9/PTPRN/STRA6/HOXA11/LRP2/SRD5A2	29
GO:0061458	BP	GO:0061458	reproductive system development	29/498	436/18800	0.000745	0.000655	GJB3/GJB5/FOSL1/PLCD3/KRT19/GJB2/ANXA1/MDFI/SERPINB5/PPARG/WNT7A/MMP14/GREB1L/EREG/PHLDA2/LHX1/SPRR2A/IL1A/NDP/UMODL1/HOXA13/BMP7/SOX9/FGF9/	29

(Continued)

Table S1: *Continued*

	ONTOLOGY	ID	Description	Gene Ratio	Bg Ratio	*p* value	*q* value	gene ID	Count
								PTPRN/STRA6/ HOXA11/LRP2/ SRD5A2	
GO:0061844	BP	GO:0061844	antimicrobial humoral immune response mediated by antimicrobial peptide	11/498	79/ 18800	0.000887	0.00078	KRT6A/S100A9/ KLK7/S100A7/ DEFB1/CXCL11/ DEFB4A/KLK5/ CXCL5/CXCL6/PGC	11
GO:0033561	BP	GO:0033561	regulation of water loss via skin	7/498	29/ 18800	0.000991	0.000871	KRT16/PLEC/SFN/ ALOXE3/MET/ CLDN1/ABCA12	7
GO:0030574	BP	GO:0030574	collagen catabolic process	8/498	42/ 18800	0.001428	0.001255	KLK6/MMP14/ MMP11/MMP1/ MMP10/MMP7/ PRSS2/MMP3	8
GO:0007369	BP	GO:0007369	gastrulation	17/498	190/ 18800	0.001459	0.001283	ITGB4/LAMB3/ MMP14/EPHA2/ TRIM15/IL1RN/ COL7A1/DKK1/ ITGA3/ITGA2/ LHX1/LAMA3/ HMGA2/HNF4A/ BMP7/HOXA11/ COL11A1	17
GO:0010469	BP	GO:0010469	regulation of signaling receptor activity	16/498	173/ 18800	0.001682	0.001478	PLAU/PPARG/ PSCA/DKK1/EREG/ AREG/TGFA/ PHLDA2/SERPINE1/ CBLC/NETO1/ GREM1/ADORA1/ MUC4/CACNG4/ PCSK9	16
GO:0022600	BP	GO:0022600	digestive system process	12/498	104/ 18800	0.00213	0.001872	INAVA/KCNN4/ NPSR1/SERPINA3/ VSIG1/SOX9/ NEUROD1/TFF1/ TFF2/MUC2/MUC4/ MUC13	12
GO:0048333	BP	GO:0048333		7/498		0.002157	0.001896		7

(Continued)

Table S1: *Continued*

	ONTOLOGY	ID	Description	Gene Ratio	Bg Ratio	*p* value	*q* value	gene ID	Count
			mesodermal cell differentiation		33/ 18800			ITGB4/TRIM15/ DKK1/ITGA3/ ITGA2/HMGA2/ HOXA11	
GO:0001942	BP	GO:0001942	hair follicle development	11/498	89/ 18800	0.002332	0.00205	CD109/CDH3/ KRT17/DKK1/MSX2/ FERMT1/WNT10A/ FOXQ1/SOX9/ FOXE1/GAL	11
GO:0022404	BP	GO:0022404	molting cycle process	11/498	92/ 18800	0.003044	0.002675	CD109/CDH3/ KRT17/DKK1/MSX2/ FERMT1/WNT10A/ FOXQ1/SOX9/ FOXE1/GAL	11
GO:0022405	BP	GO:0022405	hair cycle process	11/498	92/ 18800	0.003044	0.002675	CD109/CDH3/ KRT17/DKK1/MSX2/ FERMT1/WNT10A/ FOXQ1/SOX9/ FOXE1/GAL	11
GO:0098773	BP	GO:0098773	skin epidermis development	11/498	93/ 18800	0.003293	0.002894	CD109/CDH3/ KRT17/DKK1/MSX2/ FERMT1/WNT10A/ FOXQ1/SOX9/ FOXE1/GAL	11
GO:0018149	BP	GO:0018149	peptide cross-linking	7/498	36/ 18800	0.003488	0.003065	ANXA1/SPRR1B/ TGM5/SPRR1A/ SPRR3/PI3/IVL	7
GO:0097529	BP	GO:0097529	myeloid leukocyte migration	18/ 498	229/ 18800	0.003767	0.003311	ANXA1/MMP14/ S100A14/S100A9/ SERPINE1/IL1A/ SAA1/S100A7/ UMODL1/S100A8/ CXCL11/EDN2/ CALCA/GREM1/ CCL7/CXCL5/LBP/ CXCL6	18
GO:0031638	BP	GO:0031638	zymogen activation	9/498	64/ 18800	0.004	0.003515	ANXA2/PERP/ MELTF/PLAU/ S100A10/MMP14/	9

(Continued)

Table S1: *Continued*

	ONTOLOGY	ID	Description	Gene Ratio	Bg Ratio	*p* value	*q* value	gene ID	Count
GO:0097530	BP	GO:0097530	granulocyte migration	14/498	154/ 18800	0.005398	0.004743	ASPH/PRSS3/ SERPINE1 ANXA1/S100A14/ S100A9/IL1A/SAA1/ S100A7/UMODL1/ S100A8/CXCL11/ EDN2/CCL7/CXCL5/ LBP/CXCL6	14
GO:0042634	BP	GO:0042634	regulation of hair cycle	6/498	28/ 18800	0.006283	0.005521	CDH3/KRT17/ MSX2/FERMT1/ EPS8L3/GAL	6
GO:0048736	BP	GO:0048736	appendage development	15/498	177/ 18800	0.006283	0.005521	ITGB4/WNT7A/ PITX1/DKK1/ITGA6/ MSX2/ASPH/ TFAP2A/HOXA13/ BMP7/SOX9/ GREM1/FGF9/ FREM2/HOXA11	15
GO:0060173	BP	GO:0060173	limb development	15/498	177/ 18800	0.006283	0.005521	ITGB4/WNT7A/ PITX1/DKK1/ITGA6/ MSX2/ASPH/ TFAP2A/HOXA13/ BMP7/SOX9/ GREM1/FGF9/ FREM2/HOXA11	15
GO:0001667	BP	GO:0001667	ameboidal-type cell migration	28/ 498	480/ 18800	0.006988	0.006141	KRT16/ITGB4/ SEMA7A/ANXA1/ FGFBP1/PPARG/ PLEC/SRPX2/ WNT7A/EPHA2/ SEMA3B/ITGA3/ ITGA2/LOXL2/ FERMT1/ANXA3/ PRSS3/S100A2/ MET/PTPRR/FAT2/ BMP7/EDN2/SOX9/ GREM1/S100P/RET/ FGF19	28
GO:0038127	BP	GO:0038127	ERBB signaling pathway	12/498		0.006988	0.006141		12

(Continued)

Table S1: *Continued*

	ONTOLOGY	ID	Description	Gene Ratio	Bg Ratio	*p* value	*q* value	gene ID	Count
					121/ 18800			PLAUR/FAM83B/ SH3TC2/EREG/ FAM83A/AREG/ TGFA/CBLC/PTK6/ PTPRR/SOX9/ ADORA1	
GO:0033627	BP	GO:0033627	cell adhesion mediated by integrin	10/ 498	86/ 18800	0.006988	0.006141	ITGB4/PLAU/ EPHA2/ITGA3/ ITGA6/ITGA2/ FERMT1/SERPINE1/ FYB2/RET	10
GO:0001706	BP	GO:0001706	endoderm formation	8/498	55/ 18800	0.007061	0.006205	LAMB3/MMP14/ COL7A1/DKK1/ LHX1/LAMA3/ HMGA2/COL11A1	8
GO:0035107	BP	GO:0035107	appendage morphogenesis	13/498	142/ 18800	0.007607	0.006685	WNT7A/PITX1/ DKK1/MSX2/ASPH/ TFAP2A/HOXA13/ BMP7/SOX9/ GREM1/FGF9/ FREM2/HOXA11	13
GO:0035108	BP	GO:0035108	limb morphogenesis	13/498	142/ 18800	0.007607	0.006685	WNT7A/PITX1/ DKK1/MSX2/ASPH/ TFAP2A/HOXA13/ BMP7/SOX9/ GREM1/FGF9/ FREM2/HOXA11	13
GO:0002064	BP	GO:0002064	epithelial cell development	16/498	203/ 18800	0.007607	0.006685	MYO1E/PLEC/ WNT7A/EPHA2/ SFN/F2RL1/MET/ IL1A/CLDN1/ POF1B/ABCA12/ VSIG1/HOXA13/ HNF4A/SOX9/ DACT2	16
GO:0051797	BP	GO:0051797	regulation of hair follicle development	5/498	19/ 18800	0.007689	0.006757	CDH3/KRT17/ MSX2/FERMT1/GAL	5
GO:0001707	BP	GO:0001707	mesoderm formation	9/498	73/ 18800	0.008907	0.007827	ITGB4/EPHA2/ TRIM15/DKK1/	9

(Continued)

Table S1: *Continued*

	ONTOLOGY	ID	Description	Gene Ratio	Bg Ratio	*p* value	*q* value	gene ID	Count
								ITGA3/ITGA2/HMGA2/BMP7/HOXA11	
GO:0010817	BP	GO:0010817	regulation of hormone levels	28/498	496/18800	0.01021	0.008973	SLCO4A1/KLK6/ANXA1/PPARG/IL1RN/ANO1/DHRS9/AKR1B15/CYP2S1/IL11/ABCA12/ABCC8/HNF4A/CYP2C9/NEUROD1/ADORA1/UGT2B7/FOXE1/AKR1B10/GAL/DUOXA2/PTPRN/NR0B2/DUOX2/CRYM/CYP2C18/SRD5A2/PCSK2	28
GO:0071621	BP	GO:0071621	granulocyte chemotaxis	12/498	128/18800	0.01021	0.008973	ANXA1/S100A14/S100A9/SAA1/S100A7/S100A8/CXCL11/EDN2/CCL7/CXCL5/LBP/CXCL6	12
GO:0050891	BP	GO:0050891	multicellular organismal water homeostasis	8/498	59/18800	0.01021	0.008973	KRT16/PLEC/SFN/ALOXE3/MET/CLDN1/ABCA12/CYP4F12	8
GO:0048332	BP	GO:0048332	mesoderm morphogenesis	9/498	75/18800	0.010299	0.009051	ITGB4/EPHA2/TRIM15/DKK1/ITGA3/ITGA2/HMGA2/BMP7/HOXA11	9
GO:0002062	BP	GO:0002062	chondrocyte differentiation	11/498	110/18800	0.010339	0.009086	WNT7A/TGFBI/MSX2/LOXL2/HMGA2/RFLNA/SOX9/GREM1/FGF9/HOXA11/COL11A1	11

(Continued)

Table S1: *Continued*

	ONTOLOGY	ID	Description	Gene Ratio	Bg Ratio	*p* value	*q* value	gene ID	Count
GO:0060065	BP	GO:0060065	uterus development	5/498	21/18800	0.011491	0.010098	WNT7A/GREB1L/LHX1/STRA6/HOXA11	5
GO:0035987	BP	GO:0035987	endodermal cell differentiation	7/498	46/18800	0.011865	0.010427	LAMB3/MMP14/COL7A1/DKK1/LAMA3/HMGA2/COL11A1	7
GO:0030595	BP	GO:0030595	leukocyte chemotaxis	17/498	236/18800	0.011881	0.010441	ANXA1/PLEC/S100A14/F2RL1/S100A9/SERPINE1/SAA1/S100A7/S100A8/CXCL11/EDN2/CALCA/GREM1/CCL7/CXCL5/LBP/CXCL6	17
GO:0044848	BP	GO:0044848	biological phase	4/498	12/18800	0.012181	0.010705	CDH3/MSX2/FERMT1/GAL	4
GO:0002526	BP	GO:0002526	acute inflammatory response	11/498	113/18800	0.012181	0.010705	IL20RB/VNN1/IL1A/SERPINA3/SAA2/SAA1/SERPINA1/S100A8/ADORA1/LBP/IL31RA	11
GO:0043616	BP	GO:0043616	keratinocyte proliferation	7/498	47/18800	0.012848	0.011291	CD109/CDH3/EREG/SFN/FERMT1/AREG/KLK8	7
GO:0050878	BP	GO:0050878	regulation of body fluid levels	23/498	382/18800	0.013526	0.011887	KRT16/ANXA8/PLAUR/PLAU/PLEC/KCNN4/SFN/ITGA2/XDH/F2RL1/ALOXE3/MET/SERPINE1/CLDN1/SAA1/SERPINA1/SERPINB2/ABCA12/F5/HNF4A/ADORA1/CYP4F12/CYP4F11	23
GO:0042363	BP	GO:0042363	fat-soluble vitamin catabolic process	4/498	13/18800	0.015647	0.01375	CYP24A1/CYP4F12/CYP4F3/CYP4F11	4

(Continued)

Table S1: *Continued*

	ONTOLOGY	ID	Description	Gene Ratio	Bg Ratio	*p* value	*q* value	gene ID	Count
GO:0045741	BP	GO:0045741	positive regulation of epidermal growth factor-activated receptor activity	4/498	13/ 18800	0.015647	0.01375	EREG/AREG/TGFA/ ADORA1	4
GO:1900426	BP	GO:1900426	positive regulation of defense response to bacterium	4/498	13/ 18800	0.015647	0.01375	F2RL1/KLK7/ KLK5/PGC	4
GO:2001053	BP	GO:2001053	regulation of mesenchymal cell apoptotic process	4/498	13/ 18800	0.015647	0.01375	MSX2/HOXA13/ BMP7/SOX9	4
GO:0060326	BP	GO:0060326	cell chemotaxis	20/ 498	315/ 18800	0.016072	0.014124	ANXA1/PLEC/ EPHA2/S100A14/ F2RL1/MET/ S100A9/SERPINE1/ SAA1/S100A7/ S100A8/CXCL11/ EDN2/CALCA/ GREM1/CCL7/ DEFB4A/CXCL5/ LBP/CXCL6	20
GO:0042742	BP	GO:0042742	defense response to bacterium	22/ 498	364/ 18800	0.016072	0.014124	KRT6A/OAS1/ EPHA2/S100A14/ ANXA3/F2RL1/ S100A9/GBP6/ SERPINE1/KLK7/ IL22RA1/S100A7/ PI3/S100A8/DEFB1/ LCN2/IFNE/ DEFB4A/KLK5/LBP/ CXCL6/PGC	22
GO:0015837	BP	GO:0015837	amine transport	10/ 498	100/ 18800	0.016532	0.014528	SYT8/SNCG/NTSR1/ TH/SYT12/ADORA1/ RHCG/SYT13/SYT4/ RAB3B	10
GO:0030326	BP	GO:0030326	embryonic limb morphogenesis	11/498	119/ 18800	0.016532	0.014528	WNT7A/PITX1/ DKK1/MSX2/ TFAP2A/HOXA13/ BMP7/GREM1/ FGF9/FREM2/ HOXA11	11

(Continued)

Table S1: *Continued*

	ONTOLOGY	ID	Description	Gene Ratio	Bg Ratio	*p* value	*q* value	gene ID	Count
GO:0035113	BP	GO:0035113	embryonic appendage morphogenesis	11/498	119/ 18800	0.016532	0.014528	WNT7A/PITX1/ DKK1/MSX2/ TFAP2A/HOXA13/ BMP7/GREM1/ FGF9/FREM2/ HOXA11	11
GO:0045742	BP	GO:0045742	positive regulation of epidermal growth factor receptor signaling pathway	6/498	36/ 18800	0.01674	0.014711	PLAUR/EREG/ AREG/TGFA/PTK6/ ADORA1	6
GO:0030104	BP	GO:0030104	water homeostasis	8/498	66/ 18800	0.017012	0.01495	KRT16/PLEC/SFN/ ALOXE3/MET/ CLDN1/ABCA12/ CYP4F12	8
GO:0032963	BP	GO:0032963	collagen metabolic process	10/ 498	101/ 18800	0.017015	0.014952	KLK6/MMP14/ SERPINB7/ITGA2/ MMP11/MMP1/ MMP10/MMP7/ PRSS2/MMP3	10
GO:0034329	BP	GO:0034329	cell junction assembly	24/ 498	420/ 18800	0.018159	0.015958	ITGB4/GJB2/PKP2/ COL17A1/S100A10/ PLEC/SRPX2/ WNT7A/MMP14/ EPHA2/FSCN1/ DKK1/ITGA6/ ITGA2/CLDN1/ POF1B/DNER/ CTNND2/GREM1/ GJB6/SLITRK6/ ADGRF1/ GABRB3/PKP1	24
GO:0045216	BP	GO:0045216	cell-cell junction organization	15/498	205/ 18800	0.01857	0.016319	GJB2/PERP/DSG2/ PKP2/PLEC/INAVA/ EPHA2/FSCN1/ F2RL1/CLDN1/ POF1B/ABCC8/ CTNND2/ GJB6/PKP1	15
GO:0042182	BP	GO:0042182	ketone catabolic process	4/498		0.018705	0.016438		4

(Continued)

Table S1: *Continued*

	ONTOLOGY	ID	Description	Gene Ratio	Bg Ratio	*p* value	*q* value	gene ID	Count
					14/ 18800			KYNU/CYP4F12/ CYP4F3/CYP4F11	
GO:0031639	BP	GO:0031639	plasminogen activation	5/498	25/ 18800	0.020306	0.017845	ANXA2/MELTF/ PLAU/S100A10/ SERPINE1	5
GO:0062149	BP	GO:0062149	detection of stimulus involved in sensory perception of pain	5/498	25/ 18800	0.020306	0.017845	ITGA2/ANO1/ NTSR1/CALCA/ ADORA1	5
GO:1901186	BP	GO:1901186	positive regulation of ERBB signaling pathway	6/498	38/ 18800	0.020649	0.018146	PLAUR/EREG/ AREG/TGFA/PTK6/ ADORA1	6
GO:0010038	BP	GO:0010038	response to metal ion	21/498	351/ 18800	0.021391	0.018798	S100A16/LOXL2/ SYT8/MT2A/ASCL1/ TFAP2A/IL1A/ CLDN1/SLC13A5/ ADGRV1/MT1A/ GUCA1A/S100A8/ ABCC8/TH/SYT12/ TFF1/SYT13/SYT4/ CNGA3/AOC1	21
GO:0009111	BP	GO:0009111	vitamin catabolic process	4/498	15/ 18800	0.02285	0.02008	CYP24A1/CYP4F12/ CYP4F3/CYP4F11	4
GO:0010755	BP	GO:0010755	regulation of plasminogen activation	4/498	15/ 18800	0.02285	0.02008	ANXA2/MELTF/ S100A10/SERPINE1	4
GO:0048820	BP	GO:0048820	hair follicle maturation	4/498	15/ 18800	0.02285	0.02008	CDH3/MSX2/ FERMT1/GAL	4
GO:0097152	BP	GO:0097152	mesenchymal cell apoptotic process	4/498	15/ 18800	0.02285	0.02008	MSX2/HOXA13/ BMP7/SOX9	4
GO:0007173	BP	GO:0007173	epidermal growth factor receptor signaling pathway	10/ 498	108/ 18800	0.025424	0.022343	PLAUR/FAM83B/ EREG/FAM83A/ AREG/TGFA/CBLC/ PTK6/SOX9/ ADORA1	10
GO:1990266	BP	GO:1990266	neutrophil migration	11/498	128/ 18800	0.025653	0.022544	S100A9/IL1A/SAA1/ UMODL1/S100A8/ CXCL11/EDN2/ CCL7/CXCL5/LBP/ CXCL6	11
GO:0010631	BP	GO:0010631	epithelial cell migration	21/498		0.025653	0.022544		21

(Continued)

Table S1: *Continued*

	ONTOLOGY	ID	Description	Gene Ratio	Bg Ratio	*p* value	*q* value	gene ID	Count
					358/ 18800			KRT16/ANXA1/ FGFBP1/PPARG/ SRPX2/WNT7A/ EPHA2/ITGA3/ ITGA2/LOXL2/ FERMT1/ANXA3/ PRSS3/S100A2/ MET/PTPRR/FAT2/ EDN2/SOX9/ GREM1/S100P	
GO:0016540	BP	GO:0016540	protein autoprocessing	5/498	27/ 18800	0.026523	0.023308	KLK6/MYRF/CTSE/ PCSK9/PCSK2	5
GO:0090132	BP	GO:0090132	epithelium migration	21/498	361/ 18800	0.027764	0.024399	KRT16/ANXA1/ FGFBP1/PPARG/ SRPX2/WNT7A/ EPHA2/ITGA3/ ITGA2/LOXL2/ FERMT1/ANXA3/ PRSS3/S100A2/ MET/PTPRR/FAT2/ EDN2/SOX9/ GREM1/S100P	21
GO:0050965	BP	GO:0050965	detection of temperature stimulus involved in sensory perception of pain	4/498	16/ 18800	0.027764	0.024399	ANO1/NTSR1/ CALCA/ADORA1	4
GO:0051238	BP	GO:0051238	sequestering of metal ion	4/498	16/ 18800	0.027764	0.024399	S100A9/S100A7/ S100A8/LCN2	4
GO:0051216	BP	GO:0051216	cartilage development	14/498	195/ 18800	0.028901	0.025397	WNT7A/PITX1/ TGFBI/MSX2/ LOXL2/HMGA2/ RFLNA/BMP7/ SOX9/GREM1/ BARX2/FGF9/ HOXA11/COL11A1	14
GO:0051091	BP	GO:0051091	positive regulation of DNA-binding transcription factor activity	17/498	265/ 18800	0.02939	0.025827	FOSL1/TRIM31/ PPARG/TRIM15/ SPHK1/ANXA3/ CARD11/S100A9/ OPRD1/NDP/	17

(Continued)

Table S1: *Continued*

	ONTOLOGY	ID	Description	Gene Ratio	Bg Ratio	*p* value	*q* value	gene ID	Count
								NTSR1/AIM2/S100A8/NEUROD1/GREM1/ZIC2/ADGRF1	
GO:0031589	BP	GO:0031589	cell-substrate adhesion	21/498	364/18800	0.029614	0.026024	ITGB4/LAMB3/MELTF/LYPD3/PLAU/COL17A1/S100A10/MMP14/UNC13D/ITGA3/ITGA6/ITGA2/FERMT1/SERPINE1/L1CAM/FAT2/GREM1/SPOCK1/OLFM4/MUC4/MSLN	21
GO:0007498	BP	GO:0007498	mesoderm development	11/498	132/18800	0.030273	0.026604	ITGB4/EPHA2/TRIM15/DKK1/ITGA3/ITGA2/LHX1/HMGA2/BMP7/HOXA11/DLL3	11
GO:0048754	BP	GO:0048754	branching morphogenesis of an epithelial tube	12/498	153/18800	0.030273	0.026604	MMP14/EPHA2/GREB1L/MSX2/AREG/LHX1/MET/BMP7/SOX9/GREM1/NKX2-1/HOXA11	12
GO:0016485	BP	GO:0016485	protein processing	16/498	243/18800	0.030273	0.026604	ANXA2/KLK6/PERP/MELTF/PLAU/S100A10/MMP14/IL1R2/ASPH/PRSS3/SERPINE1/MYRF/TMPRSS4/CTSE/PCSK9/PCSK2	16
GO:0042063	BP	GO:0042063	gliogenesis	18/498	291/18800	0.030273	0.026604	ITGB4/ANXA1/PLEC/MMP14/SH3TC2/CLCF1/AREG/ASCL1/S100A9/DNER/	18

(Continued)

Table S1: *Continued*

	ONTOLOGY	ID	Description	Gene Ratio	Bg Ratio	*p* value	*q* value	gene ID	Count
GO:0090130	BP	GO:0090130	tissue migration	21/498	366/18800	0.030296	0.026624	ZNF488/S100A8/MYRF/ABCC8/SOX9/FGF5/CDK5R2/LRP2 KRT16/ANXA1/FGFBP1/PPARG/SRPX2/WNT7A/EPHA2/ITGA3/ITGA2/LOXL2/FERMT1/ANXA3/PRSS3/S100A2/MET/PTPRR/FAT2/EDN2/SOX9/GREM1/S100P	21
GO:0019369	BP	GO:0019369	arachidonic acid metabolic process	7/498	58/18800	0.030436	0.026747	ALOXE3/CYP2S1/CYP2C9/CYP4F12/CYP4F3/CYP2C18/CYP4F11	7
GO:0061041	BP	GO:0061041	regulation of wound healing	11/498	133/18800	0.030643	0.026928	CD109/ANXA1/PLAU/FERMT1/TNFRSF12A/F2RL1/SERPINE1/CLDN1/SERPINB2/ABCC8/DUOX2	11
GO:0050953	BP	GO:0050953	sensory perception of light stimulus	15/498	221/18800	0.030808	0.027073	CDH3/TGFBI/NXNL2/NDP/ADGRV1/GUCA1A/GRK1/REEP6/TH/ZIC2/MYO3B/CNGA3/SLITRK6/COL11A1/USH1C	15
GO:0002544	BP	GO:0002544	chronic inflammatory response	4/498	17/18800	0.031497	0.027679	UNC13D/VNN1/S100A9/S100A8	4
GO:0060192	BP	GO:0060192	negative regulation of lipase activity	4/498	17/18800	0.031497	0.027679	ANXA8/ANXA1/ANXA8L1/ANGPTL4	4
GO:0050954	BP	GO:0050954	sensory perception of mechanical stimulus	13/498	177/18800	0.031706	0.027863	GJB2/ITGA2/TFAP2A/LHFPL3/ADGRV1/TH/GJB6/MYO3B/CRYM/	13

(Continued)

Table S1: *Continued*

	ONTOLOGY	ID	Description	Gene Ratio	Bg Ratio	*p* value	*q* value	gene ID	Count
GO:0048562	BP	GO:0048562	embryonic organ morphogenesis	18/498	294/18800	0.031706	0.027863	LRP2/SLITRK6/COL11A1/USH1C MDFI/MMP14/EPHA2/LHX1/TFAP2A/TH/BMP7/SOX9/NEUROD1/GJB6/FOXE1/FGF9/STRA6/MYO3B/HOXA11/SLITRK6/COL11A1/USH1C	18
GO:0007605	BP	GO:0007605	sensory perception of sound	12/498	156/18800	0.032632	0.028676	GJB2/TFAP2A/LHFPL3/ADGRV1/TH/GJB6/MYO3B/CRYM/LRP2/SLITRK6/COL11A1/USH1C	12
GO:0033559	BP	GO:0033559	unsaturated fatty acid metabolic process	10/498	115/18800	0.033445	0.029391	ANXA1/ELOVL6/ALOXE3/CYP2S1/CYP2C9/EDN2/CYP4F12/CYP4F3/CYP2C18/CYP4F11	10
GO:0030856	BP	GO:0030856	regulation of epithelial cell differentiation	12/498	157/18800	0.033925	0.029813	CD109/SFN/SPRED3/MSX2/XDH/ASCL1/LHX1/SULT2B1/SERPINE1/IL1A/ABCA12/SOX9	12
GO:0007176	BP	GO:0007176	regulation of epidermal growth factor-activated receptor activity	5/498	30/18800	0.035706	0.031378	EREG/AREG/TGFA/CBLC/ADORA1	5
GO:0007492	BP	GO:0007492	endoderm development	8/498	78/18800	0.035706	0.031378	LAMB3/MMP14/COL7A1/DKK1/LHX1/LAMA3/HMGA2/COL11A1	8
GO:0030540	BP	GO:0030540	female genitalia development	4/498	18/18800	0.037055	0.032563	LHX1/STRA6/LRP2/SRD5A2	4
GO:0031644	BP	GO:0031644	regulation of nervous system process	11/498	138/18800	0.037402	0.032868	WNT7A/ITGA2/IL1A/OPRD1/NTSR1/KLK8/	11

(Continued)

Table S1: *Continued*

	ONTOLOGY	ID	Description	Gene Ratio	Bg Ratio	*p* value	*q* value	gene ID	Count
								ZNF488/MYRF/NETO1/ADORA1/CACNG4	
GO:2000273	BP	GO:2000273	positive regulation of signaling receptor activity	6/498	45/18800	0.037402	0.032868	EREG/AREG/TGFA/GREM1/ADORA1/CACNG4	6
GO:1901184	BP	GO:1901184	regulation of ERBB signaling pathway	8/498	79/18800	0.037585	0.033029	PLAUR/SH3TC2/EREG/AREG/TGFA/CBLC/PTK6/ADORA1	8
GO:0035115	BP	GO:0035115	embryonic forelimb morphogenesis	5/498	31/18800	0.039751	0.034933	WNT7A/MSX2/TFAP2A/HOXA13/HOXA11	5
GO:0048732	BP	GO:0048732	gland development	23/498	431/18800	0.039751	0.034933	ANXA1/SERPINB5/PITX1/EPHA2/MSX2/ITGA2/XDH/AREG/TGFA/ASCL1/MET/CLDN1/WNT10A/HOXA13/HNF4A/BMP7/SOX9/NKX2-1/FOXE1/STRA6/HOXA11/PCSK9/FGL1	23
GO:0001895	BP	GO:0001895	retina homeostasis	8/498	80/18800	0.039857	0.035026	SLC2A1/CDH3/NXNL2/ADGRV1/ZG16B/PIP/CST4/USH1C	8
GO:0042445	BP	GO:0042445	hormone metabolic process	15/498	230/18800	0.040003	0.035154	KLK6/DHRS9/AKR1B15/CYP2S1/CYP2C9/UGT2B7/FOXE1/AKR1B10/GAL/DUOXA2/DUOX2/CRYM/CYP2C18/SRD5A2/PCSK2	15
GO:0048806	BP	GO:0048806	genitalia development	6/498	46/18800	0.040123	0.03526	GREB1L/LHX1/HOXA13/STRA6/LRP2/SRD5A2	6

(Continued)

Table S1: *Continued*

	ONTOLOGY	ID	Description	Gene Ratio	Bg Ratio	*p* value	*q* value	gene ID	Count
GO:0019221	BP	GO:0019221	cytokine-mediated signaling pathway	25/498	486/18800	0.040244	0.035365	IL20RB/PPARG/OAS1/IL1RN/OASL/EREG/SPHK1/CLCF1/IL1R2/BIRC3/F2RL1/IL1A/IL22RA1/AIM2/IFNE/CXCL11/CRLF2/EDN2/CCL7/TFF2/CXCL5/DUOX2/CXCL6/IL31RA/IL36RN	25
GO:0061138	BP	GO:0061138	morphogenesis of a branching epithelium	13/498	185/18800	0.041542	0.036506	MMP14/EPHA2/GREB1L/MSX2/AREG/LHX1/MET/HOXA13/BMP7/SOX9/GREM1/NKX2-1/HOXA11	13
GO:0019373	BP	GO:0019373	epoxygenase P450 pathway	4/498	19/18800	0.041845	0.036773	CYP2S1/CYP2C9/CYP4F12/CYP2C18	4
GO:0050961	BP	GO:0050961	detection of temperature stimulus involved in sensory perception	4/498	19/18800	0.041845	0.036773	ANO1/NTSR1/CALCA/ADORA1	4
GO:0007548	BP	GO:0007548	sex differentiation	17/498	281/18800	0.042588	0.037426	WNT7A/MMP14/GREB1L/EREG/LHX1/SPRR2A/IL1A/UMODL1/HOXA13/HNF4A/SOX9/FGF9/PTPRN/STRA6/HOXA11/LRP2/SRD5A2	17
GO:0006690	BP	GO:0006690	icosanoid metabolic process	10/498	121/18800	0.042809	0.037619	ANXA1/ALOXE3/CYP2S1/CYP2C9/EDN2/CYP4F12/CYP4F3/GGTLC1/CYP2C18/CYP4F11	10
GO:0007411	BP	GO:0007411	axon guidance	15/498	234/18800	0.044556	0.039155	LAMC2/SEMA7A/EPHA2/SEMA3B/LHX1/LAMA3/TUBB3/GFRA3/	15

(Continued)

Table S1: *Continued*

	ONTOLOGY	ID	Description	Gene Ratio	Bg Ratio	*p* value	*q* value	gene ID	Count
								EFNA2/L1CAM/ EVX1/EPHA10/ BMP7/RET/CDK5R2	
GO:0009410	BP	GO:0009410	response to xenobiotic stimulus	22/ 498	411/ 18800	0.044556	0.039155	FOSL1/CDH3/ SMOX/ITGA3/ ITGA2/TGFA/ TIMP4/CYP2S1/ AIM2/ABCC8/TH/ HNF4A/CYP2C9/ NEUROD1/CA9/ CYP4F12/GAL/RET/ CYP2C18/SRD5A2/ UGT1A6/SST	22
GO:0007160	BP	GO:0007160	cell-matrix adhesion	15/498	235/ 18800	0.04561	0.040081	ITGB4/LYPD3/ PLAU/COL17A1/ S100A10/MMP14/ ITGA3/ITGA6/ ITGA2/FERMT1/ SERPINE1/L1CAM/ GREM1/ MUC4/MSLN	15
GO:0097485	BP	GO:0097485	neuron projection guidance	15/498	235/ 18800	0.04561	0.040081	LAMC2/SEMA7A/ EPHA2/SEMA3B/ LHX1/LAMA3/ TUBB3/GFRA3/ EFNA2/L1CAM/ EVX1/EPHA10/ BMP7/RET/CDK5R2	15
GO:1903034	BP	GO:1903034	regulation of response to wounding	12/498	166/ 18800	0.046029	0.040449	CD109/ANXA1/ PLAU/FERMT1/ TNFRSF12A/F2RL1/ SERPINE1/CLDN1/ KLK8/SERPINB2/ ABCC8/DUOX2	12
GO:0006953	BP	GO:0006953	acute-phase response	6/498	48/ 18800	0.046029	0.040449	IL1A/SERPINA3/ SAA2/SAA1/ SERPINA1/LBP	6
GO:0061448	BP	GO:0061448	connective tissue development	16/498	260/ 18800	0.046149	0.040555	WNT7A/PITX1/ SERPINB7/TGFBI/	16

(Continued)

Table S1: *Continued*

	ONTOLOGY	ID	Description	Gene Ratio	Bg Ratio	*p* value	*q* value	gene ID	Count
								MSX2/LOXL2/HMGA2/UMODL1/RFLNA/BMP7/SOX9/GREM1/BARX2/FGF9/HOXA11/COL11A1	
GO:0003382	BP	GO:0003382	epithelial cell morphogenesis	5/498	33/18800	0.046572	0.040927	MET/POF1B/VSIG1/HOXA13/DACT2	5
GO:0001502	BP	GO:0001502	cartilage condensation	4/498	20/18800	0.047011	0.041313	WNT7A/SOX9/BARX2/COL11A1	4
GO:1900424	BP	GO:1900424	regulation of defense response to bacterium	4/498	20/18800	0.047011	0.041313	F2RL1/KLK7/KLK5/PGC	4
GO:0030850	BP	GO:0030850	prostate gland development	6/498	49/18800	0.049331	0.043351	ANXA1/SERPINB5/HOXA13/BMP7/SOX9/HOXA11	6
GO:0071248	BP	GO:0071248	cellular response to metal ion	13/498	191/18800	0.049331	0.043351	SYT8/MT2A/TFAP2A/CLDN1/SLC13A5/ADGRV1/MT1A/GUCA1A/TH/SYT12/SYT13/SYT4/AOC1	13
GO:0001533	CC	GO:0001533	cornified envelope	16/527	45/19594	6.98×10^{-12}	6.03×10^{-12}	ANXA1/DSG2/PKP2/SPRR1B/SPRR1A/SPRR3/SPRR2D/CST6/SPRR2A/SPRR2F/DSG3/PI3/SCEL/IVL/DSC3/PKP1	16
GO:0062023	CC	GO:0062023	collagen-containing extracellular matrix	38/527	429/19594	2.43×10^{-8}	2.1×10^{-8}	LAMC2/ITGB4/ANXA2/ANXA8/LAMB3/SEMA7A/ANXA1/COL17A1/S100A10/S100A6/SRPX2/SEMA3B/LAD1/COL7A1/TGFBI/LOXL2/SERPINA5/LAMA3/FIBCD1/S100A9/SERPINE1/NDP/	38

(Continued)

Table S1: *Continued*

	ONTOLOGY	ID	Description	Gene Ratio	Bg Ratio	*p* value	*q* value	gene ID	Count
								SERPINA3/L1CAM/S100A7/SERPINA1/S100A8/BMP7/ANGPTL4/GREM1/PRSS1/MUC2/FREM2/TINAG/COL25A1/AMBP/COL11A1/FGL1	
GO:0031225	CC	GO:0031225	anchored component of membrane	20/527	169/19594	3.02×10^{-6}	2.61×10^{-6}	CD109/RAET1L/LY6D/PLAUR/SEMA7A/MELTF/LYPD3/PSCA/LYPD5/RAB27B/VNN1/ULBP2/GFRA3/EFNA2/NT5E/ALPP/LY6K/PRMT8/RAB3B/MSLN	20
GO:0045111	CC	GO:0045111	intermediate filament cytoskeleton	25/527	257/19594	3.02×10^{-6}	2.61×10^{-6}	KRT16/KRT6A/KRT6B/KRT6C/KRT19/PKP2/KRT17/PLEC/MMP14/KRT8/KRT7/KRT78/KRT80/KRT14/KRT81/KRT23/S100A8/KRT13/KRT15/HOXA13/KRT5/CASP14/KRT75/KRT20/PKP1	25
GO:0005882	CC	GO:0005882	intermediate filament	22/527	216/19594	7.19×10^{-6}	6.22×10^{-6}	KRT16/KRT6A/KRT6B/KRT6C/KRT19/PKP2/KRT17/PLEC/KRT8/KRT7/KRT78/KRT80/KRT14/KRT81/KRT23/KRT13/KRT15/	22

(Continued)

Table S1: *Continued*

	ONTOLOGY	ID	Description	Gene Ratio	Bg Ratio	*p* value	*q* value	gene ID	Count
GO:0045095	CC	GO:0045095	keratin filament	14/527	102/ 19594	3.66×10^{-5}	3.16×10^{-5}	KRT5/CASP14/ KRT75/KRT20/PKP1 KRT6A/KRT6B/ KRT6C/KRT17/ KRT8/KRT7/KRT78/ KRT80/KRT14/ KRT81/KRT13/ KRT5/CASP14/ KRT75	14
GO:0030057	CC	GO:0030057	desmosome	7/527	25/ 19594	0.000163	0.000141	PERP/DSG2/PKP2/ POF1B/DSG3/ DSC3/PKP1	7
GO:0005604	CC	GO:0005604	basement membrane	12/527	95/ 19594	0.000421	0.000364	LAMC2/ITGB4/ ANXA2/LAMB3/ COL17A1/LAD1/ COL7A1/TGFBI/ LOXL2/LAMA3/ FREM2/TINAG	12
GO:0045178	CC	GO:0045178	basal part of cell	21/527	269/ 19594	0.000561	0.000485	ITGB4/ANXA2/ SLC2A1/SLC16A3/ ANXA1/ITGA3/ ITGA2/PROM2/ TGFA/KRT14/MET/ CLDN1/CALHM3/ SLC4A11/VSIG1/ CA9/ADORA1/ CLCA2/RHCG/ SLCO1B3/CDH17	21
GO:0009925	CC	GO:0009925	basal plasma membrane	19/527	251/ 19594	0.001959	0.001694	ITGB4/ANXA2/ SLC2A1/SLC16A3/ ANXA1/ITGA3/ PROM2/TGFA/ MET/CLDN1/ CALHM3/SLC4A11/ VSIG1/CA9/ ADORA1/CLCA2/ RHCG/SLCO1B3/ CDH17	19
GO:0005911	CC	GO:0005911	cell-cell junction	29/527		0.002916	0.002522		29

(Continued)

Table S1: *Continued*

	ONTOLOGY	ID	Description	Gene Ratio	Bg Ratio	*p* value	*q* value	gene ID	Count
					497/ 19594			GJB3/GJB4/GJB5/ TRIM29/ANXA2/ SLC2A1/MYO1E/ GJB2/PERP/ANXA1/ DSG2/CDH3/PKP2/ COL17A1/EPHA2/ STEAP1/FSCN1/ PDLIM4/CLDN1/ POF1B/CALB2/ DSG3/CTNND2/ FAT2/DSC3/GJB6/ ABCC2/PKP1/AOC1	
GO:0005796	CC	GO:0005796	Golgi lumen	11/527	104/ 19594	0.003457	0.00299	MUC16/WNT7A/ MMP14/MMP11/ DEFB1/MUC5AC/ DEFB4A/MUC2/ MUC4/MUC5B/ MUC13	11
GO:1904090	CC	GO:1904090	peptidase inhibitor complex	4/527	11/ 19594	0.004181	0.003616	PLAU/SERPINA5/ SERPINE1/KLK8	4
GO:0005921	CC	GO:0005921	gap junction	6/527	32/ 19594	0.00485	0.004195	GJB3/GJB4/GJB5/ GJB2/CALB2/GJB6	6
GO:0005922	CC	GO:0005922	connexin complex	5/527	21/ 19594	0.00485	0.004195	GJB3/GJB4/GJB5/ GJB2/GJB6	5
GO:0016324	CC	GO:0016324	apical plasma membrane	22/527	358/ 19594	0.006917	0.005982	SLC2A1/SLC16A3/ PTPRH/ANXA1/ RAB27B/PROM2/ CDHR2/ANO1/ CLDN1/UPK1B/ SLC4A11/UMODL1/ AMN/CYP4F12/ GJB6/RHCG/ DUOX2/ABCC2/ LRP2/SLC14A2/ MUC13/SLC15A1	22
GO:0016323	CC	GO:0016323	basolateral plasma membrane	16/527	226/ 19594	0.009342	0.008079	ANXA2/SLC2A1/ SLC16A3/ANXA1/ ITGA3/PROM2/ TGFA/CLDN1/	16

(Continued)

Table S1: *Continued*

	ONTOLOGY	ID	Description	Gene Ratio	Bg Ratio	*p* value	*q* value	gene ID	Count
GO:0045177	CC	GO:0045177	apical part of cell	24/527	424/19594	0.011007	0.009519	CALHM3/SLC4A11/VSIG1/CA9/ADORA1/RHCG/SLCO1B3/CDH17 SLC2A1/SLC16A3/PTPRH/ANXA1/RAB27B/PROM2/CDHR2/ANO1/CLDN1/UPK1B/SLC4A11/UMODL1/AMN/CYP4F12/GJB6/RHCG/DUOXA2/DUOX2/ABCC2/LRP2/SLC14A2/MUC13/SLC15A1/USH1C	24
GO:0031528	CC	GO:0031528	microvillus membrane	5/527	26/19594	0.011077	0.00958	PTPRH/PROM2/CDHR2/CA9/S100P	5
GO:0042383	CC	GO:0042383	sarcolemma	11/527	131/19594	0.015425	0.013341	SLC2A1/AHNAK2/KRT19/ANXA1/PLEC/CLCN1/FLNC/ABCC8/CACNG4/CACNG6/POPDC3	11
GO:0042599	CC	GO:0042599	lamellar body	4/527	17/19594	0.016199	0.01401	KLK7/ABCA12/SFTA3/KLK5	4
GO:0098636	CC	GO:0098636	protein complex involved in cell adhesion	6/527	43/19594	0.016199	0.01401	ITGB4/PLAUR/PLAU/ITGA3/ITGA6/ITGA2	6
GO:0034774	CC	GO:0034774	secretory granule lumen	19/527	322/19594	0.01964	0.016986	ANXA2/CDA/TCN1/UNC13D/SERPINA4/S100A9/SERPINE1/SERPINA3/S100A7/SERPINA1/S100A8/F5/LCN2/SERPINB3/PRSS2/S100P/OLFM4/CRISP3/AOC1	19
GO:0060205	CC	GO:0060205	cytoplasmic vesicle lumen	19/527		0.020958	0.018126		19

(Continued)

Table S1: *Continued*

	ONTOLOGY	ID	Description	Gene Ratio	Bg Ratio	*p* value	*q* value	gene ID	Count
					325/ 19594			ANXA2/CDA/TCN1/ UNC13D/ SERPINA4/S100A9/ SERPINE1/ SERPINA3/S100A7/ SERPINA1/S100A8/ F5/LCN2/ SERPINB3/PRSS2/ S100P/OLFM4/ CRISP3/AOC1	
GO:0031983	CC	GO:0031983	vesicle lumen	19/527	327/ 19594	0.021594	0.018676	ANXA2/CDA/TCN1/ UNC13D/ SERPINA4/S100A9/ SERPINE1/ SERPINA3/S100A7/ SERPINA1/S100A8/ F5/LCN2/ SERPINB3/PRSS2/ S100P/OLFM4/ CRISP3/AOC1	19
GO:0031253	CC	GO:0031253	cell projection membrane	19/527	339/ 19594	0.031258	0.027034	PTPRH/PLEK2/ EPHA2/FSCN1/ ITGA3/FERMT1/ PROM2/CDHR2/ ARHGEF4/EPS8L1/ OPRD1/EPS8L3/ SLC28A3/ADGRV1/ AMN/CA9/ ADORA1/ S100P/LRP2	19
GO:0150034	CC	GO:0150034	distal axon	16/527	270/ 19594	0.037845	0.032731	FSCN1/NGEF/ ITGA3/ITGA2/ TUBB3/SNCG/ OPRD1/NTSR1/ CALB2/L1CAM/TH/ CALCA/ADORA1/ PTPRN/IGF2BP1/ CDK5R2	16
GO:0005902	CC	GO:0005902	microvillus	8/527		0.038606	0.033389		8

(Continued)

Table S1: *Continued*

	ONTOLOGY	ID	Description	Gene Ratio	Bg Ratio	*p* value	*q* value	gene ID	Count
					90/ 19594			MYO1E/PTPRH/ FSCN1/PROM2/ CDHR2/CA9/S100P/ USH1C	
GO:0043679	CC	GO:0043679	axon terminus	9/527	113/ 19594	0.04379	0.037873	ITGA2/SNCG/ OPRD1/NTSR1/ CALB2/TH/CALCA/ ADORA1/PTPRN	9
GO:0043256	CC	GO:0043256	laminin complex	3/527	12/ 19594	0.04379	0.037873	LAMC2/LAMB3/ LAMA3	3
GO:0030280	MF	GO:0030280	structural constituent of skin epidermis	13/512	37/ 18410	5.96×10^{-9}	5.11×10^{-9}	KRT6A/KRT6B/ KRT6C/KRT8/KRT7/ SPRR1A/KRT78/ KRT80/KRT81/PI3/ KRT5/KRT75/PKP1	13
GO:0004252	MF	GO:0004252	serine-type endopeptidase activity	24/512	174/ 18410	3.05×10^{-8}	2.62×10^{-8}	KLK6/PLAU/ MMP14/PRSS22/ MMP11/PRSS3/ MMP1/TMPRSS11E/ KLK7/KLK8/ MMP10/CTSV/ MMP7/TMPRSS4/ PRSS2/PRSS1/ KLK5/TMPRSS11D/ MMP3/KLK12/ KLK14/PCSK9/ PCSK2/HABP2	24
GO:0008236	MF	GO:0008236	serine-type peptidase activity	24/512	191/ 18410	1.43×10^{-7}	1.23×10^{-7}	KLK6/PLAU/ MMP14/PRSS22/ MMP11/PRSS3/ MMP1/TMPRSS11E/ KLK7/KLK8/ MMP10/CTSV/ MMP7/TMPRSS4/ PRSS2/PRSS1/ KLK5/TMPRSS11D/ MMP3/KLK12/ KLK14/PCSK9/ PCSK2/HABP2	24

(Continued)

Table S1: *Continued*

	ONTOLOGY	ID	Description	Gene Ratio	Bg Ratio	*p* value	*q* value	gene ID	Count
GO:0017171	MF	GO:0017171	serine hydrolase activity	24/512	195/18410	1.65×10^{-7}	1.41×10^{-7}	KLK6/PLAU/MMP14/PRSS22/MMP11/PRSS3/MMP1/TMPRSS11E/KLK7/KLK8/MMP10/CTSV/MMP7/TMPRSS4/PRSS2/PRSS1/KLK5/TMPRSS11D/MMP3/KLK12/KLK14/PCSK9/PCSK2/HABP2	24
GO:0004867	MF	GO:0004867	serine-type endopeptidase inhibitor activity	17/512	98/18410	1.88×10^{-7}	1.61×10^{-7}	ANXA2/CD109/SERPINB5/SERPINB7/COL7A1/SERPINA4/SERPINA5/SERPINB4/SERPINE1/SERPINA3/PI3/SERPINA1/SERPINB2/SERPINB3/A2ML1/SPOCK1/AMBP	17
GO:0030414	MF	GO:0030414	peptidase inhibitor activity	23/512	187/18410	2.48×10^{-7}	2.13×10^{-7}	ANXA2/CD109/SERPINB5/SERPINB7/COL7A1/SERPINA4/BIRC3/SERPINA5/CST6/SERPINB4/SERPINE1/TIMP4/SERPINA3/UMODL1/PI3/SERPINA1/SERPINB2/SERPINB3/A2ML1/SPOCK1/CST4/CST1/AMBP	23
GO:0004866	MF	GO:0004866		22/512		4.96×10^{-7}	4.26×10^{-7}		22

(Continued)

Table S1: *Continued*

	ONTOLOGY	ID	Description	Gene Ratio	Bg Ratio	*p* value	*q* value	gene ID	Count
			endopeptidase inhibitor activity		180/ 18410			ANXA2/CD109/ SERPINB5/ SERPINB7/COL7A1/ SERPINA4/BIRC3/ SERPINA5/CST6/ SERPINB4/ SERPINE1/TIMP4/ SERPINA3/PI3/ SERPINA1/ SERPINB2/ SERPINB3/A2ML1/ SPOCK1/CST4/ CST1/AMBP	
GO:0061134	MF	GO:0061134	peptidase regulator activity	25/512	230/ 18410	4.96×10^{-7}	4.26×10^{-7}	ANXA2/CD109/ SERPINB5/PRSS22/ SERPINB7/COL7A1/ SERPINA4/BIRC3/ SERPINA5/CST6/ SERPINB4/ SERPINE1/TIMP4/ SERPINA3/ UMODL1/PI3/ NLRP12/SERPINA1/ SERPINB2/ SERPINB3/A2ML1/ SPOCK1/CST4/ CST1/AMBP	25
GO:0061135	MF	GO:0061135	endopeptidase regulator activity	22/512	194/ 18410	1.66×10^{-6}	1.42×10^{-6}	ANXA2/CD109/ SERPINB5/ SERPINB7/COL7A1/ SERPINA4/BIRC3/ SERPINA5/CST6/ SERPINB4/ SERPINE1/TIMP4/ SERPINA3/PI3/ SERPINA1/ SERPINB2/ SERPINB3/A2ML1/	22

(Continued)

Table S1: *Continued*

	ONTOLOGY	ID	Description	Gene Ratio	Bg Ratio	*p* value	*q* value	gene ID	Count
GO:0004857	MF	GO:0004857	enzyme inhibitor activity	32/512	390/ 18410	3.14×10^{-6}	2.69×10^{-6}	SPOCK1/CST4/ CST1/AMBP ANXA2/CD109/ SH3RF2/ANXA1/ CAMK2N1/ SERPINB5/ SERPINB7/COL7A1/ SFN/SERPINA4/ BIRC3/SERPINA5/ ANXA3/CST6/ SERPINB4/ SERPINE1/TIMP4/ PKIB/SERPINA3/ ADGRV1/GCKR/ UMODL1/PI3/ SERPINA1/ SERPINB2/ SERPINB3/A2ML1/ ANGPTL4/SPOCK1/ CST4/CST1/AMBP	32
GO:0048306	MF	GO:0048306	calcium-dependent protein binding	13/512	87/ 18410	4.37×10^{-5}	3.75×10^{-5}	S100A16/ANXA2/ ANXA1/S100A10/ S100A6/S100A3/ S100A14/ANXA3/ S100A2/S100A9/ S100A7/S100A8/ S100P	13
GO:0005544	MF	GO:0005544	calcium-dependent phospholipid binding	10/512	54/ 18410	0.000101	8.69×10^{-5}	ANXA2/ANXA8/ ANXA1/ANXA8L1/ ANXA3/SYT8/ ANXA10/SYT12/ SYT13/SYT4	10
GO:0048018	MF	GO:0048018	receptor ligand activity	33/512	489/ 18410	0.000128	0.000109	SEMA7A/WNT7A/ UCN2/SEMA3B/ IL1RN/DKK1/EREG/ CLCF1/AREG/TGFA/ IL1A/NDP/IL11/ WNT10A/IFNE/ CXCL11/BMP7/	33

(Continued)

Table S1: *Continued*

	ONTOLOGY	ID	Description	Gene Ratio	Bg Ratio	*p* value	*q* value	gene ID	Count
								CALCB/EDN2/CALCA/TFF1/GREM1/CCL7/DEFB4A/FGF5/GAL/FGF9/CXCL5/INSL4/CXCL6/IL36RN/FGF19/SST	
GO:0030546	MF	GO:0030546	signaling receptor activator activity	33/512	496/18410	0.000161	0.000138	SEMA7A/WNT7A/UCN2/SEMA3B/IL1RN/DKK1/EREG/CLCF1/AREG/TGFA/IL1A/NDP/IL11/WNT10A/IFNE/CXCL11/BMP7/CALCB/EDN2/CALCA/TFF1/GREM1/CCL7/DEFB4A/FGF5/GAL/FGF9/CXCL5/INSL4/CXCL6/IL36RN/FGF19/SST	33
GO:0004175	MF	GO:0004175	endopeptidase activity	30/512	432/18410	0.000176	0.000151	KLK6/PLAU/MMP14/PRSS22/MMP11/PRSS3/MMP1/TMPRSS11E/KLK7/KLK8/MMP10/CTSV/MMP7/TMPRSS4/PRSS2/CTSE/PIP/CLCA2/PRSS1/KLK5/CASP14/TMPRSS11D/MMP3/KLK12/TINAG/KLK14/PCSK9/PCSK2/HABP2/PGC	30
GO:0008201	MF	GO:0008201	heparin binding	17/512	168/18410	0.000176	0.000151	LAMC2/FGFBP1/SERPINA5/ADAMTSL5/LIPH/	17

(Continued)

Table S1: *Continued*

	ONTOLOGY	ID	Description	Gene Ratio	Bg Ratio	*p* value	*q* value	gene ID	Count
								SAA1/CXCL11/ BMP7/CCL7/FGF9/ NELL1/TENM1/ REG4/COL25A1/ CXCL6/ COL11A1/AOC1	
GO:0008391	MF	GO:0008391	arachidonic acid monooxygenase activity	6/512	21/ 18410	0.000604	0.000518	CYP2S1/CYP2C9/ CYP4F12/CYP4F3/ CYP2C18/CYP4F11	6
GO:0005200	MF	GO:0005200	structural constituent of cytoskeleton	12/512	104/ 18410	0.001065	0.000914	KRT16/KRT6A/ KRT6B/KRT19/ PLEC/ACTBL2/VILL/ KRT14/TUBB3/ KRT15/KRT5/KRT20	12
GO:0005539	MF	GO:0005539	glycosaminoglycan binding	18/512	234/ 18410	0.003224	0.002767	LAMC2/FGFBP1/ SERPINA5/ ADAMTSL5/LIPH/ SAA1/CXCL11/ BMP7/CCL7/FGF9/ NELL1/TENM1/ REG4/COL25A1/ CXCL6/COL11A1/ HABP2/AOC1	18
GO:0070851	MF	GO:0070851	growth factor receptor binding	13/512	139/ 18410	0.004116	0.003532	FAM83B/IL1RN/ EREG/AREG/TGFA/ IL1A/CBLC/IL11/ GREM1/FGF5/ FGF9/IL36RN/ FGF19	13
GO:0001664	MF	GO:0001664	G protein-coupled receptor binding	20/512	288/ 18410	0.004968	0.004264	ITGB4/WNT7A/ UCN2/S100A14/ TAC4/NDP/SAA1/ WNT10A/DEFB1/ CXCL11/CALCB/ EDN2/CALCA/CCL7/ TFF2/ADORA1/ DEFB4A/GAL/ CXCL5/CXCL6	20
GO:1901681	MF	GO:1901681	sulfur compound binding	19/512		0.005009	0.004299		19

(Continued)

Table S1: *Continued*

	ONTOLOGY	ID	Description	Gene Ratio	Bg Ratio	*p* value	*q* value	gene ID	Count
					267/ 18410			LAMC2/FGFBP1/ ITGA2/SERPINA5/ ADAMTSL5/LIPH/ SAA1/CXCL11/ BMP7/CCL7/FGF9/ NELL1/TENM1/ REG4/PRMT8/ COL25A1/CXCL6/ COL11A1/AOC1	
GO:0005243	MF	GO:0005243	gap junction channel activity	5/512	21/ 18410	0.005997	0.005146	GJB3/GJB4/GJB5/ GJB2/GJB6	5
GO:0031994	MF	GO:0031994	insulin-like growth factor I binding	4/512	13/ 18410	0.008668	0.007439	ITGB4/ITGA6/ IGFBP1/LRP2	4
GO:0070330	MF	GO:0070330	aromatase activity	5/512	25/ 18410	0.013141	0.011278	CYP2C9/CYP4F12/ CYP4F3/CYP2C18/ CYP4F11	5
GO:0015267	MF	GO:0015267	channel activity	27/512	489/ 18410	0.013781	0.011827	GJB3/GJB4/GJB5/ ANXA2/GJB2/ KCNN4/APOL1/ STEAP1/ANO1/ CLCN1/CALHM3/ SLC4A11/KCNF1/ CATSPER1/ABCC8/ FXYD3/GABRP/ CACNA2D2/CLCA2/ GJB6/RHCG/ HTR3A/KCNV1/ CNGA3/CACNG4/ CACNG6/GABRB3	27
GO:0022803	MF	GO:0022803	passive transmembrane transporter activity	27/512	490/ 18410	0.013781	0.011827	GJB3/GJB4/GJB5/ ANXA2/GJB2/ KCNN4/APOL1/ STEAP1/ANO1/ CLCN1/CALHM3/ SLC4A11/KCNF1/ CATSPER1/ABCC8/ FXYD3/GABRP/ CACNA2D2/CLCA2/ GJB6/RHCG/	

(Continued)

Table S1: *Continued*

	ONTOLOGY	ID	Description	Gene Ratio	Bg Ratio	*p* value	*q* value	gene ID	Count
								HTR3A/KCNV1/CNGA3/CACNG4/CACNG6/GABRB3	
GO:0016712	MF	GO:0016712	oxidoreductase activity, acting on paired donors, with incorporation or reduction of molecular oxygen, reduced flavin or flavoprotein as one donor, and incorporation of one atom of oxygen	6/512	40/18410	0.01606	0.013783	CYP2S1/CYP2C9/CYP4F12/CYP4F3/CYP2C18/CYP4F11	6
GO:0042379	MF	GO:0042379	chemokine receptor binding	8/512	71/18410	0.01606	0.013783	S100A14/DEFB1/CXCL11/CCL7/TFF2/DEFB4A/CXCL5/CXCL6	8
GO:0005125	MF	GO:0005125	cytokine activity	16/512	235/18410	0.018645	0.016002	WNT7A/IL1RN/CLCF1/AREG/IL1A/NDP/IL11/WNT10A/IFNE/CXCL11/BMP7/GREM1/CCL7/CXCL5/CXCL6/IL36RN	16
GO:0008392	MF	GO:0008392	arachidonic acid epoxygenase activity	4/512	17/18410	0.020058	0.017214	CYP2S1/CYP2C9/CYP4F12/CYP2C18	4
GO:0005506	MF	GO:0005506	iron ion binding	12/512	151/18410	0.020058	0.017214	MELTF/XDH/ALOXE3/CYP24A1/CYP2S1/TH/LCN2/CYP2C9/CYP4F12/CYP4F3/CYP2C18/CYP4F11	12
GO:0022829	MF	GO:0022829	wide pore channel activity	5/512	30/18410	0.023447	0.020123	GJB3/GJB4/GJB5/GJB2/GJB6	5
GO:0045236	MF	GO:0045236	CXCR chemokine receptor binding	4/512	18/18410	0.023447	0.020123	CXCL11/TFF2/CXCL5/CXCL6	4
GO:0019838	MF	GO:0019838	growth factor binding	11/512	139/18410	0.029472	0.025294	ITGB4/CD109/FGFBP1/SRPX2/EPHA2/IL1RN/ITGA6/IL1R2/	11

(Continued)

Table S1: *Continued*

	ONTOLOGY	ID	Description	Gene Ratio	Bg Ratio	*p* value	*q* value	gene ID	Count
								IGFBP1/LRP2/ IL36RN	
GO:0005154	MF	GO:0005154	epidermal growth factor receptor binding	5/512	32/ 18410	0.029472	0.025294	FAM83B/EREG/ AREG/TGFA/CBLC	5
GO:0050786	MF	GO:0050786	RAGE receptor binding	3/512	10/ 18410	0.036023	0.030916	S100A9/S100A7/ S100A8	3
GO:0005504	MF	GO:0005504	fatty acid binding	6/512	49/ 18410	0.036047	0.030936	PPARG/S100A9/ FABP6/S100A8/ HNF4A/CYP4F11	6
GO:0005201	MF	GO:0005201	extracellular matrix structural constituent	12/512	172/ 18410	0.049303	0.042313	LAMC2/LAMB3/ COL17A1/SRPX2/ COL7A1/TGFBI/ LAMA3/UMODL1/ MUC5AC/MUC4/ COL25A1/COL11A1	12

Table S2: The results of GSEA

ID	Description	setSize	enrichmentScore	NES	*p* value	*p*.adjust	*q* value	rank	leading_edge	core_enrichment
hsa04061	Viral protein interaction with cytokine and cytokine receptor	98	0.745808	2.006787	1.00×10^{-10}	4.79×10^{-9}	2.06×10^{-9}	6073	tags = 72%, list = 18%, signal = 60%	53833/58985/6374/6354/6373/6372/5473/6366/53832/10344/3606/3627/3576/6355/1232/6364/5196/6367/9547/6356/6359/6357/6376/6362/8809/4283/56477/4055/51554/7132/6348/6352/8795/6369/8797/6351/8793/8743/3560/3559/8740/6346/3561/2833/2919/3577/3569/7133/6347/9560/6846/1436/29949/1234/1237/6368/1230/10563/6363/4049/6375/57007/8764/3588/6361/8807/3586/7124/2921/1435/146433
hsa05164	Influenza A	168	0.647134	1.788176	1.00×10^{-10}	4.79×10^{-9}	2.06×10^{-9}	8459	tags = 65%, list = 25%, signal = 50%	5646/5645/5644/3552/56649/4938/9407/402569/4939/3606/4940/3627/5371/3576/148022/29108/4599/355/64135/1021/91543/6772/7132/637/56000/7416/6352/8795/4615/3458/8797/7098/578/8772/3337/8743/60/3553/3459/71/9021/23586/3838/4600/3569/79671/6347/834/356/64499/8766/5578/7177/9641/5595/4793/836/1965/5604/54205/3716/4790/7187/208/5605/3117/1019/5291/3717/3592/3665/7099/3111/3123/3122/841/5290/3127/581/3836/5970/3112/7124/8717/5293/5610/8517/842/11100/293/6773/3113/3439/29107/3115/7706/3661/114548/6041/207/29110/9230/3383/5611/2224/896/100529063/292/23633/3109
hsa04060	Cytokine-cytokine receptor interaction	293	0.6261	1.759532	1.00×10^{-10}	4.79×10^{-9}	2.06×10^{-9}	6318	tags = 52%, list = 18%, signal = 43%	53833/58985/3552/7850/6374/3557/655/26525/338376/64109/6354/6373/6372/51330/3589/133396/23529/5473/6366/

(Continued)

Table S2: *Continued*

ID	Description	setSize	enrichmentScore	NES	*p* value	*p*.adjust	*q* value	rank	leading_edge	core_enrichment
										51561/53832/10344/3606/970/8744/27242/3627/9235/56300/3576/6355/1232/3601/6364/5196/6367/9547/6356/6359/355/6357/130399/1438/6376/6362/3574/3604/3566/8809/3623/4283/56477/4055/9180/51554/7132/9173/8600/6348/3624/6352/8795/6369/3458/8797/8784/268/6351/8793/8743/10148/3560/3559/958/3553/8740/6346/3459/3561/9466/7040/7292/2833/2919/3577/3600/3569/27190/282617/7133/6347/85480/246778/4050/356/9560/3595/658/6846/1436/10673/7046/652/1437/3594/50615/29949/58191/1234/3952/1237/6368/90/7293/1230/10563/282616/282618/6363/943/4049/8741/3976/6375/8200/5008/920/57007/4982/8764/3588/3556/6361/3597/3592/8807/3581/3625/94/8792/3586/10663/7048/7124/2921/3575/7042/1435/146433/1439/84957/23765
hsa05130	Pathogenic Escherichia coli infection	196	0.627716	1.746466	1.00×10^{-10}	4.79×10^{-9}	2.06×10^{-9}	8144	tags = 65%, list = 24%, signal = 50%	10381/9076/4643/57121/4640/7277/9071/84617/3606/3576/2597/29108/79861/355/2147/3688/4645/64005/10686/84790/10095/55971/7132/5600/10376/23562/8795/4615/8797/578/8772/8743/837/4627/60/7430/3553/71/10552/9368/4690/10458/79784/7122/644150/10109/3569/23191/5062/2768/5879/4641/6300/834/356/10096/1364/9170/3654/9080/203068/6714/

(Continued)

Table S2: *Continued*

ID	Description	setSize	enrichmentScore	NES	*p* value	*p*.adjust	*q* value	rank	leading_edge	core_enrichment
										10093/10787/55930/382/10097/5058/5595/4793/836/10383/9074/54205/1366/7082/10094/27128/4790/7456/387/2212/2149/81873/4629/10163/7099/841/2017/581/8440/5777/10092/5970/10061/7124/7846/8717/8517/4642/840/23118/842/2846/9267/10672/9138/5603/10006/4691/7009/8976/10802/5861/4644/4542/10427/3071/9871/114548/5781/9266/9632/1432/8737/147179/55845/25
hsa05169	Epstein-Barr virus infection	198	0.62326	1.734325	1.00×10^{-10}	4.79×10^{-9}	2.06×10^{-9}	8130	tags = 64%, list = 24%, signal = 49%	4938/8900/898/4939/9636/4940/3627/3135/6890/1026/890/4609/355/6891/3134/5971/1647/1021/6772/3106/4734/637/3105/7128/5600/3107/567/4615/4791/578/8772/595/960/958/4616/5708/965/5714/4794/23586/5704/3569/5879/6300/4067/915/6892/3654/3133/377841/864/7431/9641/10213/4793/11047/836/54205/3716/5606/1870/4790/7187/208/919/916/1017/3117/1019/5701/5291/894/3718/9612/3665/3111/29760/3123/5707/917/3122/841/5290/3127/581/5713/5970/3112/7124/1869/8717/5293/5610/8517/5705/23118/842/3066/5717/5709/5700/9541/5925/5603/3065/22938/3280/5702/6773/3113/3439/9020/1643/5719/3115/3661/5718/207/29110/1432/811/8737/3383/896/5706/5609/2923
hsa05170		210	0.617054	1.721493	1.00×10^{-10}	4.79×10^{-9}	2.06×10^{-9}	8502		

(Continued)

Table S2: *Continued*

ID	Description	setSize	enrichmentScore	NES	*p* value	*p*.adjust	*q* value	rank	leading_edge	core_enrichment
	Human immunodeficiency virus 1 infection								tags = 63%, list = 25%, signal = 48%	200315/3135/130340/6890/598/5829/5582/9133/355/6891/3134/891/27350/995/5880/3106/9582/7132/983/637/60489/3105/5600/3107/85417/567/4615/3984/578/8772/55970/10298/140564/1072/1174/2786/2771/1111/5062/7133/5879/6300/356/200316/2793/808/6923/915/115004/5578/684/6892/3654/3133/2767/2790/1234/51806/59345/5058/5595/91860/836/2773/5604/54205/9616/25939/2783/10053/5606/4790/920/208/919/164668/5605/162/3985/916/5291/7099/917/841/5290/581/1399/56924/4893/5970/7124/8717/4772/5293/8517/5530/23118/842/5603/340061/10681/2185/805/3439/3265/6921/3845/6500/8451/3661/3710/8450/8907/8454/207/29110/6199/1432/811/8737/7852/2782/5609/2923/801/4773/9978/545/51764/1398/7186/8945
hsa04510	Focal adhesion	202	0.614908	1.713386	1.01×10^{-10}	4.79×10^{-9}	2.06×10^{-9}	6081	tags = 52%, list = 18%, signal = 43%	3918/3691/3914/3909/4233/3673/2318/3381/330/3675/3655/3696/3690/858/3371/1291/2012/3694/7424/1292/3678/1277/857/6696/7791/5829/5582/7058/9564/7408/5155/81/7414/87/3688/3693/5880/22801/3685/2316/3912/1278/1293/7410/2335/10627/5154/595/10398/1284/10298/60/71/284217/824/10319/4638/5062/25759/5879/1282/29780/1729/103910/22798/5578/7057/6714/5159/1499/5058/5595/3695/2317/

(Continued)

Table S2: *Continued*

ID	Description	setSize	enrichmentScore	NES	*p* value	*p*.adjust	*q* value	rank	leading_edge	core_enrichment
										3910/1288/23396/329/5604/3611/5908/2064/6464/3672/208/56034/387/7409/10451/5228/3911/5291/894/3480/2885/5499/7422/5290/1399/56924/256076/2324/5293/1299/1287/2002
hsa05132	Salmonella infection	249	0.58128	1.627369	3.84×10^{-10}	1.59×10^{-8}	6.87×10^{-9}	9078	tags = 63%, list = 26%, signal = 47%	113146/10381/2318/302/330/6281/7277/84617/3606/3576/2597/6237/29108/4609/79861/79026/838/8767/338382/84790/2316/197259/10095/7132/83657/929/57617/5600/10376/8795/4615/8797/10627/578/8772/8743/837/10398/60/51429/3553/71/5287/10552/3798/10109/3569/10333/23191/391/5879/6300/834/10096/103910/3654/4074/11035/203068/5216/8655/7879/3840/10093/6932/10787/382/10097/1499/5058/5595/1781/2317/7295/836/10383/329/5604/54205/5585/5606/10094/27128/4790/5878/3320/208/5898/387/5605/9367/81873/5291/3839/7099/841/5290/10121/581/3836/10092/1785/60412/5970/7124/7846/8717/5293/8517/257364/840/23118/9267/9842/3326/5217/5603/147700/10006/8677/10540/5420/8976/55207/83547/84516/3265/6990/79792/3071/23643/6500/25828/23207/114548/10120/207/9266/1432/8737/5609/55845/10671/83658/58484/5868/58498/5869/7186/57381/26263/55770/1434/6934/1639/6885/64837

(Continued)

Table S2: *Continued*

ID	Description	setSize	enrichmentScore	NES	*p* value	*p*.adjust	*q* value	rank	leading_edge	core_enrichment
hsa04650	Natural killer cell mediated cytotoxicity	124	0.659872	1.794665	4.35×10^{-10}	1.60×10^{-8}	6.92×10^{-9}	8682	tags = 67%, list = 25%, signal = 50%	154064/135250/80328/353091/3821/3135/3822/3002/79465/5582/355/117157/3823/9437/5880/3106/2214/4277/637/3105/3107/8795/3458/8797/7410/3824/8743/2207/3459/10870/3689/2215/7305/25759/5879/7462/356/5578/3932/3133/1437/5058/5595/836/5604/6464/919/5551/100507436/7409/5605/10451/5291/6452/2885/3937/5290/5777/4893/7124/3809/4772/5293/3804/5530/80329/399694/2185/259197/3384/3439/4068/3265/3845/51744/5781/3383/3811/4773/22914/3812/962/7535
hsa04621	NOD-like receptor signaling pathway	183	0.610206	1.693227	9.32×10^{-10}	3.09×10^{-8}	1.33×10^{-8}	7897	tags = 56%, list = 23%, signal = 43%	9447/4938/91662/330/1673/4939/2633/3606/2635/4940/3428/3576/2634/148022/29108/598/838/8767/6772/84674/118429/115361/7128/10135/7416/5600/6352/1508/4615/8772/4210/7205/837/1535/3553/7417/2919/115362/51393/3569/79671/114769/6300/6347/64127/834/5331/11035/9641/24145/55054/5595/4793/7295/10616/329/5585/3716/9474/84168/4790/3320/7187/387/10010/9051/1536/23710/3665/7099/841/90550/10628/5970/199713/10059/7124/2921/8517/23118/5332/81858/3326/9140/5603/340061/6773/3439/79792/5027/7531/3661/64170/3710/25828/9927/55669/114548/6041/29110/1432/8737
hsa05417	Lipid and atherosclerosis	214	0.581654	1.623549	1.55×10^{-9}	4.68×10^{-8}	2.02×10^{-8}	8191		

(Continued)

Table S2: *Continued*

ID	Description	setSize	enrichmentScore	NES	*p* value	*p*.adjust	*q* value	rank	leading_edge	core_enrichment
									tags = 57%, list = 24%, signal = 43%	4312/5468/3929/1559/4314/30001/3606/3576/148022/29108/598/4318/355/3949/6648/7132/637/929/6348/4688/5600/6352/8795/4615/8797/7410/3310/7412/8743/958/1535/3553/337/2919/1555/4217/3569/10333/25833/5879/6300/6347/4067/3312/834/356/808/5578/4973/5331/3654/6714/9641/51806/5595/91860/836/1965/54205/5908/3308/5606/4790/4689/3320/7187/208/3303/387/10010/7409/19/1536/353376/10451/5291/3717/3592/3665/7099/841/653361/5290/581/4893/3329/5970/7124/2921/4772/5293/8517/840/5530/23118/3304/842/5332/6256/3326/839/9138/4780/5603/3306/6349/805/3439/3265/23643/3845/818/3661/114548/207/29110/1432/3383/5609/801/4773
hsa05163	Human cytomegalovirus infection	223	0.588852	1.643234	2.51×10^{-9}	6.95×10^{-8}	3.00×10^{-8}	8477	tags = 59%, list = 25%, signal = 45%	3690/3135/6890/3576/1026/1232/5829/5582/4609/9564/355/6891/3134/1021/6376/5880/3685/3106/7132/637/3105/6348/5600/3107/6352/567/578/6351/8772/595/55970/3553/2786/2771/1978/3569/84699/2768/5879/6300/6347/356/9560/2793/808/115004/5578/6892/5331/3133/109/2767/6714/2790/1234/51806/59345/1499/5595/91860/836/2773/5604/54205/1230/3716/2783/1870/4790/208/387/5605/3588/1019/5291/2885/5732/6667/841/9586/5731/7422/

(Continued)

Table S2: *Continued*

ID	Description	setSize	enrichmentScore	NES	*p* value	*p*.adjust	*q* value	rank	leading_edge	core_enrichment
										5290/581/1399/4893/5970/7124/1869/8717/4772/5293/8517/2002/5530/5734/842/10488/5332/10672/5925/9138/5603/3579/340061/10681/2185/6349/805/3439/3265/3845/3661/3710/5156/5743/207/29110/6199/1432/811/8737/7852/2782/2923/801/4773/51764/1398/7186/1956
hsa05166	Human T-cell leukemia virus 1 infection	219	0.583782	1.628761	5.31×10^{-9}	1.36×10^{-7}	5.84×10^{-8}	10165	tags = 65%, list = 30%, signal = 46%	8061/6513/4488/4316/7850/8900/898/3135/1026/3601/598/890/4609/9133/991/4085/3134/706/5971/701/4088/3106/4055/7132/3105/7416/3107/567/4791/595/3560/3559/958/3561/7417/7040/9700/3689/3600/1111/3569/84699/6688/6776/5901/915/2113/3932/3133/7046/1437/109/9232/7538/5902/5595/1958/5604/1959/4487/3716/4049/1870/9184/4790/920/208/5605/996/916/8379/1017/6722/3117/1019/5291/894/3718/6929/55697/3111/3123/917/3122/9586/5290/3127/581/4893/7048/5970/3112/7124/1869/4772/5293/7042/8517/2002/5530/10488/5925/1030/7094/293/3113/3265/9020/3845/64682/3115/51529/8881/207/7043/811/3383/2224/896/4773/25847/292/545/3109/196883/7514/5566/10393/246184/5728/1029/83660/5594/1871/5534/90993/3683/821/6908/5567/200186/7157/1031
hsa05146	Amoebiasis	101	0.667572	1.798626	5.73×10^{-9}	1.36×10^{-7}	5.86×10^{-8}	6268	tags = 61%, list = 18%, signal = 50%	3918/6318/6317/3914/3909/4583/7850/2769/3576/1277/5275/5582/1281/81/7414/

(Continued)

Table S2: *Continued*

ID	Description	setSize	enrichmentScore	NES	*p* value	*p*.adjust	*q* value	rank	leading_edge	core_enrichment
										87/338382/5272/3912/735/1278/929/3458/2335/1284/3553/284217/7040/10319/2919/3689/3684/3569/1282/4843/22798/5578/5331/5269/1437/2767/7879/3910/836/1288/4790/3315/5878/3911/5291/3592/7099/5052/5290/3586/5970/7124/2921/5293/7042/1287/5332
hsa05167	Kaposi sarcoma-associated herpesvirus infection	194	0.587721	1.634326	8.08×10^{-9}	1.79×10^{-7}	7.71×10^{-8}	9803	tags = 64%, list = 29%, signal = 46%	3135/3576/1026/1232/148022/4609/355/3134/5155/1021/6772/3106/7132/4277/637/3105/5600/3107/3091/7098/578/8772/595/55970/9976/3459/7316/2919/2786/718/3569/3055/5879/6300/4067/2793/808/3133/1437/6714/2790/9641/1234/6932/51806/7538/59345/942/2247/1237/1499/5595/91860/836/5604/54205/1230/3716/2783/1870/4790/7187/208/100507436/5605/1019/5291/3717/3665/841/7422/5290/581/4893/5970/1869/8717/2921/4772/5293/5610/8517/64422/5530/842/7311/8678/6233/5925/5603/10681/805/9261/6773/3439/3265/3845/57580/3661/3710/5743/207/29110/1432/3383/2782/5609/801/4773/81631/51764/146850/7186/6934/440738/23533/6850/2920/344807/2932/5594/1871/3455/5534
hsa05165	Human papillomavirus infection	331	0.540989	1.525972	9.24×10^{-9}	1.92×10^{-7}	8.26×10^{-8}	7814	tags = 49%, list = 23%, signal = 38%	7476/3918/3691/3914/54626/5522/3909/3673/8638/80326/3381/3675/8900/3655/7477/898/3696/3690/3371/9636/1291/3694/

(Continued)

Table S2: *Continued*

ID	Description	setSize	enrichmentScore	NES	*p* value	*p*.adjust	*q* value	rank	leading_edge	core_enrichment
										1292/3135/3678/1026/148022/1277/6696/5829/5315/890/7058/4599/355/3134/4854/57801/1021/182/3688/3693/22801/6772/3685/3659/3106/3912/7132/1278/3105/89780/3107/1293/2335/7098/578/8772/595/1284/284217/1855/9368/10319/1978/4600/527/84699/1282/356/22798/1452/2535/3133/7057/3996/9641/6932/10297/5159/1499/5595/3695/11211/3910/836/1288/7283/5604/3716/3672/9296/4790/84667/84441/7187/208/529/10312/10134/2308/7483/7475/5605/5515/5518/1017/1019/3911/5291/10474/894/5663/2885/56288/841/9586/5520/7422/5290/5526/581/51382/256076/4893/3993/5970/7124/1869/8717/8312/5293/5610/8517/1299/1287/1857/245972/5734/3066/10488/5700/5925/5521/92359/3065/3280/6773/3439/3265/8324/3845/8992/7448/5516/3661/526/5743/7337/7472/207/29110/6199
hsa04613	Neutrophil extracellular trap formation	184	0.596347	1.655432	1.01×10^{-8}	1.98×10^{-7}	8.55×10^{-8}	7180	tags = 52%, list = 21%, signal = 41%	3690/5582/3013/5880/8329/2357/2214/8351/3012/55506/8347/7416/4688/5600/8339/8334/2358/55766/837/366/8345/60/1535/71/728/2209/7417/4353/8970/3689/8357/6404/718/2215/2359/3684/8343/128312/5879/6300/10105/834/3014/27180/5578/85236/5331/23569/92815/10533/3015/6714/1182/3017/8969/8370/5595/5604/

(Continued)

Table S2: *Continued*

ID	Description	setSize	enrichmentScore	NES	*p* value	*p*.adjust	*q* value	rank	leading_edge	core_enrichment
										8355/2266/8353/8348/4790/79885/2243/4689/208/3021/2212/5605/1536/64581/5291/440689/9734/7099/51564/8349/653361/5290/8520/51311/5970/8362/5293/3066/5332/5603/3146/9555/3065/293/440093/8294/79792/8367
hsa03010	Ribosome	154	0.61366	1.687476	1.13×10^{-8}	2.07×10^{-7}	8.90×10^{-8}	11719	tags = 77%, list = 34%, signal = 51%	6183/29088/6142/200916/11224/6223/28998/6182/55173/6231/100529239/6175/25873/6165/6218/6132/64963/11222/6158/6217/124995/10573/63875/6193/6206/51187/6159/51073/65003/6209/64979/6141/51121/6208/51116/6143/23521/51065/3921/6155/51023/6187/29074/6152/51318/6124/6171/7311/55168/6233/6203/6150/64983/51021/6192/6161/9553/65008/6137/6222/9045/6136/6205/51069/6204/6144/6235/6176/29093/6181/6169/64981/51081/2197/6164/9801/6166/6122/6157/64960/6133/219927/64928/6130/65005/6156/6147/6234/4736/6134/6154/6210/6170/51373/6189/6129/51264/64965/6201/6202/51263/6207/6168/6229/9349/140032/6228/6188/6167/6160/6139/6230/6138/6128/6224/79590/54948/6227/6125
hsa05131	Shigellosis	245	0.567659	1.588547	1.18×10^{-8}	2.07×10^{-7}	8.90×10^{-8}	8502	tags = 56%, list = 25%, signal = 43%	113026/3606/80201/3678/3576/29108/598/5829/3099/9564/81/7414/87/8767/3688/10318/8915/8351/10095/7132/929/7416/5600/6352/826/4615/823/10627/837/10398/

(Continued)

Table S2: *Continued*

ID	Description	setSize	enrichmentScore	NES	*p* value	*p*.adjust	*q* value	rank	leading_edge	core_enrichment
										60/960/3553/71/10552/824/7316/8357/718/10109/831/5879/6300/1729/84335/834/23176/3101/3098/10096/103910/10801/115004/5331/1437/5216/6714/10093/5333/55054/382/10097/5595/4793/2931/10616/3611/54205/8355/10094/9474/8353/27128/4790/208/7321/387/3021/2308/5481/81873/55752/5291/10163/7099/5290/2017/581/1399/10092/5970/8878/7124/8717/5293/8517/23118/7311/9267/5332/8678/6233/81858/9140/5217/5603/340061/7094/55062/8976/7323/23048/440093/6500/3661/3710/114548/8454/207/29110/9266/6199/1432/30849/8737/26100/81631/9978/4735/58484/57521/989/58498/11335/1398/7186/1956/8945
hsa04810	Regulation of actin cytoskeleton	228	0.574729	1.605391	1.26×10^{-8}	2.09×10^{-7}	9.02×10^{-8}	7066	tags = 53%, list = 21%, signal = 42%	3691/50649/3673/9965/3675/2250/3655/57121/3696/3690/3694/3678/6237/5829/9564/5155/81/7414/87/2147/3688/128239/3693/5880/22801/3685/10095/81624/735/7410/2934/2335/3984/10627/5154/55970/4627/10398/10298/60/7430/71/10552/7074/1072/10458/3689/79784/9087/3684/54961/10109/4638/23191/5062/2768/3827/5879/6548/1729/79837/10096/103910/624/9170/5216/6714/10093/10787/1132/10297/10097/5159/2247/8826/5058/5595/3695/23396/5604/623/3672/10094/4478/208/56034/

(Continued)

Table S2: *Continued*

ID	Description	setSize	enrichmentScore	NES	*p* value	*p*.adjust	*q* value	rank	leading_edge	core_enrichment
										387/7409/5605/2149/3985/54434/2246/81873/10451/5305/5291/4629/10163/3681/5962/5499/5290/1399/56924/10092/4893/3687/5293/3682/2846/10672/28964/5217/9138/8976/2252/3265/3071/3845
hsa04380	Osteoclast differentiation	125	0.637314	1.733045	1.50×10^{-8}	2.27×10^{-7}	9.78×10^{-8}	9846	tags = 74%, list = 29%, signal = 53%	8061/2274/5468/3552/3690/2355/5971/6772/3726/2214/7132/140885/8600/4688/5600/353514/3458/4791/11006/1513/1535/3553/3459/2209/7040/126014/4286/9021/55423/54209/10288/2215/10326/7305/6688/5879/6300/79168/1436/3932/7046/5595/5604/3716/4790/4689/23547/208/4982/2212/11024/5291/2885/29760/3937/653361/5290/8792/10990/7048/5970/8878/7124/4772/5293/7042/8517/1435/5530/23118/54/10859/5603/6773/9020/11025/2213/207/1432/5609/4773/7186/8651/27035/6885/3727/6850/2354/5594/3455/5534/3460
hsa05203	Viral carcinogenesis	202	0.586887	1.635309	1.44×10^{-8}	2.27×10^{-7}	9.78×10^{-8}	10799	tags = 68%, list = 31%, signal = 47%	8900/898/5366/3135/1026/1232/5829/5315/890/991/3134/81/87/1021/3106/4055/8347/983/3105/3107/8339/2934/4791/578/595/7534/8345/7532/8970/6672/718/1111/8343/128312/84699/5879/4067/6776/7533/1960/85236/3133/6714/3017/1234/8370/1237/5902/5595/836/1959/3716/8348/4790/79885/7187/387/8379/7529/2961/1017/6722/1019/5291/440689/894/3718/55697/2885/

(Continued)

Table S2: *Continued*

ID	Description	setSize	enrichmentScore	NES	*p* value	*p*.adjust	*q* value	rank	leading_edge	core_enrichment
										3665/9734/51564/841/9586/8349/5290/581/2958/4893/5970/8717/8362/5293/5610/8517/245972/3066/10488/27044/5700/5925/1030/2960/3065/22938/9261/3265/8294/3845/8367/7531/3661/3190/2967/10971/7337/8841/896/7185/23513/5922/7186/2959/8365/5933/2965/5566/6502/6850/1029/1642/5594/1108/90993/9093/4193/6908/998/1027/5567/7157/7874/8363/8360/2033/468/9114/1739
hsa05160	Hepatitis C	157	0.61323	1.684649	1.66×10^{-8}	2.39×10^{-7}	1.03×10^{-7}	7897	tags = 58%, list = 23%, signal = 45%	5522/4938/9076/9071/4939/4940/3627/1026/148022/4609/4599/355/1021/91543/10686/6772/3949/3434/7132/637/949/23562/3458/7098/578/8772/595/7534/7532/9021/7122/23586/4600/356/10197/7533/1364/9080/9641/1499/5595/836/9074/1965/5604/54205/3716/1366/1870/4790/7187/208/5605/5515/5518/7529/1017/1019/5291/2885/3665/841/5520/5290/581/440275/4893/5970/7124/1869/8717/5293/5610/8517/842/6256/8837/5925/5521/6773/3439/3265/3845/7531/5516/3661/10971/6041/207/29110/8737
hsa04144	Endocytosis	251	0.561071	1.570493	2.25×10^{-8}	2.87×10^{-7}	1.24×10^{-7}	8744	tags = 61%, list = 25%, signal = 46%	23624/1212/858/100526767/3135/5371/8853/857/55040/30846/3134/116984/4088/3949/3106/26056/10095/1173/4734/79720/10938/11031/3105/6455/3107/3310/30844/1175/3560/3559/3561/10552/50807/160/

(Continued)

Table S2: *Continued*

ID	Description	setSize	enrichmentScore	NES	*p* value	*p*.adjust	*q* value	rank	leading_edge	core_enrichment
										57154/3798/3577/644150/10109/29924/30011/5337/3312/92421/6457/11021/830/25978/5119/10096/2350/9146/8766/829/3133/7046/10890/7879/6714/10093/4218/1601/163/1234/7037/382/10097/64411/80223/23396/378/1759/274/10094/27128/23096/27243/128866/10015/2870/5878/7456/8724/3303/387/81873/377/440073/832/51510/381/116986/3480/8027/56288/9559/93343/10092/1785/7048/30845/23527/57132/84313/3304/156/9267/11267/51534/10617/51652/28964/9897/51160/26119/91782/3579/83737/3306/8976/80230/51100/3265/8729/9525/2352/5156/29934/79643/11059/7251/9266/56904/6643/9230/147179/7852/26286/9922/23550/254122/5868/118813/23111/5338/5869/1956/2261/9744/9101/1211/868/23325
hsa05171	Coronavirus disease – COVID-19	231	0.55975	1.563892	2.17×10^{-8}	2.87×10^{-7}	1.24×10^{-7}	10390	tags = 67%, list = 30%, signal = 47%	4312/4938/4314/4939/9636/4940/3627/3576/5582/4599/629/64135/2147/6772/1839/714/715/7132/735/712/713/5600/4615/716/7098/2162/3553/728/719/23586/718/4600/3569/6300/6347/6142/834/115004/5578/3654/200916/1437/11224/9641/6223/5595/4793/6231/100529239/3716/2266/6175/4790/25873/1636/6165/6868/2243/6218/7187/6132/6158/6217/2212/1536/6193/5291/3592/6206/51187/6159/6209/7099/

(Continued)

Table S2: *Continued*

ID	Description	setSize	enrichmentScore	NES	*p* value	*p*.adjust	*q* value	rank	leading_edge	core_enrichment
										6141/51121/6208/5290/6143/51311/23521/51065/5970/3921/6155/7124/5293/5610/6187/8517/6152/23118/6124/1675/6171/7311/6233/6203/5603/340061/6192/6161/6773/3439/6137/6222/9045/6136/3661/6205/6204/6144/6235/6176/6181/114548/6169/29110/2197/1432/6164/6166/6122/6157/6133/6130/1956/6156/6147/6234/51284/6885/4736/103/6134/6154/6850/6210/6170/5594/6189/5648/6129/3455/6201/6202/6207/6168/6229/9349/140032/6228/6188/59272/6167
hsa04151	PI3K-Akt signaling pathway	352	0.530021	1.495264	2.14×10^{-8}	2.87×10^{-7}	1.24×10^{-7}	8490	tags = 50%, list = 25%, signal = 38%	2069/3918/3691/374/3914/5522/3909/1943/4233/1969/3673/54541/7039/3381/9965/3675/2250/3655/57121/898/3696/3690/3371/1946/1291/3694/7424/1292/3678/1026/1277/6696/598/4909/4609/7058/5155/1021/3688/3693/3574/22801/3685/3566/9180/3912/1278/3667/1293/2335/5154/118788/595/55970/7534/1284/3560/3559/672/3561/284217/7532/10319/253314/2786/11140/1978/6446/3569/84699/5879/1282/8115/356/2793/22798/7533/1436/5578/9170/7057/2790/10110/59345/5159/2247/5595/3695/3910/1288/5604/2064/5585/3716/2783/3672/3481/4790/5008/3320/1944/208/56034/5605/2149/5515/5518/5563/7529/2997/2246/1017/5228/51378/1019/

(Continued)

Table S2: *Continued*

ID	Description	setSize	enrichmentScore	NES	*p* value	*p*.adjust	*q* value	rank	leading_edge	core_enrichment
										3911/5291/894/3718/3717/3480/2885/7099/4170/9586/5520/7422/4908/5290/5526/627/256076/4893/29941/5970/2324/3575/5293/8517/1299/1435/1287/842/10488/2846/6256/1977/3326/5521/10681/2252/3439/3265/3845/6794/7531/7448/5516/2998/2321/57818/5156/10971/8074/207/6199/2065/64223/896/2323/2782/51764/10161/57521/146850/1956/3915
hsa04668	TNF signaling pathway	114	0.640232	1.733925	2.68×10^{-8}	3.29×10^{-7}	1.42×10^{-7}	8466	tags = 69%, list = 25%, signal = 52%	6374/6372/330/4314/4323/1906/7424/3627/6364/4318/355/182/6376/8809/3659/8986/3726/197259/7132/7128/5600/6352/7412/8772/3553/9021/2919/3600/4217/3569/84699/7133/6300/6347/64127/602/1051/1437/11035/5595/836/329/5604/153090/5606/4049/3976/4790/7187/208/5291/841/9586/5290/192111/5970/10059/7124/8717/2921/5293/8517/1435/840/23118/10488/8837/5603/83737/9020/5743/207/1432/8737/3383/7185/5609/9530/7186
hsa04110	Cell cycle	127	0.623177	1.694145	3.81×10^{-8}	4.52×10^{-7}	1.95×10^{-7}	10491	tags = 77%, list = 31%, signal = 54%	2810/8900/898/5347/990/1026/9088/890/4609/9133/991/4085/8318/891/1647/1021/701/7272/995/4088/4998/983/993/85417/4174/699/595/7534/4173/4616/4171/7532/7040/9700/1111/4175/7533/9232/23594/4176/51343/7027/1870/9184/1874/5001/1028/996/8379/7529/1017/4172/1019/894/5111/10459/1022/1869/7042/3066/10926/5925/1030/3065/64682/6500/7531/10971/

(Continued)

Table S2: *Continued*

ID	Description	setSize	enrichmentScore	NES	*p* value	*p*.adjust	*q* value	rank	leading_edge	core_enrichment
										51529/8881/8454/7043/896/25/25847/9978/545/4999/9126/10274/5933/10393/6502/246184/1029/23595/2932/5591/1871/8317/4193/1027/902/7157/1031/8555/1032/2033
hsa04657	IL-17 signaling pathway	93	0.662244	1.7786	4.98×10^{-8}	5.51×10^{-7}	2.38×10^{-7}	6503	tags = 56%, list = 19%, signal = 45%	4586/8061/4312/6278/727897/6280/3934/6374/6279/6354/6372/1673/4314/4322/3627/3576/6364/6356/4318/338324/7128/5600/3458/8772/5597/3553/2919/3569/27190/6300/6347/10758/1051/1437/9641/5595/836/4790/3320/7187/6361/841/5596/5970/7124/8717/2921/8517/23118/23765/3326/5603
hsa05135	Yersinia infection	136	0.62505	1.70847	4.90×10^{-8}	5.51×10^{-7}	2.38×10^{-7}	8466	tags = 64%, list = 25%, signal = 48%	3606/3678/3576/148022/29108/5829/9564/3688/5880/10095/5600/4615/7410/2335/3984/4210/60/8935/3553/71/10552/10458/925/644150/10109/3569/2533/391/5879/6300/6347/834/10096/3932/3654/6714/10093/382/10097/5595/23396/5604/5585/5606/10094/4790/7456/920/208/387/2212/7409/5605/81873/10451/5291/7454/10163/7099/926/3937/5290/1399/10092/3586/5970/7124/4772/5293/8517/23118/9138/5603/2185/8976/27330/3661/114548/207/29110/1432/147179/5609/4773/58484/1398/7186
hsa05150	Staphylococcus aureus infection	88	0.673097	1.804591	5.24×10^{-8}	5.61×10^{-7}	2.42×10^{-7}	2780	tags = 39%, list = 8%, signal = 36%	3868/3872/3861/25984/1672/3880/3860/3866/54474/1673/3859/5724/3875/629/

(Continued)

Table S2: *Continued*

ID	Description	setSize	enrichmentScore	NES	*p* value	*p*.adjust	*q* value	rank	leading_edge	core_enrichment
										2357/714/2214/715/712/713/3857/716/2358/3885/728/2209/3689/3075/6404/719/718/2215/2359/3684
hsa04218	Cellular senescence	156	0.601742	1.653354	6.16×10^{-8}	6.39×10^{-7}	2.76×10^{-7}	9898	tags = 70%, list = 29%, signal = 50%	3552/8900/5054/898/3135/3576/3486/1026/6237/3805/890/2305/4605/4609/9133/3134/59341/891/1647/1021/4088/3106/983/993/3105/7416/5600/3107/85417/823/595/4616/4683/7417/824/7040/1978/678/1111/3569/6300/10758/808/2113/3133/7046/51806/5595/91860/5604/147746/5606/1870/4790/208/1874/2308/5605/5481/1017/1019/5291/894/5499/90550/5290/4893/7048/5970/8878/1869/4772/5293/7042/5530/144715/5925/5603/1030/805/9261/293/3265/3845/3710/207/7043/1432/55957/896/801/4773/292/545/10111/8945/677/5933/2626/5500/5501/5728/1029/2309/5594/1871/23291/5534/4193
hsa05161	Hepatitis B	162	0.593678	1.636774	7.21×10^{-8}	7.26×10^{-7}	3.13×10^{-7}	8191	tags = 56%, list = 24%, signal = 42%	8900/898/3576/1026/148022/5582/890/4609/332/4318/355/64135/6772/4088/637/5600/4615/7098/8772/7534/7040/23586/3569/84699/6300/6776/356/3339/1960/5578/3654/7046/6714/9641/5595/836/5604/54205/1959/3716/5606/1870/4790/7187/208/5605/7529/353376/1017/5291/3718/5111/3717/2885/3665/7099/841/9586/5290/581/4893/6778/7048/5970/7124/1869/

(Continued)

Table S2: *Continued*

ID	Description	setSize	enrichmentScore	NES	*p* value	*p*.adjust	*q* value	rank	leading_edge	core_enrichment
										4772/5293/7042/8517/2002/23118/842/10488/5925/5603/2185/6773/3439/3265/3845/1643/3661/10971/207/7043/29110/1432/5609/4773
hsa04062	Chemokine signaling pathway	190	0.586227	1.630316	8.13×10^{-8}	7.94×10^{-7}	3.42×10^{-7}	7101	tags = 54%, list = 21%, signal = 43%	6374/6354/6373/6372/5473/6366/10344/3627/3576/6355/1232/6364/5196/6367/5829/9547/6356/6359/9564/6357/6376/6362/5880/6772/4283/56477/6348/6352/6369/7410/6351/55970/6346/7074/2833/2919/3577/2786/2771/3055/25759/5879/6347/4067/9560/6846/2793/5331/109/6714/58191/2790/1234/59345/1237/5058/5595/4793/2931/6368/2773/5604/5908/1230/10563/6464/6363/2783/6375/4790/2870/208/2268/387/7409/10451/5291/7454/6361/3718/3717/2885/56288/653361/5290/1399/4893/10663/5970/2921/5293/8517/156/5332/399694/3579/10681/2185/6349/6773/3265/3845/1794
hsa05142	Chagas disease	101	0.647314	1.744044	1.02×10^{-7}	9.67×10^{-7}	4.17×10^{-7}	7870	tags = 66%, list = 23%, signal = 51%	5522/5054/2769/3576/148022/355/714/7132/712/6348/713/5600/6352/4615/3458/8772/3553/3459/7040/2771/718/3569/10333/3827/6300/6347/4843/356/624/915/5331/3654/7046/2767/5595/2773/4790/1636/208/919/5515/5518/916/5291/3592/7099/917/841/5520/5290/3586/7048/5970/7124/5293/7042/8517/5332/8837/5521/5603/6349/5516/207/7043/1432/811

(Continued)

Table S2: *Continued*

ID	Description	setSize	enrichmentScore	NES	*p* value	*p*.adjust	*q* value	rank	leading_edge	core_enrichment
hsa04217	Necroptosis	157	0.600152	1.648721	1.37×10^{-7}	1.26×10^{-6}	5.43×10^{-7}	7633	tags = 57%, list = 22%, signal = 44%	3552/330/100526767/148022/29108/3013/355/283748/8329/6772/5836/197259/3012/7132/55506/637/7128/7416/8795/3458/8797/8334/5834/823/7098/8772/8743/55766/3553/3459/7417/824/2746/6776/92421/834/356/3014/25978/5119/92815/11035/3015/8605/8969/10616/329/3716/27243/128866/3320/5481/1536/5478/353376/123745/3718/51510/3717/7099/841/581/6778/192111/2495/8878/10059/7124/8717/57132/5610/81030/81858/51652/3326/8837/2747/91782/3146/9555/293/6773/3439/9525/818/124044/2512/79643/114548
hsa05022	Pathways of neurodegeneration - multiple diseases	475	0.49327	1.400077	1.72×10^{-7}	1.54×10^{-6}	6.65×10^{-7}	10941	tags = 62%, list = 32%, signal = 43%	22943/7476/125965/3552/10381/80326/7477/7277/2877/6804/84617/493869/1139/598/5582/1770/79861/355/8851/84790/7132/2861/637/9246/89780/5621/7416/5600/10133/2876/10376/823/578/8772/10452/3553/6712/1855/7417/5708/824/3798/50507/7316/5714/2906/5704/27019/4217/3569/4294/7133/6261/5879/6300/10105/283106/4843/356/55567/808/774/25978/1340/7384/5686/5578/1452/2535/5331/1454/5630/203068/4218/513/5691/10213/51806/10297/65018/1499/2878/5595/91860/11047/11211/836/518/2892/10383/1965/5604/54205/506/29982/60673/5606/4790/7332/1346/10010/7483/9377/7475/

(Continued)

Table S2: *Continued*

ID	Description	setSize	enrichmentScore	NES	*p* value	*p*.adjust	*q* value	rank	leading_edge	core_enrichment
										5605/4713/4710/5481/1536/5689/4709/5701/27089/7386/5695/5663/1329/4704/5707/64446/841/4726/10126/90550/10121/627/581/7318/5685/5713/5715/79139/4893/1020/27123/4747/1537/5970/8878/2915/7124/5690/7846/6389/8312/1435/10131/4711/840/5530/5705/10280/1857/517/842/7311/5694/5332/9776/5717/5709/8678/6233/5700/10476/488/5603/147700/1327/53349/10540/55062/1457/805/5861/374291/2911/5702/201625/293/84516/3265/7415/8324/8408/9001/3845/5688/51079/1347/5719/818/515/2882/509/351/516/3710/1349/9927/4724/27429/5718/55669/4702/6390/10120/5743/7472/4706/29110/126328/6622/4728/1432/30849/5693/55967/26100/10975/7317/56901/7388/5706/5609/5683/10671/4716/801/539/4717/81631/292/7326/5868/146754/7186/11273/8321/1616/4720/4725/27035/1459/4041/5682/9167/440738/1639/1460/7385/3309/64837/775/4696/80208/4700/9861/5710/4712/7979/55255/2932/5594/6392/5664/5534/1742/1350/11315/4719/440567/6263/3799/522/7480/1337/514/51465/4722/2903/11258/4715/7314/4509/5173/9896/4714/468/815/81029/7474/4723/5692/140775
hsa05145	Toxoplasmosis	109	0.628853	1.703822	3.12×10^{-7}	2.72×10^{-6}	1.17×10^{-6}	9206		

(Continued)

Table S2: *Continued*

ID	Description	setSize	enrichmentScore	NES	*p* value	*p*.adjust	*q* value	rank	leading_edge	core_enrichment
									tags = 72%, list = 27%, signal = 53%	3918/3914/3909/330/3655/598/3688/6772/3949/3912/7132/240/5600/79444/4615/3458/3310/958/3459/284217/7040/10319/2771/6300/10105/3312/4843/22798/3654/1234/5595/4793/3910/836/329/2773/54205/3716/5606/4790/208/3303/3588/3117/3911/3717/3592/7099/3111/3123/3122/841/3127/3586/5970/3112/7124/7042/8517/23118/3304/842/5603/3306/3113/23643/3115/207/7043/1432/146850/3109/8651/3915/6885/3913/3305/23533
hsa05205	Proteoglycans in cancer	203	0.565755	1.57656	3.49×10^{-7}	2.98×10^{-6}	1.28×10^{-6}	7958	tags = 55%, list = 23%, signal = 43%	7476/5328/4233/3673/5329/2318/80326/7291/7477/2817/3690/858/3678/1026/1277/857/1514/6237/5829/5582/4609/4318/355/7078/117581/3688/3693/3685/286/1839/2316/4313/1278/89780/5600/3091/7410/2335/595/60/7430/960/71/7074/7040/5879/6300/6608/6548/356/6385/3339/5578/2535/7057/6714/2247/8826/1499/5058/5595/11211/2317/836/5604/2064/3481/3316/4478/4060/208/387/7483/7409/7475/5605/10451/5291/3480/2885/7099/5962/5499/7422/5290/2017/5777/967/4893/7124/5293/7042/2002/1634/10855/9138/5603/3265/8324/3845/818/6382/7448/3710/4659/3549/5781/7472/207/6199/1432/2065
hsa05152	Tuberculosis	175	0.580289	1.605366	4.19×10^{-7}	3.47×10^{-6}	1.50×10^{-6}	8459	tags = 60%, list = 25%, signal = 45%	3552/3929/8877/51561/3606/3656/8767/6772/8915/2214/7132/637/929/5600/7421/

(Continued)

Table S2: *Continued*

ID	Description	setSize	enrichmentScore	NES	*p* value	*p*.adjust	*q* value	rank	leading_edge	core_enrichment
										4615/3458/8772/1594/2207/3553/3459/2209/26253/7040/22925/3689/1509/9902/718/2215/3684/30835/3569/10333/527/6300/64127/4843/4046/808/1051/3654/7879/6714/1263/51806/5595/91860/836/54205/3716/972/4790/7096/5878/208/10312/387/1520/2212/3588/3117/64581/11151/3717/3592/7099/3111/3123/3122/841/3127/581/3329/3586/1054/5970/3112/7124/3687/8717/7042/3916/5530/245972/842/5603/805/3113/3439/4360/818/3115/64170/2213/207/7043/1432/10332/3313/801/5868/5869/3109
hsa05162	Measles	139	0.605286	1.656028	4.40×10^{-7}	3.56×10^{-6}	1.54×10^{-6}	9281	tags = 63%, list = 27%, signal = 46%	3552/4938/898/4939/4940/598/4599/355/64135/1021/6772/637/7128/7161/4615/3310/578/8772/595/3560/3559/3553/3561/23586/30835/4600/3569/283106/3312/6776/356/915/3654/27113/9641/4793/836/1965/54205/3716/4478/4790/7187/208/3303/916/9367/1017/1019/5291/894/3718/3592/3665/7099/917/841/5290/581/440275/5970/8717/5293/5610/8517/23118/3304/842/3306/10399/1457/6773/8667/3439/3661/2213/207/29110/10332/896/868/1459/51284/1460/6885/3305/103
hsa04210	Apoptosis	136	0.606027	1.656473	5.09×10^{-7}	4.02×10^{-6}	1.73×10^{-6}	8046	tags = 62%, list = 23%, signal = 47%	1515/330/7277/5366/1514/3002/598/332/79861/355/1647/63970/84790/84823/597/4000/7132/637/10376/1508/8795/8797/823/

(Continued)

Table S2: *Continued*

ID	Description	setSize	enrichmentScore	NES	*p* value	*p*.adjust	*q* value	rank	leading_edge	core_enrichment
										578/8772/8743/1513/60/4616/71/824/6708/1521/1509/4217/356/143/10039/27113/5595/836/4001/329/1965/5604/54205/153090/4790/208/5551/1520/5605/8739/5291/1075/4170/841/5290/581/4893/1519/5970/7124/7846/8717/5293/8517/840/1439/842/6709/8837/839/3265/9020/3845/835/1522/3710/27429/207/56616/8737/7185
hsa04620	Toll-like receptor signaling pathway	102	0.637333	1.718162	6.97×10^{-7}	5.38×10^{-6}	2.32×10^{-6}	8119	tags = 60%, list = 24%, signal = 46%	3929/6373/3627/3576/148022/6696/6772/3663/4283/929/6348/5600/6352/4615/7098/6351/8772/1513/958/3553/3569/10333/5879/6300/9560/3654/9641/942/5595/5604/5606/4790/7096/7187/208/941/5605/353376/5291/3592/3665/7099/841/5290/51311/5970/7124/5293/8517/23118/5603/54472/6349/3439/23643/3661/207/29110/1432/8737/5609
hsa05140	Leishmaniasis	72	0.669168	1.761462	7.98×10^{-7}	5.88×10^{-6}	2.54×10^{-6}	7860	tags = 74%, list = 23%, signal = 57%	1917/3552/3688/6772/2214/4688/5600/4615/3458/1535/3553/3459/2209/7040/3689/718/2215/3684/6300/4843/3654/65108/5595/4793/3716/4790/4689/2212/1536/3117/3717/3592/7099/3111/3123/3122/653361/3127/5777/3586/5970/3112/7124/7042/2002/23118/5603/3113/1915/3115/5743/7043/1432
hsa04064	NF-kappa B signaling pathway	102	0.636109	1.714862	7.93×10^{-7}	5.88×10^{-6}	2.54×10^{-6}	9062	tags = 70%, list = 26%, signal = 51%	5328/3929/84433/330/6366/3576/29775/148022/598/6357/5971/1647/8915/597/4055/7132/79092/929/7128/8600/4615/

(Continued)

Table S2: *Continued*

ID	Description	setSize	enrichmentScore	NES	*p* value	*p*.adjust	*q* value	rank	leading_edge	core_enrichment
										4791/7412/6351/958/4616/3553/8740/2919/23586/4067/283106/4050/9560/10673/3932/3654/329/6363/4049/4790/7187/353376/7099/29760/8792/5970/7124/8717/2921/8517/23118/8837/1457/7329/9020/1896/23643/10913/7706/5743/8737/3383/7185/7186/7535/1459/23085/1460/6885/51588
hsa05100	Bacterial invasion of epithelial cells	77	0.660421	1.749415	8.74×10^{-7}	6.31×10^{-6}	2.72×10^{-6}	6271	tags = 61%, list = 18%, signal = 50%	4233/1212/858/3678/857/5829/9564/7414/3688/79658/10095/2335/60/23607/71/79767/10552/10109/999/391/25759/5879/23176/10096/10801/1495/6714/10093/10097/1499/3611/1759/6464/10094/387/81873/55752/5291/10163/10459/5290/2017/1399/10092/1785/5293/399694
hsa03008	Ribosome biogenesis in eukaryotes	103	0.626062	1.69	1.12×10^{-6}	7.94×10^{-6}	3.42×10^{-6}	10480	tags = 61%, list = 31%, signal = 43%	54913/56000/3692/55505/51068/283106/5901/10248/2091/28987/51119/6949/55341/23160/51077/27341/57455/10799/10940/54433/4809/10607/166378/55272/134430/25996/10775/55651/84128/1457/9790/29107/92856/84916/84135/10885/55813/65083/55781/10436/51602/22803/55127/1736/55226/1459/54464/26354/7514/10557/1460/10813/29102/10528/10556/51096/10199/102157402/138716/23560/10171/9724/79631
hsa04612	Antigen processing and presentation	69	0.671927	1.763251	1.61×10^{-6}	1.11×10^{-5}	4.80×10^{-6}	9175	tags = 81%, list = 27%, signal = 60%	3821/3135/6890/1514/3822/3805/6891/3134/3823/3106/3105/3107/1508/567/3458/3310/3824/925/3312/10197/6892/5721/

(Continued)

Table S2: *Continued*

ID	Description	setSize	enrichmentScore	NES	*p* value	*p*.adjust	*q* value	rank	leading_edge	core_enrichment
										3133/3308/972/3320/920/8302/3303/1520/3117/3111/926/5720/3123/3122/3127/3112/7124/3809/3804/3304/3326/3306/3113/3115/811/3811/2923/3812/3109/8625/4800/10437/3309/3305
hsa05206	MicroRNAs in cancer	266	0.5273	1.477906	1.94×10^{-6}	1.32×10^{-5}	5.68×10^{-6}	7066	tags = 31%, list = 21%, signal = 25%	5268/8091/1591/10642/5328/1943/4233/6624/54541/898/3690/3371/1946/3678/1026/85414/8626/5582/4609/4318/7078/9493/4854/5155/1021/995/113130/3162/993/89780/3667/6768/578/5292/5154/595/10298/7430/960/672/4082/6541/4363/5578/7057/7431/10297/5159/5595/836/5604/2064/6464/1870/4790/406992/1944/387/7168/5605/5291/894/2885/5962/4170/7422/5290/1399/4893/1869/407042/5293/7042/3066/399694/6935/3065/7329/407006/3265/5598/3845
hsa04145	Phagosome	147	0.585856	1.611333	2.36×10^{-6}	1.57×10^{-5}	6.75×10^{-6}	6189	tags = 54%, list = 18%, signal = 45%	3673/10381/7277/3690/84617/3135/6890/3678/1514/7058/79861/6891/3134/3688/3693/338382/3685/8685/84790/3106/2214/715/3105/949/929/4688/3107/10376/60/1535/71/4481/2209/4353/22925/3689/9902/718/2215/3684/30835/10333/527/5879/9146/4973/3133/7057/4074/203068/7879/7037/1781/29927/10383/9296/5878/4689/23480/529/10312/1520/2212/9341/1536/3117/64581/11151/7099/3111/3123/3122/

(Continued)

Table S2: *Continued*

ID	Description	setSize	enrichmentScore	NES	*p* value	*p*.adjust	*q* value	rank	leading_edge	core_enrichment
										653361/3127/53407/51382/3112/7846/3916/245972
hsa05418	Fluid shear stress and atherosclerosis	137	0.587553	1.605995	3.06×10^{-6}	1.99×10^{-5}	8.58×10^{-6}	8186	tags = 61%, list = 24%, signal = 46%	3552/7850/2817/3690/1906/858/5327/857/1514/119391/4318/59341/5155/445/5880/3685/9446/3162/4313/7132/7056/4688/5600/3458/7412/5154/60/1535/3553/71/1728/4217/5879/6300/6347/1843/658/6385/808/652/6714/6612/51806/4257/4258/1499/91860/7295/90/4790/3320/208/387/5563/2950/5291/7422/653361/5290/9817/5970/8878/7124/5293/8517/10365/3326/4780/5603/805/5607/5598/6382/25828/7341/207/4259/1432/3383/2944/5609/801/6613
hsa05010	Alzheimer disease	383	0.495759	1.40104	3.77×10^{-6}	2.41×10^{-5}	1.04×10^{-5}	10397	tags = 60%, list = 30%, signal = 43%	22943/7476/125965/3552/10381/80326/7477/7277/84617/1139/2597/79861/355/8851/23516/84790/25825/7132/637/89780/7416/3667/10376/823/8772/55851/3553/1855/7417/5708/824/3798/50507/5714/2906/201266/29986/5704/4217/3569/348/102/10105/283106/4843/808/1340/7384/5686/1452/2535/5331/1454/203068/513/5691/10213/51806/10297/1499/5595/91860/11047/11211/57142/836/518/10383/1965/5604/54205/506/29982/60673/27173/4790/6868/208/1346/7483/9377/7475/5605/4713/4710/5481/1536/5689/4709/5701/5291/27089/7386/5695/5663/1329/4704/5707/841/4726/90550/5290/5685/5713/5715/

(Continued)

Table S2: *Continued*

ID	Description	setSize	enrichmentScore	NES	*p* value	*p*.adjust	*q* value	rank	leading_edge	core_enrichment
										4893/1020/27123/1537/5970/2915/7124/5690/7846/6389/4311/8312/5293/5610/8517/1435/4711/840/5530/5705/1857/517/842/5694/5332/9776/5717/5709/8678/5700/10476/488/147700/1327/29985/55062/1457/805/374291/5702/293/3265/8324/8408/3845/5688/51079/1347/5719/55630/23385/515/509/351/516/3710/1349/4724/4035/5718/4702/6390/5743/7472/4706/207/126328/6622/4728/30849/5693/55967/26100/10975/56901/7388/5706/5609/7922/5683/4716/801/539/4717/292/7186/8321/51107/4720/4725/27035/1459/4041/57181/5682/9167/1460/7385/64837/775/91252/4696/4700/9861/5710/4712/7979/2932/5594/283375/6392/5664/5534/1350/4719/440567/6263/3799/522/7480/1337/514/4722/2903/221074/4715
hsa04512	ECM-receptor interaction	88	0.633975	1.699704	4.55×10^{-6}	2.85×10^{-5}	1.23×10^{-5}	4342	tags = 49%, list = 13%, signal = 43%	3918/3691/3914/3909/3673/3381/3675/3655/3696/3690/3371/1291/3694/1292/3678/1277/6696/3161/375790/7058/3688/3693/22801/3685/3912/1278/51206/1293/2335/1284/960/284217/10319/1282/6385/22798/3339/1605/7057/3695/3910/1288/3672
hsa05016	Huntington disease	305	0.509129	1.433183	4.94×10^{-6}	3.04×10^{-5}	1.31×10^{-5}	10922	tags = 63%, list = 32%, signal = 43%	5468/125965/10381/7277/1212/2877/6804/84617/493869/7052/1770/79861/84790/1173/6648/7416/2876/10376/1175/7417/

(Continued)

Table S2: *Continued*

ID	Description	setSize	enrichmentScore	NES	*p* value	*p*.adjust	*q* value	rank	leading_edge	core_enrichment
										5708/160/3798/5714/5704/27019/4217/84699/4294/10105/55567/774/1340/7384/5686/5331/203068/27113/163/513/5691/6507/10213/2878/11047/836/518/2892/10383/54205/506/29982/60673/1346/9377/4713/4710/5689/4709/5701/27089/7386/5695/1329/4704/5707/64446/6667/841/9586/4726/10126/10121/627/581/5438/5685/5713/5715/1537/2915/5690/7846/6389/4711/5705/517/842/3066/10488/5434/5694/5430/5332/9776/5717/5709/8678/5700/10476/5437/147700/1327/10540/55062/3065/374291/5702/201625/293/84516/8408/9001/5688/51079/1347/5719/515/5431/2882/23186/509/5978/516/1349/4724/5441/5718/4702/6390/10120/4706/126328/4728/30849/5439/5693/55967/26100/10975/56901/7388/5706/5609/5683/10671/7019/4716/539/4717/292/146754/7186/4720/4725/1211/5682/9167/1639/7385/64837/4696/4700/9861/5710/4712/7979/25942/6392/90993/1742/5440/1350/4719/440567/6908/3799/522/7157/1337/514/8218/4722/11258/4715/2033/4509/4714/1213/6875/4723/5692
hsa04115	p53 signaling pathway	73	0.657017	1.734645	5.46×10^{-6}	3.30×10^{-5}	1.42×10^{-5}	5841	tags = 55%, list = 17%, signal = 46%	5268/2810/64065/5054/898/5366/6241/3486/1026/598/9133/355/891/1647/1021/51512/3732/63970/55240/9540/983/637/

(Continued)

Table S2: *Continued*

ID	Description	setSize	enrichmentScore	NES	*p* value	*p*.adjust	*q* value	rank	leading_edge	core_enrichment
										7161/8795/8797/595/51246/4616/1111/901/7057/27113/836/54205/1017/1019/894/841/581/84883
hsa04933	AGE-RAGE signaling pathway in diabetic complications	100	0.614642	1.654015	6.10×10^{-6}	3.62×10^{-5}	1.56×10^{-5}	7923	tags = 64%, list = 23%, signal = 49%	113026/3552/5054/2152/1906/7424/3576/1277/1281/6772/4088/4313/1278/7056/5600/2335/7412/5292/595/1284/3553/7040/50507/3569/5879/6300/6347/1282/6776/1729/5578/5331/7046/5333/5595/836/1288/1958/4790/208/2308/1536/1019/5291/3717/7422/5290/581/4893/7048/5970/7124/4772/5293/7042/1287/5332/5603/3265/3845/207/7043/1432/3383
hsa05020	Prion disease	273	0.51567	1.445754	7.99×10^{-6}	4.65×10^{-5}	2.01×10^{-5}	11518	tags = 69%, list = 34%, signal = 46%	125965/3552/10381/116444/7277/858/84617/857/79861/5880/84790/714/735/712/5621/7416/713/4688/5600/6352/10376/3310/1535/3553/7417/5708/3798/5714/2906/5704/3569/84699/6261/5879/6300/10105/283106/3312/774/1340/7384/5686/203068/513/5691/10213/5595/11047/836/518/10383/1958/1965/54205/506/4689/1346/3303/9377/4713/4710/1536/5689/4709/5701/5291/27089/7386/5695/1329/4704/5707/9586/4726/653361/90550/5290/581/5685/5713/5715/1537/7124/5690/7846/6389/5293/4711/5530/5705/3304/517/842/10488/5694/5717/5709/5700/10476/5603/147700/1327/3306/1457/374291/5702/293/4685/5688/51079/1347/5719/515/509/

(Continued)

Table S2: *Continued*

ID	Description	setSize	enrichmentScore	NES	*p* value	*p*.adjust	*q* value	rank	leading_edge	core_enrichment
										116443/516/3710/1349/4724/5718/4702/6390/4706/126328/4728/1432/5693/55967/10975/56901/7388/5706/5683/4716/539/4717/292/3915/4720/4725/1459/5682/9167/5566/1460/7385/3309/64837/775/3305/4696/4700/9861/5710/4712/7979/2932/5594/6392/5534/90993/1350/4719/440567/6263/3799/522/5567/1337/514/4722/2903/4715/4509/4714/468/4723/5692/4851/143471/4695/1388/5580/10963/4708/7419/4694/5296/89953
hsa05222	Small cell lung cancer	92	0.621027	1.666817	8.31×10^{-6}	4.68×10^{-5}	2.02×10^{-5}	6551	tags = 60%, list = 19%, signal = 48%	3918/3914/3909/3673/330/3675/3655/898/1026/598/4609/1647/1021/3688/3685/3912/79444/2335/578/595/1284/4616/284217/10319/1164/1282/4843/22798/3910/5915/836/1288/329/54205/1870/1163/4790/7187/208/1017/1019/3911/5291/5290/581/5970/1869/5293/8517/1287/842/6256/4149/5925/1030
hsa04670	Leukocyte transendothelial migration	114	0.593275	1.606754	8.25×10^{-6}	4.68×10^{-5}	2.02×10^{-5}	6009	tags = 48%, list = 18%, signal = 40%	9076/9071/5829/5582/4318/9564/7408/81/7414/87/3688/10686/5880/4313/4688/5600/23562/7410/10627/7412/7070/10398/60/7430/1535/71/3689/4267/2771/7122/3684/5879/6300/103910/1495/1364/5578/9080/1499/9074/2773/5908/1366/1500/4478/4689/387/7409/1536/10451/5291/653361/5290/6494/5293
hsa04530	Tight junction	169	0.544616	1.505747	1.05×10^{-5}	5.82×10^{-5}	2.51×10^{-5}	7294		

(Continued)

Table S2: *Continued*

ID	Description	setSize	enrichmentScore	NES	*p* value	*p*.adjust	*q* value	rank	leading_edge	core_enrichment
									tags = 49%, list = 21%, signal = 38%	5522/9076/7277/9071/79861/7408/81/87/3688/10686/84790/10095/4734/79778/10376/23562/10627/91862/595/4627/10398/60/7430/71/10552/7074/9368/8531/23370/79784/7122/10109/4217/5879/27134/10096/103910/1364/9080/4637/51421/11346/6714/10093/4218/3996/10097/9074/57530/2064/3308/1366/7082/10094/4478/387/5515/5518/5563/154810/81873/1019/7454/4629/5111/5962/56288/51762/11149/5520/2017/10092/3993/7846/8189/5521/9414/92359/57826/6794/123720/5516
hsa05014	Amyotrophic lateral sclerosis	364	0.492253	1.389119	1.22×10^{-5}	6.62×10^{-5}	2.85×10^{-5}	11812	tags = 66%, list = 34%, signal = 44%	125965/10381/7277/2877/84617/493869/598/1770/79861/84790/7132/637/56000/7416/5600/10133/2876/10376/10452/60/71/5708/3798/5714/2906/5704/27019/311/4217/7133/5879/6300/4843/834/3084/55567/10189/25978/1340/7384/5686/23636/5630/203068/5216/4218/513/5691/10213/65018/2878/11047/79023/836/518/10383/55706/1965/54205/506/29982/60673/400916/5606/1346/10010/9377/4713/4710/53371/5689/4709/5701/27089/7386/5695/1329/4704/5707/64446/4726/10126/90550/10121/581/5685/5713/5715/10718/79139/10762/4747/1537/8878/7124/5690/7846/6389/6396/4711/5530/5705/10280/29979/517/842/5694/9776/5717/5709/8678/5700/

(Continued)

Table S2: *Continued*

ID	Description	setSize	enrichmentScore	NES	*p* value	*p*.adjust	*q* value	rank	leading_edge	core_enrichment
										10476/5217/5603/147700/1327/10540/310/55062/29978/5861/374291/5702/201625/84516/29107/7415/8408/9001/9688/5688/51079/1347/5719/515/2882/509/516/3710/1349/4724/5718/4702/6390/10120/94026/4706/29110/4686/126328/4728/1432/30849/5693/55967/26100/10975/56901/7388/5706/23165/5683/10671/4716/539/4717/81631/5868/146754/7186/11273/57122/1616/4720/9542/4725/5682/9167/440738/1639/7385/3309/64837/4696/80208/4700/9861/9883/145957/5710/4712/7979/55255/9631/6392/5534/1350/4719/440567/3799/522/7157/1337/514/4722/2903/11258/4715/8480/79902/4509/9896/4714/468/100101267/4928/9972/4723/5692/140775/23511/56171/6428/143471/23064/4695/4708/23225/8021/4744/4694/3178/89953/5684/375189/348995/3181/1345/498
hsa04630	JAK-STAT signaling pathway	166	0.556786	1.537648	1.53×10^{-5}	8.17×10^{-5}	3.52×10^{-5}	6205	tags = 41%, list = 18%, signal = 34%	53833/58985/338376/64109/3589/133396/23529/51561/53832/1026/3601/598/4609/5155/1438/3574/6772/3566/9180/3458/5292/5154/595/3560/3559/3459/3561/9466/9021/3600/3569/282617/85480/246778/6776/3595/1437/3594/50615/29949/3952/5159/116379/282616/282618/3716/3976/5008/208/3588/5291/3597/894/3718/

(Continued)

Table S2: *Continued*

ID	Description	setSize	enrichmentScore	NES	*p* value	*p*.adjust	*q* value	rank	leading_edge	core_enrichment
										3717/3592/2885/8027/4170/3581/5290/5777/6778/3586/3575/5293/1439/9306
hsa05415	Diabetic cardiomyopathy	203	0.533138	1.485667	1.55×10^{-5}	8.17×10^{-5}	3.52×10^{-5}	11979	tags = 68%, list = 35%, signal = 45%	6513/125965/1277/2597/5582/9945/4318/1281/5880/7137/4088/4313/1278/7416/4688/3667/5600/1535/7417/7040/1509/5879/6300/10105/5524/1340/7384/5578/5331/7046/513/518/506/2539/4790/1636/4689/208/1346/9377/5350/4713/4710/1536/2997/4709/5291/27089/7386/1329/4704/5499/6667/4726/653361/5290/7048/1537/5970/6389/5293/7042/4711/517/5332/10476/488/5603/1327/374291/293/51079/1347/818/515/509/2998/516/1349/4724/4702/6390/4706/207/7043/126328/2936/4728/1432/55967/10975/56901/7388/4716/539/4717/292/4720/4725/2673/9167/9882/7385/4696/4700/5500/5972/5501/4846/5728/4712/2932/6392/1350/4719/440567/522/1337/514/4722/4715/5465/4509/4714/815/5165/4723/4695/5580/4708/7419/4694/5296/5162/1345/498/29796/5601/1351
hsa04625	C-type lectin receptor signaling pathway	104	0.601201	1.623307	1.58×10^{-5}	8.20×10^{-5}	3.53×10^{-5}	8191	tags = 62%, list = 24%, signal = 48%	51561/6237/6367/29108/5971/6772/3659/8915/5600/4791/2207/93978/3553/26253/30835/3569/6300/4046/834/602/808/1960/6714/1263/9641/338339/51806/5058/5595/91860/1959/4790/208/387/64581/5291/6361/3592/841/5290/4893/3586/5970/7124/4772/5293/8517/5530/5603/805/9261/6773/

(Continued)

Table S2: *Continued*

ID	Description	setSize	enrichmentScore	NES	*p* value	*p*.adjust	*q* value	rank	leading_edge	core_enrichment
										3265/9020/3845/64170/3710/114548/5743/5781/207/1432/10332/801/4773
hsa04915	Estrogen signaling pathway	137	0.570477	1.55932	1.72×10^{-5}	8.81×10^{-5}	3.80×10^{-5}	7066	tags = 43%, list = 21%, signal = 34%	3868/3872/7031/3861/25984/3880/3860/3866/7039/54474/3859/3875/4318/1839/2852/4313/2288/2289/3857/3310/3885/1509/2771/84699/25759/3312/808/5331/109/6714/51806/5595/91860/2773/5604/6464/3320/208/3303/5605/5291/2885/6667/9586/5290/4893/5293/3304/10488/5332/399694/3326/3306/805/2911/3265/3858/8202/3845
hsa04932	Non-alcoholic fatty liver disease	155	0.554939	1.525228	1.92×10^{-5}	9.67×10^{-5}	4.17×10^{-5}	11979	tags = 72%, list = 35%, signal = 47%	5468/125965/3552/3576/355/7132/637/3667/5600/3553/7040/9021/4217/3569/5879/6300/356/1340/7384/79602/3952/2931/836/1965/54205/4790/208/1346/9377/4713/4710/5563/4709/5291/27089/7386/1329/4296/4704/841/4726/5290/581/1537/5970/7124/6389/5293/4711/840/6256/5603/1327/83737/374291/6945/51079/1347/1349/4724/6720/4702/6390/4706/207/126328/4728/1432/5571/55967/10975/56901/7388/4716/4717/7186/4720/4725/9167/10018/7385/51094/4696/4700/4712/51085/2932/6392/1350/4719/440567/998/1337/4722/4715/5465/4714/468/10062/4723/5562/4695/4708/3725/4694/5296/22877/1345/29796/5601/1351
hsa05133	Pertussis	76	0.625481	1.654473	2.01×10^{-5}	9.94×10^{-5}	4.29×10^{-5}	7860		

(Continued)

Table S2: *Continued*

ID	Description	setSize	enrichmentScore	NES	*p* value	*p*.adjust	*q* value	rank	leading_edge	core_enrichment
									tags = 68%, list = 23%, signal = 53%	3552/6374/6372/51561/3678/3576/148022/29108/3688/714/3659/715/712/929/713/5600/4615/716/710/3553/1072/3689/2771/718/3684/3569/6300/4843/834/808/3654/51806/5595/91860/836/2773/4790/387/353376/3592/7099/3586/5970/7124/840/5603/805/23643/3661/3394/114548/1432
hsa04666	Fc gamma R-mediated phagocytosis	96	0.597715	1.607291	2.09×10^{-5}	0.000102	4.40×10^{-5}	6009	tags = 54%, list = 18%, signal = 45%	8877/8853/5582/7408/283748/5880/2214/10095/7410/2934/3984/10552/2209/50807/1072/4082/2215/10109/3055/5879/4067/5337/10096/5578/8605/10093/382/10097/65108/5058/5595/23396/5604/274/10094/208/2212/7409/3985/81873/8612/10451/5291/7454/123745/10163/653361/5290/1399/10092/1785/5293
hsa04520	Adherens junction	71	0.628138	1.65427	3.46×10^{-5}	0.000164	7.08×10^{-5}	9016	tags = 70%, list = 26%, signal = 52%	4233/5818/6591/4008/81/7414/87/5880/6615/4088/81607/5819/60/71/10458/999/5879/283106/1495/7046/6714/6932/8826/1499/5595/5792/2064/7082/1500/5795/387/7454/10163/3480/5797/56288/5777/7048/117178/8976/1457/2241/7525/1956/1459/6934/9855/5770/1460/6885
hsa05322	Systemic lupus erythematosus	127	0.564185	1.533773	3.42×10^{-5}	0.000164	7.08×10^{-5}	7204	tags = 49%, list = 21%, signal = 39%	3013/81/87/8329/714/2214/8351/3012/715/55506/735/8347/712/713/8339/3458/8334/716/55766/8345/958/2209/8970/8357/718/2215/8343/128312/6737/3014/85236/92815/3015/3017/8969/8370/942/8355/8353/8348/6628/941/3021/2212/3117/440689/

(Continued)

Table S2: *Continued*

ID	Description	setSize	enrichmentScore	NES	*p* value	*p*.adjust	*q* value	rank	leading_edge	core_enrichment
										3111/3123/3122/8349/3127/3586/6632/3112/7124/8362/9555/440093/3113/8294/8367/3115
hsa05323	Rheumatoid arthritis	88	0.612926	1.643271	3.63×10^{-5}	0.00017	7.33×10^{-5}	7923	tags = 66%, list = 23%, signal = 51%	4312/3552/6374/6372/4314/3589/51561/3606/3576/1514/6364/8600/6348/6352/3458/1513/3553/7040/2919/3689/3600/3569/527/6347/4050/10673/1437/942/9296/8741/529/941/10312/3117/7099/3111/3123/3122/7422/3127/51382/8792/3112/7124/2921/7042/1435/245972/54/6349/3113/8992/3115/1493/2321/526/7043/3383
hsa05134	Legionellosis	57	0.664043	1.721048	3.90×10^{-5}	0.00018	7.75×10^{-5}	6982	tags = 65%, list = 20%, signal = 52%	1917/3606/3576/29108/929/4615/3310/4791/3553/2919/3689/718/3684/3569/3312/834/23786/836/54205/3297/4790/3303/3592/81876/7099/841/3329/5970/7124/2921/840/3304/842/3306/5861/7415/1915
hsa05012	Parkinson disease	266	0.499526	1.400063	6.04×10^{-5}	0.000274	0.000118	11979	tags = 71%, list = 35%, signal = 46%	125965/10381/7054/7277/84617/598/79861/23516/84790/2861/9246/7416/10376/7417/5708/3798/7316/5714/201266/2771/29986/5704/4217/10105/1843/808/1340/7384/5686/203068/513/5691/10213/51806/65018/91860/11047/7295/836/518/10383/2773/1965/54205/506/27173/7332/1346/9377/4713/4710/1813/5689/4709/5701/27089/7386/5695/1329/4704/5707/4726/90550/581/9817/7318/5685/5713/5715/1537/5690/7846/6389/10131/4711/5705/517/842/7311/5694/5717/5709/6233/5700/10476/4780/

(Continued)

Table S2: *Continued*

ID	Description	setSize	enrichmentScore	NES	*p* value	*p*.adjust	*q* value	rank	leading_edge	core_enrichment
										147700/1327/29985/805/374291/5702/293/5688/51079/1347/5719/818/55630/515/509/516/3710/25828/1349/9927/4724/27429/5718/55669/4702/6390/4706/126328/6622/4728/5693/55967/10975/7317/56901/7388/5706/7922/5683/4716/801/539/4717/292/7326/1616/4720/4725/57181/5682/9167/5566/7385/3309/64837/91252/4696/4700/9861/5710/4712/7979/283375/6392/1350/11315/4719/440567/6263/3799/522/5567/7157/1337/514/51465/4722/221074/4715/55334/7314/4509/4714/468/815/4723/5692/2770/143471/4695/4708/7419/4694/89953/5684/4129/5413/1345/498/29796/5601/1351
hsa01232	Nucleotide metabolism	85	0.609287	1.628101	6.53×10^{-5}	0.000293	0.000126	9753	tags = 71%, list = 28%, signal = 51%	978/9615/7498/205/4907/954/7083/6241/129607/1890/7371/100/7378/124583/284958/4860/144811/4831/5167/654364/7298/122622/377841/272/353/4830/1841/2987/4833/1635/51251/3614/158067/1854/8833/3251/7084/22978/26289/30833/1716/159/79077/8623/51727/6240/955/51020/271/132/1503/3704/3615/10201/957/102157402/158/953/29922/131870
hsa04010	MAPK signaling pathway	294	0.491016	1.379983	7.39×10^{-5}	0.000327	0.000141	8615	tags = 52%, list = 25%, signal = 39%	2069/374/1943/3552/4233/1969/2318/27092/7039/59285/5801/9965/2250/1847/1946/7424/6237/4909/5582/4609/355/5155/5971/1647/283748/5880/1852/8986/2316/1849/7132/929/9448/5600/59283/4615/

(Continued)

Table S2: *Continued*

ID	Description	setSize	enrichmentScore	NES	*p* value	*p*.adjust	*q* value	rank	leading_edge	core_enrichment
										3310/4791/777/5154/55970/4616/3553/1846/7040/786/4217/5062/2768/5879/6300/1843/3312/356/774/1436/5578/3654/7046/9064/8605/5159/2247/5058/5595/2317/51776/1845/836/7867/5604/5908/2064/5606/10125/3481/1844/4790/3315/1944/208/56034/3303/5605/3556/2246/2872/6722/5228/51378/123745/8912/1848/93589/3480/2885/4296/773/7422/4908/627/1399/5921/4893/7048/5970/5778/7124/8717/2324/4772/51347/7042/8517/1435/2002/5530/23118/3304/115727/4149/5603/10368/3306/27330/9261/2252/3265/9020/59284/5607/5598/3845/8491/51295/2321/5156/8074/207/7043/5924/1432/2065/2323/5609/5922/1398/7186/1956/2261/1616/5536
hsa04141	Protein processing in endoplasmic reticulum	170	0.54194	1.499078	7.75×10^{-5}	0.000339	0.000146	11966	tags = 73%, list = 35%, signal = 48%	30001/26232/267/3310/823/578/3337/824/4217/3312/23640/10970/26270/10273/5886/29927/1965/7466/10484/23480/3320/7321/3303/10134/7353/5887/80700/23645/3300/54788/51726/6747/6184/5034/11253/581/440275/79139/55741/64215/5610/9532/6396/29979/3304/10960/1603/3326/4780/10808/3301/3306/29978/10802/7323/55968/10427/7415/55829/6500/10130/22824/54431/51360/1650/9871/5589/56681/8454/9632/51035/811/10952/5611/5609/

(Continued)

Table S2: *Continued*

ID	Description	setSize	enrichmentScore	NES	*p* value	*p*.adjust	*q* value	rank	leading_edge	core_enrichment
										2923/22872/573/9978/7326/10294/7993/7186/7841/91445/10525/56886/55757/55666/3309/9601/7322/6238/10113/3305/3703/81567/133022/821/10956/91319/23190/51465/165324/80267/27102/9373/10483/468/1410/85479/7095/3998/23193/64374/1388/6185/7184/9695/80331/201595/6745/55768/5601
hsa05321	Inflammatory bowel disease	62	0.640451	1.674555	9.08×10^{-5}	0.000391	0.000169	7204	tags = 63%, list = 21%, signal = 50%	3552/51561/3606/6772/4088/3566/8809/3458/2625/3553/3459/3561/7040/3569/50943/64127/30009/3595/3594/50615/4790/3117/3592/7099/3111/3123/3122/8807/3127/6778/3586/5970/3112/7124/4772/7042/4094/3113/3115
hsa05235	PD-L1 expression and PD-1 checkpoint pathway in cancer	89	0.599887	1.608516	9.37×10^{-5}	0.000399	0.000172	9846	tags = 66%, list = 29%, signal = 47%	116071/29126/148022/6772/5133/10538/55509/5600/3091/4615/3458/3459/4794/6300/283106/915/3932/5595/4793/5604/3716/5606/10125/4790/920/208/919/5605/916/353376/5291/3717/7099/917/5290/5777/4893/5970/4772/5293/8517/5530/5603/1457/3265/3845/5781/207/6199/1432/4773/1956/7535/1459/1460/5728/5594/5534/3460
hsa05208	Chemical carcinogenesis - reactive oxygen species	222	0.514066	1.434172	9.99×10^{-5}	0.00042	0.000181	12747	tags = 72%, list = 37%, signal = 46%	253152/125965/4233/119391/79852/9446/3162/1572/6648/7416/4688/5600/3091/1535/7417/50507/1109/1728/4217/1646/4025/5879/6300/10105/5337/1340/7384/6714/513/4257/4258/5595/518/25865/5604/

(Continued)

Table S2: *Continued*

ID	Description	setSize	enrichmentScore	NES	*p* value	*p*.adjust	*q* value	rank	leading_edge	core_enrichment
										196/506/4790/5795/208/1346/9377/5605/4713/4710/4709/5291/27089/7386/2885/1329/4704/4726/7422/653361/5290/9817/4893/1537/5970/6389/5293/8517/4711/517/10476/4780/5603/1327/374291/293/3265/9020/3845/51079/1347/65010/515/509/516/1349/4724/4702/6390/5781/4706/207/126328/4259/4728/1432/55967/10975/56901/2944/7388/5609/25/4716/539/4717/292/5338/1956/4720/4725/27035/9167/5770/10327/7385/4696/4700/5728/4712/1545/2309/5594/52/6392/1350/1836/4719/440567/27/522/1337/8644/514/2949/4722/4715/6416/4509/4714/4723/1147/4695/5580/4708/3725/7419/4694/5296/1345/498/369/29796/5601/1351/4705/1645/4508/4513/6654/572/6647/23683/4697/84701
hsa04120	Ubiquitin mediated proteolysis	142	0.54176	1.482922	0.000102	0.000425	0.000183	10795	tags = 66%, list = 31%, signal = 46%	330/23624/5371/11065/26232/991/4734/9246/79444/27338/672/57154/7316/9021/10477/6923/65264/55236/997/10273/10054/51343/329/9616/7332/140739/7321/4281/7325/996/9040/7328/9690/9817/7318/9320/92912/7311/7324/6233/10055/7320/83737/27339/7329/7323/6921/3093/9039/1643/64682/6500/8451/54926/8450/51529/11059/26272/8881/7337/8454/7317/22954/25847/9978/7326/134111/8651/8945/8453/868/8924/8916/9820/10393/7322/51588/

(Continued)

Table S2: *Continued*

ID	Description	setSize	enrichmentScore	NES	*p* value	*p*.adjust	*q* value	rank	leading_edge	core_enrichment
										7319/7334/89910/6502/246184/26091/55120/1642/23291/1161/4193/51465/7314/22888/8452/867/51433
hsa05332	Graft-versus-host disease	37	0.696582	1.704836	0.000105	0.00043	0.000185	8459	tags = 84%, list = 25%, signal = 63%	3552/3821/3135/3002/355/3134/3106/3105/3107/3458/3824/3553/3569/356/3133/942/941/5551/3117/3111/3123/3122/3127/3112/7124/3804/3113/3115/3811/3812/3109
hsa04360	Axon guidance	181	0.524279	1.453401	0.000122	0.000492	0.000212	7714	tags = 47%, list = 22%, signal = 37%	25791/7869/1943/4233/8482/1969/3897/655/29984/22885/2041/10509/1947/1946/10512/2048/10371/6237/57715/2051/1948/90249/64101/3688/5880/5361/5365/23654/10501/219699/59277/3984/10627/10398/10298/22854/1072/4690/2050/2771/54961/57522/5062/5879/6608/658/103910/5578/6405/64221/6714/5058/5595/2773/3611/1944/387/9423/54910/3985/54434/5291/56288/223117/5998/5290/8440/5921/56924/4893/1020/5293/5530/91584/3983/3265/57556/3845/6091/818/55558/7223/54437/8633/5781
hsa05202	Transcriptional misregulation in cancer	190	0.524869	1.459677	0.000137	0.000549	0.000237	7370	tags = 45%, list = 21%, signal = 36%	8091/5328/5468/4233/7850/3207/330/4314/8900/3206/5327/5371/3576/3486/1026/3195/85414/3002/598/890/4609/4318/6692/51513/1647/3728/597/3205/8351/7490/929/578/5154/7849/3560/958/4616/5218/2209/4353/4286/8357/3684/3569/3248/6688/64919/2138/1051/1436/1437/942/3695/2892/329/8355/8353/2530/

(Continued)

Table S2: *Continued*

ID	Description	setSize	enrichmentScore	NES	*p* value	*p*.adjust	*q* value	rank	leading_edge	core_enrichment
										4790/3021/2308/894/1848/6929/3480/8148/6667/581/7048/5970/4086/171558/3066/51274/6935/6256/4149/4094/3065/440093/7030/2120/1643/1053/4005/2321
hsa03050	Proteasome	46	0.668956	1.692425	0.000142	0.000549	0.000237	9821	tags = 87%, list = 29%, signal = 62%	5698/5696/5699/3458/5708/5714/5704/10197/51371/5686/5721/5691/10213/11047/5689/5701/5695/5720/5707/5685/5713/5715/5690/5705/5694/5717/5709/5700/5702/5688/5719/5718/5693/5706/5683/5682/9861/5710/7979/9491
hsa04659	Th17 cell differentiation	105	0.581042	1.568867	0.00014	0.000549	0.000237	6503	tags = 53%, list = 19%, signal = 43%	51561/6772/4088/3566/5600/3091/3458/2625/10148/3560/3559/3553/3459/3561/9466/7040/4794/3569/50943/6300/246778/6776/30009/915/3932/7046/3594/50615/5595/4793/196/3716/4790/3320/920/919/916/3556/3117/3718/3717/3111/3123/917/3122/3127/6778/7048/5970/3112/4772/8517/5530/6256/3326/5603
hsa04514	Cell adhesion molecules	154	0.53299	1.465645	0.000142	0.000549	0.000237	7325	tags = 51%, list = 21%, signal = 40%	1001/3897/9076/84189/79679/3655/9071/3696/3135/29126/5818/64101/3134/3688/10686/3685/5133/3106/80381/80380/5817/152404/1462/5819/3105/3107/23562/7412/958/22854/6614/965/6402/3689/4267/7122/925/6404/3684/999/201633/64115/6385/9379/5802/1364/9080/3133/942/3695/5792/9074/29851/1366/920/941/9672/26047/3117/914/5797/57502/3111/926/3123/3122/3127/3112/22829/923/2734/6693/3384/4685/3113/3115/6382/1493

(Continued)

Table S2: *Continued*

ID	Description	setSize	enrichmentScore	NES	*p* value	*p*.adjust	*q* value	rank	leading_edge	core_enrichment
hsa04940	Type I diabetes mellitus	40	0.6906	1.708478	0.000145	0.000553	0.000239	7558	tags = 75%, list = 22%, signal = 59%	3552/5798/3135/3002/355/3134/3106/3105/3107/3458/3553/356/3133/942/2571/4049/941/5551/3117/3592/3111/3123/3122/3127/3329/3112/7124/3113/3115/1363
hsa04660	T cell receptor signaling pathway	103	0.573158	1.547191	0.000147	0.000553	0.000239	9803	tags = 64%, list = 29%, signal = 46%	84433/5133/8915/5600/3458/7410/10298/4794/4690/925/5062/6300/915/3932/1437/5058/5595/4793/5604/29851/10125/4790/920/208/919/387/7409/5605/916/10451/1019/5291/2885/926/3937/917/5290/8440/56924/5777/4893/3586/5970/7124/4772/5293/8517/5530/5603/3265/9020/3845/1493/207/1432/5609/4773/7535/868/6885/3702/57144/5788/2932/5594/5534
hsa05330	Allograft rejection	34	0.687423	1.666977	0.000183	0.000677	0.000292	7204	tags = 74%, list = 21%, signal = 58%	3135/3002/355/3134/3106/3105/3107/3458/958/356/3133/942/941/5551/3117/3592/3111/3123/3122/3127/3586/3112/7124/3113/3115
hsa04640	Hematopoietic cell lineage	95	0.582778	1.565956	0.000181	0.000677	0.000292	6047	tags = 47%, list = 18%, signal = 39%	3552/7850/3673/3675/3589/3655/3690/3678/1438/3574/3566/924/929/3559/960/3553/2209/925/3684/3569/915/1436/966/1437/7037/952/3672/920/916/290/3117/914/3111/926/3123/928/917/3122/3581/3127/3112/7124/4311/3575/1435
hsa05412	Arrhythmogenic right ventricular cardiomyopathy	77	0.595696	1.577964	0.000221	0.000807	0.000348	5331	tags = 47%, list = 16%, signal = 40%	3691/5318/3673/27092/59285/1829/3675/3655/3696/3690/1824/3694/3678/1832/3688/3693/22801/3728/3685/4000/2697/59283/60/71/284217/786/1495/1605/6932/1499/3695/6444/3672/6443/93589/6442

(Continued)

Table S2: *Continued*

ID	Description	setSize	enrichmentScore	NES	*p* value	*p*.adjust	*q* value	rank	leading_edge	core_enrichment
hsa04623	Cytosolic DNA-sensing pathway	74	0.604084	1.596324	0.000224	0.000808	0.000348	7897	tags = 51%, list = 23%, signal = 40%	9447/1687/3606/3627/29108/197259/6352/6351/8772/4210/3553/23586/3569/834/9560/115004/11035/9641/4793/836/25939/4790/3665/841/5970/8517/840/5434/81030/5437/340061/3439/79792/3661/5441/114548/29110/8737
hsa04610	Complement and coagulation cascades	86	0.584032	1.558127	0.000232	0.000829	0.000357	4824	tags = 47%, list = 14%, signal = 40%	5104/5328/5055/5329/2153/5265/5054/2161/2152/5327/629/2147/10544/714/715/735/712/7056/713/716/5270/2151/2162/710/11326/728/3689/7035/3075/719/718/3684/3827/624/966/9002/623/2266/2243/2149
hsa05220	Chronic myeloid leukemia	76	0.594408	1.572281	0.000238	0.00084	0.000362	10158	tags = 72%, list = 30%, signal = 51%	1026/598/4609/1647/1021/4088/578/595/4616/7040/25759/6776/7046/5595/5604/6464/1870/4790/208/5605/1019/5291/613/2885/5290/581/1399/4893/7048/5970/1869/1488/5293/7042/8517/3066/399694/5925/3065/3265/3845/1643/5781/207/7043/25/1398/1487/1029/5594/1871/861/4193/1027/7157
hsa04611	Platelet activation	124	0.549178	1.493609	0.000273	0.000955	0.000412	9649	tags = 60%, list = 28%, signal = 43%	3673/3690/1277/7408/1281/2147/3688/283748/5593/1278/5600/51206/6915/10627/60/2207/71/2771/4638/6300/4067/103910/83706/5331/109/6714/8605/5742/9002/5595/8773/2773/54518/5908/5028/10125/2266/2243/208/5739/387/2212/2149/5592/5291/123745/3937/5499/5290/5293/5332/84876/10672/9138/5603/7094/6786/3710/8673/4659/207/1432/146850/85366/

(Continued)

Table S2: *Continued*

ID	Description	setSize	enrichmentScore	NES	*p* value	*p*.adjust	*q* value	rank	leading_edge	core_enrichment
										196883/64805/5566/23533/5500/5501/4846/6850/83660/5594
hsa03250	Viral life cycle – HIV-1	62	0.621478	1.624948	0.000291	0.001006	0.000434	11256	tags = 77%, list = 33%, signal = 52%	200315/4599/27350/9582/636/60489/140564/5045/4600/5901/200316/55201/9146/6829/684/1234/9638/25939/128866/10015/23534/920/164668/5478/25920/7936/4131/8178/10955/6598/7469/6827/9525/79643/7251/7852/1025/7514/5300/80237/904/27125/2033/22936/9972/85363/23299/27183
hsa04068	FoxO signaling pathway	129	0.538092	1.464702	0.000309	0.001059	0.000457	10491	tags = 62%, list = 31%, signal = 43%	114907/5347/1026/9133/891/1647/9454/4088/6648/3667/5600/85417/5896/10769/8743/595/4616/7040/6446/3569/6300/901/356/10733/7046/1454/1263/10110/5595/5604/8698/208/3276/2308/5605/5563/9455/1017/23710/5291/894/116986/3480/2885/5290/80854/4893/3586/7048/3575/5293/7042/10365/9140/5603/1030/2911/3265/9456/3845/6794/57818/207/7043/1432/5571/1956/10018/11337/6502/5728/2309/5594/1901/4193/1027/7874/6789/1032/2033
hsa05416	Viral myocarditis	56	0.622056	1.612695	0.000393	0.001324	0.000571	8459	tags = 71%, list = 25%, signal = 54%	3135/857/3134/5880/3106/637/3105/3107/595/60/958/71/284217/3689/1981/5879/3133/1605/942/836/6444/54205/941/5551/3117/6443/3111/6442/3123/3122/841/3127/3112/842/3113/3115/3383/1982/25/3109
hsa04662		81	0.590121	1.570227	0.000395	0.001324	0.000571	9803		

(Continued)

Table S2: *Continued*

ID	Description	setSize	enrichmentScore	NES	*p* value	*p*.adjust	*q* value	rank	leading_edge	core_enrichment
	B cell receptor signaling pathway								tags = 63%, list = 29%, signal = 45%	84433/27071/5880/8915/353514/7410/11006/118788/4794/10288/5879/4067/79168/5595/4793/8519/5604/971/4790/23547/208/7409/5605/11024/10451/5291/2885/29760/5290/5777/4893/10990/5970/4772/5293/8517/5530/10859/3265/3845/11025/2213/207/4773/3635/975/25780/6850/2932/5594/5534
hsa04137	Mitophagy - animal	72	0.588318	1.54864	0.000479	0.00159	0.000685	11966	tags = 79%, list = 35%, signal = 52%	6237/598/285973/338382/10133/3091/7316/4286/283106/7879/6714/65018/23786/79065/9474/23710/51024/6667/4893/192111/5970/8878/1869/7311/8887/8678/6233/4077/1457/7030/3265/8408/3845/9927/55669/29110/81631/9101/1459/440738/1460/11337/2309/9958/54543/7157/22808/7314/468/22800/89941/3725/10241/11345/79735/64786/5601
hsa01250	Biosynthesis of nucleotide sugars	37	0.666832	1.632027	0.000494	0.001623	0.0007	9702	tags = 81%, list = 28%, signal = 58%	55276/80201/3099/9945/64841/2821/55907/5236/3101/3098/7264/7358/2582/54187/7360/2584/29926/2762/5373/23483/55577/10020/5238/29925/2585/6675/91373/2673/4351/2645
hsa04622	RIG-I-like receptor signaling pathway	71	0.58713	1.546272	0.000555	0.001807	0.000779	9527	tags = 56%, list = 28%, signal = 41%	338376/9636/3627/3576/64135/5600/8772/79132/23586/79671/6300/9641/4793/9474/4790/7187/10010/3592/3665/841/5970/7124/8717/8517/9140/5603/340061/3439/7706/3661/26007/29110/1432/8737/9755/7186/6885/103/5300/843

(Continued)

Table S2: *Continued*

ID	Description	setSize	enrichmentScore	NES	*p* value	*p*.adjust	*q* value	rank	leading_edge	core_enrichment
hsa00601	Glycosphingolipid biosynthesis - lacto and neolacto series	27	0.721057	1.690838	0.000598	0.001927	0.000831	4435	tags = 63%, list = 13%, signal = 55%	10331/10317/10690/53947/2525/8706/2528/84002/6484/2524/8702/2683/2526/2529/79369/8704/10678
hsa05212	Pancreatic cancer	76	0.580091	1.534411	0.000649	0.002071	0.000893	7814	tags = 59%, list = 23%, signal = 46%	7039/1026/598/5888/5899/1647/1021/5880/6772/4088/578/595/4616/7040/5879/5337/7046/10928/5595/5604/2064/3716/1870/4790/208/5898/1019/5291/7422/5290/581/7048/5970/1869/5293/7042/8517/675/842/5925/3845/1643/207/7043/6199
hsa04974	Protein digestion and absorption	103	0.548619	1.48095	0.000695	0.002197	0.000947	7216	tags = 50%, list = 21%, signal = 39%	1308/5646/5645/1294/3783/5644/1301/6564/1303/1291/1292/169044/1277/1281/1289/1300/1290/1306/1278/50509/1293/9056/483/10008/1284/486/1282/206358/6550/80781/136227/7512/1296/54407/478/1288/1295/340024/6505/1307/256076/4311/1299/1287/1305/6519/91522/6520/153201/1359/440387
hsa04978	Mineral absorption	60	0.602278	1.568123	0.000728	0.002281	0.000984	8305	tags = 52%, list = 24%, signal = 39%	26872/4489/4502/4499/261729/4493/4501/4495/4496/9843/55503/4494/3162/7421/483/486/6550/478/475/6569/493/340024/7779/6523/2495/55630/65010/490/2512/3163/4490
hsa05210	Colorectal cancer	86	0.56439	1.505726	0.000843	0.002617	0.001128	7814	tags = 53%, list = 23%, signal = 41%	2069/374/7039/5366/1026/4609/5899/332/1647/5880/4088/578/595/4616/7040/5879/7046/27113/6932/10297/1499/5595/836/5604/54205/208/5898/387/5605/5291/2885/5290/581/4893/7048/8312/5293/

(Continued)

Table S2: *Continued*

ID	Description	setSize	enrichmentScore	NES	*p* value	*p*.adjust	*q* value	rank	leading_edge	core_enrichment
										7042/842/3265/3845/1643/4437/207/7043/6199
hsa00052	Galactose metabolism	31	0.674599	1.622361	0.0009	0.002768	0.001193	5302	tags = 58%, list = 15%, signal = 49%	57016/5214/55276/80201/3099/2683/5236/93432/231/3101/3098/8972/2582/8704/7360/2584/5211/2717
hsa01521	EGFR tyrosine kinase inhibitor resistance	79	0.565997	1.505286	0.001011	0.00308	0.001328	9649	tags = 63%, list = 28%, signal = 46%	4233/7039/598/5582/5155/558/5154/253314/1978/3569/25759/3084/5578/6714/5159/2247/5595/5604/2064/6464/3716/208/56034/5605/5291/3717/3480/2885/7422/5290/581/2621/4893/5293/399694/1977/3265/3845/5156/207/6199/2065/1956/2261/9542/10018/5728/2932/2309/5594
hsa05320	Autoimmune thyroid disease	49	0.626536	1.596234	0.001047	0.003159	0.001362	7325	tags = 51%, list = 21%, signal = 40%	3135/3002/355/3134/3106/3105/3107/958/356/3133/7038/942/941/5551/3117/3111/3123/3122/3127/3586/3112/3113/3439/3115/1493
hsa00051	Fructose and mannose metabolism	33	0.666483	1.611135	0.001086	0.003247	0.0014	8060	tags = 67%, list = 23%, signal = 51%	57016/5214/80201/229/3099/226/7167/6652/231/3101/3098/57103/7264/5209/5211/29926/5210/2762/5373/26007/230/29925
hsa04142	Lysosome	132	0.525707	1.434741	0.001101	0.003263	0.001407	11082	tags = 64%, list = 32%, signal = 44%	1510/1515/1212/130340/1514/3373/23553/968/1508/1513/8372/1174/1521/1509/2760/527/2519/1176/8692/7805/9374/4074/5660/5476/10053/9516/10312/1520/162/2588/6556/1075/51172/2717/8943/26503/4668/3423/967/1519/9741/3916/9179/245972/57192/54/23659/2548/8546/4669/285362/23431/138050/1522/8907/8763/10239/4125/

(Continued)

Table S2: *Continued*

ID	Description	setSize	enrichmentScore	NES	*p* value	*p*.adjust	*q* value	rank	leading_edge	core_enrichment
										20/411/23457/53/4864/3988/3073/1211/1777/26985/3425/1203/3482/950/2581/533/51606/3920/8218/4126/1213/3074/9114/410/2720/2629/1200
hsa05017	Spinocerebellar ataxia	142	0.513656	1.405994	0.001117	0.003282	0.001415	12483	tags = 68%, list = 36%, signal = 44%	146227/116444/5582/6511/7416/6712/7417/5708/5714/2906/5704/4217/6261/10105/5686/5578/5331/56652/5691/10213/11047/4976/2892/54205/29982/60673/208/5689/5701/5291/5695/773/5707/6667/90550/5290/5685/5713/5715/23152/5690/5293/5705/5694/5332/9776/5717/5709/8678/5700/488/55062/2911/5702/293/8408/5688/5719/116443/3710/5718/207/30849/5693/26100/5706/5683/292/7186/11273/2959/5682/4800/10528/9861/5710/7979/6908/5649/2903/5173/5692/143471/9519/7419/5296/342371/5684/11317/5601/25814/23130/10939/2776/9698/2475/3516
hsa05215	Prostate cancer	97	0.549589	1.47851	0.001135	0.003306	0.001426	7066	tags = 48%, list = 21%, signal = 39%	5328/7850/7039/4314/6716/898/5327/4824/1026/4318/6692/5155/5154/595/84699/6932/5159/1499/5595/5604/2064/1870/4790/3320/208/56034/2308/5605/2950/1017/5291/3480/2885/9586/5290/4893/5970/1869/5293/8517/842/10488/6935/3326/5925/3265/3845
hsa04015	Rap1 signaling pathway	210	0.484072	1.350494	0.001244	0.003591	0.001548	7445	tags = 46%, list = 22%, signal = 36%	1943/4233/1969/9965/2250/57121/3690/1946/7424/6237/5582/5899/9564/7408/5155/3688/5880/2357/3397/5600/136/7410/

(Continued)

Table S2: *Continued*

ID	Description	setSize	enrichmentScore	NES	*p* value	*p*.adjust	*q* value	rank	leading_edge	core_enrichment
										5154/60/71/7074/3689/2771/3684/2533/999/5879/6300/808/1436/5578/9170/5331/7057/109/5216/6714/51806/9002/5159/2247/1499/5595/91860/64411/25865/2773/54518/5604/5908/5028/5606/1500/1944/208/56034/5898/387/7409/5605/2149/2246/1813/10451/5228/51378/5291/3480/56288/3937/7422/5290/1399/4893/6494/2324/5293/1435/2846/5332/5217/5603/7094/26037/805/2252/3265/3845/10636/8631/2321/5156
hsa01240	Biosynthesis of cofactors	151	0.513162	1.410293	0.001266	0.003623	0.001562	10463	tags = 58%, list = 31%, signal = 40%	250/54578/7364/8942/205/23057/251/54575/79799/54658/8836/129607/4143/3620/54576/6999/2729/93100/54577/195814/4831/1728/654364/248/27010/10797/122622/25902/219/8566/6697/112724/7358/2730/4830/53630/27430/2937/7360/4833/158067/7372/2356/29926/27235/4522/55163/26289/51109/80347/1738/55312/5373/159/790/51727/3145/11019/23498/3242/29968/125061/1503/29925/64802/80308/51805/1719/7390/65220/54600/10327/10201/60490/1355/4351/200895/102157402/158/7389/29922/80025/6472/79646/79717/50808/4832
hsa04140	Autophagy - animal	141	0.518192	1.417902	0.001321	0.00375	0.001617	11248	tags = 63%, list = 33%, signal = 43%	54541/1514/6237/598/118471/285973/338382/3667/1508/3091/1613/1509/84335/10533/7879/4218/55054/5595/1965/5604/

(Continued)

Table S2: *Continued*

ID	Description	setSize	enrichmentScore	NES	*p* value	*p*.adjust	*q* value	rank	leading_edge	core_enrichment
										79065/29982/60673/9474/58476/208/10010/5605/5515/5563/23710/5291/3480/64419/5290/440275/4893/8878/5293/9342/64422/3916/9776/8678/8837/9140/53349/3146/55062/5861/51100/3265/8408/3845/6794/10645/5516/8673/3476/207/29110/6199/30849/83734/26100/64223/81631/57521/81671/5566/64121/440738/6885/9711/11337/5728/55255/5594/3920/5567/10670/22808/6198/140775/115201/22800/5562/5580/55014
hsa04066	HIF-1 signaling pathway	109	0.540323	1.463956	0.001355	0.003812	0.001644	7843	tags = 54%, list = 23%, signal = 42%	6513/5054/112399/5214/1906/80201/229/1026/2597/3099/5582/7076/92483/3162/4055/3939/226/3091/3458/5230/3459/253314/1978/3569/2023/4843/3101/3098/6923/5578/2026/7037/5595/5604/2064/4790/208/5209/5605/1536/2872/51378/5291/5211/3480/7099/7422/5290/5970/5293/1977/3945/6921/818/2321/54583/207/6199/230
hsa00140	Steroid hormone biosynthesis	59	0.595	1.548284	0.00154	0.004297	0.001853	3098	tags = 32%, list = 9%, signal = 29%	54578/6820/7364/6716/54575/1577/79799/54658/79644/1583/3294/54576/1312/54577/3290/6715/1109/1646/412
hsa05143	African trypanosomiasis	36	0.64839	1.573151	0.001648	0.00456	0.001966	6268	tags = 61%, list = 18%, signal = 50%	2150/8542/3606/5582/355/3620/4615/3458/7412/3553/3569/3827/356/5578/5331/7064/3910/3592/3040/3586/7124/5332
hsa04071	Sphingolipid signaling pathway	118	0.528065	1.433183	0.0018	0.004938	0.002129	9766	tags = 60%, list = 28%, signal = 43%	5522/8877/134/130367/5582/5880/7132/637/5600/53637/2207/1509/2771/4217/

(Continued)

Table S2: *Continued*

ID	Description	setSize	enrichmentScore	NES	*p* value	*p*.adjust	*q* value	rank	leading_edge	core_enrichment
										2768/3827/5879/6300/4363/5337/166929/624/5578/5331/10558/5595/204219/2773/5604/8698/4790/208/387/5605/5515/5518/8879/5291/5520/5290/5526/581/4893/5970/7124/8717/5293/1903/253782/5332/10672/5521/5603/56624/3265/3845/5516/6610/207/1432/340485/5338/7186/10715/8560/4846/5728/8439/5594/1901/56848
hsa04390	Hippo signaling pathway	156	0.502323	1.380189	0.001913	0.005206	0.002245	9867	tags = 62%, list = 29%, signal = 44%	7476/374/5522/655/80326/330/5054/7477/122786/6591/4609/332/3397/4088/7004/89780/7161/85409/268/166824/595/7534/60/71/1855/7532/7040/3689/2736/126374/999/9231/658/1495/7533/2535/7005/26524/7046/652/1454/27113/3996/6932/10297/1499/1490/11211/329/8200/7483/7475/5515/5518/7529/2246/894/55233/56288/5499/10413/5520/3993/7048/8312/7042/4086/1857/7003/5521/8324/7531/5516/10971/7472/7043/60485/896/23513/174/8945/8321/4771/84552/6934/5500/659/5501/84962/25937/8463/2932/392255/23291/1742/1453
hsa04936	Alcoholic liver disease	142	0.506714	1.386992	0.001985	0.005359	0.002311	9630	tags = 51%, list = 28%, signal = 37%	3929/3576/148022/355/714/7132/712/929/713/5600/4615/8772/595/3553/728/50507/2919/719/718/4217/3569/6300/356/219/6319/3654/9641/79602/6932/1499/836/5606/4790/7187/208/2308/5563/3592/

(Continued)

Table S2: *Continued*

ID	Description	setSize	enrichmentScore	NES	*p* value	*p*.adjust	*q* value	rank	leading_edge	core_enrichment
										7099/841/5970/7124/8717/2921/2168/8517/23118/23765/5603/127/3439/9020/23643/131/10645/3661/6720/84818/207/29110/1432/5571/8737/5609/128/130/6934/6885/51094/2920/51/2932/2309
hsa05410	Hypertrophic cardiomyopathy	90	0.548485	1.470057	0.002169	0.005806	0.002504	5331	tags = 42%, list = 16%, signal = 36%	3691/3673/27092/59285/3675/3655/3696/3690/1906/3694/3678/7171/3688/3693/22801/3685/7137/7169/4000/59283/7139/60/71/284217/7040/786/3569/1605/7170/3695/6444/3672/1636/7168/5563/6443/93589/6442
hsa05211	Renal cell carcinoma	68	0.573117	1.506037	0.002396	0.006364	0.002744	10607	tags = 68%, list = 31%, signal = 47%	6513/4233/7039/112399/1026/5155/3091/10298/7040/5062/5879/6923/2113/5058/5595/5604/5908/208/5605/5291/2885/7422/5290/1399/56924/4893/5293/7042/7030/3265/6921/3845/54583/5781/207/7043/9978/1398/8453/57144/2889/5594/5906/998/2033/9915
hsa00240	Pyrimidine metabolism	58	0.591032	1.532596	0.002457	0.006475	0.002792	8042	tags = 60%, list = 23%, signal = 46%	978/4907/7083/6241/129607/1890/7371/7378/124583/284958/318/4831/5167/654364/7298/377841/4830/1841/1806/4833/1635/51251/1854/7372/7084/22978/30833/79077/8623/790/51727/6240/955/51020/1503
hsa04658	Th1 and Th2 cell differentiation	89	0.545668	1.463135	0.002961	0.007739	0.003337	8728	tags = 61%, list = 25%, signal = 45%	4854/182/6772/3566/5600/3458/2625/3560/3559/3459/3561/4794/6300/6776/30009/3595/915/3932/3594/864/5595/4793/3716/4790/84441/920/919/916/3117/3718/3717/3592/3111/3123/917/3122/3127/

(Continued)

Table S2: *Continued*

ID	Description	setSize	enrichmentScore	NES	*p* value	*p*.adjust	*q* value	rank	leading_edge	core_enrichment
										6778/5970/3112/4772/8517/5530/5603/4094/3113/3115/3714/1432/4773/3109/6775/7535/4853
hsa04012	ErbB signaling pathway	84	0.545614	1.456879	0.003052	0.007915	0.003413	10788	tags = 68%, list = 31%, signal = 47%	2069/374/7039/1026/5582/4609/1839/10298/4690/1978/5062/25759/6776/3084/5578/6714/5058/5595/5604/2064/6464/208/5605/5291/2885/5290/8440/1399/56924/10718/4893/5293/2002/399694/3265/3845/818/685/207/6199/2065/5609/25/1398/1956/9542/868/57144/145957/2932/5594/1027/27/6416/6198/867/815
hsa05340	Primary immunodeficiency	37	0.619832	1.516996	0.003765	0.009689	0.004178	6306	tags = 54%, list = 18%, signal = 44%	6890/6891/100/5896/57379/958/3561/925/915/3932/29851/920/916/7374/3718/926/29760/3575/8517/84876
hsa04215	Apoptosis - multiple species	32	0.637069	1.538429	0.004371	0.011078	0.004777	7778	tags = 66%, list = 23%, signal = 51%	330/5366/598/332/7132/637/79444/578/8772/27113/836/329/54205/841/581/666/840/842/8678/27429/56616
hsa04216	Ferroptosis	41	0.603411	1.497527	0.004343	0.011078	0.004777	10202	tags = 71%, list = 30%, signal = 50%	23657/23516/55240/2729/3162/2182/5621/7417/1356/10533/2730/7037/5093/2937/9474/6303/2181/1536/2495/2879/10162/6520/8031/2512/81631/440738/30061/7157/5094
hsa00510	N-Glycan biosynthesis	50	0.587363	1.502208	0.00445	0.011194	0.004827	12128	tags = 70%, list = 35%, signal = 45%	146664/79644/4124/2683/11282/10195/4248/8704/4247/2530/4245/84920/6184/11253/1603/22845/4249/57171/1650/85365/7841/3703/56052/8813/440138/1798/29880/199857/23193/6185/84620/79087/201595/144245/79053

(Continued)

Table S2: *Continued*

ID	Description	setSize	enrichmentScore	NES	*p* value	*p*.adjust	*q* value	rank	leading_edge	core_enrichment
hsa04370	VEGF signaling pathway	59	0.571827	1.487983	0.004522	0.011288	0.004867	10071	tags = 68%, list = 29%, signal = 48%	8877/5829/5582/283748/5880/5600/9047/25759/5879/6300/5578/6714/8605/5595/7867/5604/3315/208/5605/5291/123745/7422/5290/4893/5293/5530/842/5603/9261/3265/3845/5743/207/1432/4773/4846/5594/56848/5534/998
hsa05221	Acute myeloid leukemia	67	0.556778	1.460518	0.00513	0.012709	0.00548	7814	tags = 55%, list = 23%, signal = 43%	8900/5371/890/4609/3728/597/5467/929/5292/595/2209/4353/1978/3684/6688/6776/1436/1437/6932/5595/5604/4790/208/5605/5291/1848/2885/5290/4893/5970/5293/8517/3265/3845/1053/207/6199
hsa00533	Glycosaminoglycan biosynthesis - keratan sulfate	14	0.741298	1.542389	0.005388	0.013251	0.005714	4435	tags = 57%, list = 13%, signal = 50%	10164/4166/8702/2683/6482/8704/2530/10678
hsa04672	Intestinal immune network for IgA production	45	0.579563	1.461706	0.005723	0.01397	0.006024	8459	tags = 62%, list = 25%, signal = 47%	3601/56477/4055/57379/958/7040/3600/3569/10673/942/3695/29851/8741/941/3117/3111/3123/3122/3127/3586/3112/3113/9020/3115/7852/102723996/23308/3109
hsa00520	Amino sugar and nucleotide sugar metabolism	49	0.58414	1.488221	0.006282	0.015224	0.006564	9757	tags = 78%, list = 28%, signal = 56%	55276/80201/3099/9945/64841/2821/55907/5236/3101/3098/7264/7358/2582/54187/51005/7360/2584/29926/1727/51167/1118/2762/80896/51700/5373/55577/10020/10007/5238/29925/2585/3073/6675/91373/2673/4351/2645/132789
hsa03030	DNA replication	36	0.613074	1.487467	0.006823	0.016415	0.007078	8204	tags = 69%, list = 24%, signal = 53%	4174/57804/4173/4171/4175/5984/4176/2237/10535/6742/5425/54107/5424/4172/5111/246243/6119/5982/5983/3978/5557/5558/23649/6117/56655
hsa05219	Bladder cancer	41	0.594605	1.475672	0.00694	0.016577	0.007148	10158		

(Continued)

Table S2: *Continued*

ID	Description	setSize	enrichmentScore	NES	*p* value	*p*.adjust	*q* value	rank	leading_edge	core_enrichment
									tags = 78%, list = 30%, signal = 55%	4312/3576/1026/4609/4318/1890/1839/4313/595/1613/999/7057/6714/5595/5604/2064/1870/5605/1019/7422/4893/1869/5925/3265/3845/1956/2261/1029/5594/1871/4193/7157
hsa00500	Starch and sucrose metabolism	36	0.612622	1.48637	0.007071	0.01665	0.00718	4911	tags = 47%, list = 14%, signal = 41%	55276/80201/3099/5836/5834/2632/2821/5167/5236/93432/3101/3098/8972/283209/2992/7360/2997
hsa04926	Relaxin signaling pathway	129	0.495991	1.350102	0.00707	0.01665	0.00718	7066	tags = 44%, list = 21%, signal = 35%	4312/4322/2769/1906/3640/7424/1277/4318/1281/4313/1278/5600/55970/1284/7040/59/2786/2771/84699/25759/6300/1282/4843/2793/5578/5331/7046/109/6714/2790/59345/5595/1288/2773/5604/6464/2783/4790/208/5605/5291/2885/9586/7422/5290/4893/7048/5970/5293/1287/10488/5332/399694/5603/10681/3265/3845
hsa05144	Malaria	49	0.57832	1.473394	0.007467	0.017459	0.007528	9074	tags = 61%, list = 26%, signal = 45%	4233/3606/3576/7058/4615/3458/7412/958/3553/7040/3689/3569/6347/7057/3592/7099/3040/3586/7124/7042/6382/4035/7043/3383/22914/3820/2995/3043/1311/975
hsa00983	Drug metabolism - other enzymes	77	0.534412	1.415625	0.007712	0.017905	0.007721	5625	tags = 43%, list = 16%, signal = 36%	978/7498/54578/7364/54575/7083/79799/54658/6241/119391/1890/7371/54576/9446/7378/54577/4353/4831/654364/4257/4830/4258/1806/4833/2950/3614/1854/8833/3251/7372/7084/10/9
hsa00830	Retinol metabolism	65	0.548474	1.434843	0.008476	0.019541	0.008426	7083	tags = 46%, list = 21%, signal = 37%	54578/10170/1559/7364/1562/29785/54575/1577/339761/79799/54658/220/54905/

(Continued)

Table S2: *Continued*

ID	Description	setSize	enrichmentScore	NES	*p* value	*p*.adjust	*q* value	rank	leading_edge	core_enrichment
										54576/54577/5959/195814/56603/1555/54884/112724/53630/158835/8228/6121/8694/51109/1576/127/131
hsa01524	Platinum drug resistance	72	0.539058	1.418971	0.008587	0.01966	0.008478	7987	tags = 51%, list = 23%, signal = 40%	1244/330/5366/1026/119391/598/332/355/9446/637/578/8772/7153/672/4217/356/27113/4257/4258/5595/836/329/54205/2064/208/2950/5291/841/5290/581/5293/842/2067/4437/207/4259/2944
hsa05225	Hepatocellular carcinoma	167	0.477638	1.319174	0.009829	0.02235	0.009637	9669	tags = 53%, list = 28%, signal = 38%	7476/4233/7039/80326/7477/1026/119391/598/5582/4609/1647/1021/7296/4088/9446/3162/89780/578/595/60/4616/71/1855/7040/1728/25759/5578/1452/2535/7046/6932/10297/4257/4258/1499/5595/11211/5604/6464/3481/1870/208/7483/7475/86/5605/2950/1019/5291/3480/2885/5290/581/9817/4893/7048/1869/8312/5293/7042/2002/8193/1857/399694/5925/4780/6598/3265/8324/3845/1643/7472/207/7043/6199/4259/2944/8110/1956/8321/114112/4041/6934/6601/5728/1029/2932/5594/1871
hsa03420	Nucleotide excision repair	46	0.580249	1.468	0.011281	0.025371	0.01094	12052	tags = 80%, list = 35%, signal = 52%	57804/5984/5886/5425/5887/54107/5424/5111/1022/6119/5982/5983/3978/1069/2067/1643/8451/2967/8450/6117/2068/56655/9978/2071/2965/2073/5985/1642/1161/10714/902/5427/404672/7508/6118/5981/5426
hsa00230	Purine metabolism	128	0.491915	1.337217	0.011386	0.025371	0.01094	9856	tags = 52%, list = 29%, signal = 37%	9615/7498/205/4907/954/55276/6241/100/124583/284958/318/5143/4860/144811/

(Continued)

Table S2: *Continued*

ID	Description	setSize	enrichmentScore	NES	*p* value	*p*.adjust	*q* value	rank	leading_edge	core_enrichment
										4831/5141/5167/5236/654364/122622/377841/109/272/353/4830/471/2987/10846/50940/10606/4833/5138/3614/158067/8833/3251/22978/2618/26289/30833/1716/159/374659/6240/5471/955/51020/271/132/9061/3704/196883/3615/5148/5147/10201/5634/957/102157402/158/953/29922/8654/131870/5152/9060
hsa04114	Oocyte meiosis	130	0.484677	1.320889	0.011341	0.025371	0.01094	10829	tags = 58%, list = 32%, signal = 40%	898/5347/9088/9133/991/4085/891/995/983/151648/5600/699/7534/7532/9700/6300/808/7533/9748/6790/109/9232/51806/5595/91860/5604/5515/996/5518/8379/7529/1017/10459/3480/5499/5526/26271/5530/5603/27330/805/441272/64682/818/6500/7531/5516/3710/10971/51529/8881/8454/1432/132864/801/25847/9978/8945/9126/196883/5566/10393/5500/64506/5501/246184/5594/23291/5534/5567/5528/5529/6195/815/51433/286151
hsa03013	Nucleocytoplasmic transport	108	0.508096	1.375211	0.012531	0.027734	0.011959	11813	tags = 69%, list = 34%, signal = 45%	1917/402569/56000/5905/3838/51068/5901/10189/23636/3840/6612/79023/55706/84321/23534/9775/53371/59343/22916/3839/84305/55161/3836/10762/6396/10073/3837/80145/3843/10526/7329/3842/29107/1915/9688/51808/5976/55110/94026/7341/4686/10284/23165/6613/23214/23633/79228/30000/57122/10527/51194/7514/

(Continued)

Table S2: *Continued*

ID	Description	setSize	enrichmentScore	NES	*p* value	*p*.adjust	*q* value	rank	leading_edge	core_enrichment
										1434/9883/4116/10921/9631/9670/55308/8086/8480/79902/100101267/4928/9972/23511/9939/23039/8563/23225/57510/8021/348995/64328
hsa04540	Gap junction	88	0.514951	1.380598	0.013026	0.02864	0.012349	7445	tags = 48%, list = 22%, signal = 37%	10381/7277/84617/5582/79861/5155/5593/84790/983/2697/10376/5154/2771/3358/5578/5331/109/203068/2767/6714/5159/5595/10383/2773/5604/7082/56034/5605/5592/1813/2885/4893/2915/7846/5332/2911/3265/5607/5598/3845/3710/5156
hsa04722	Neurotrophin signaling pathway	119	0.486818	1.322763	0.01321	0.028852	0.012441	12183	tags = 70%, list = 36%, signal = 45%	3656/4909/8767/4145/3667/5600/7161/4794/396/4217/25759/5879/6300/356/808/3654/11213/51806/5595/91860/4793/5604/5908/6464/4790/208/387/5605/5291/5663/2885/4908/5290/627/581/1399/4893/5970/5293/10603/399694/10019/5603/11108/27330/805/9261/3265/5607/5598/3845/818/7531/5781/207/1432/5609/25/801/27018/1398/2889/2932/2309/5594/5664/5906/998/7157/4914/6195/468/815/51135/4804/5580/3725/5296/4915/814/5601/2549/10782
hsa04919	Thyroid hormone signaling pathway	121	0.499787	1.357703	0.013498	0.029058	0.01253	10158	tags = 52%, list = 30%, signal = 37%	6513/113026/5214/3690/1734/5582/1735/4609/4854/6567/6772/3685/3091/483/595/60/71/7068/486/6548/5578/5331/652/6714/5333/478/1499/5595/5604/208/2308/5350/5605/5291/5211/5290/4893/5293/842/3066/

(Continued)

Table S2: *Continued*

ID	Description	setSize	enrichmentScore	NES	*p* value	*p*.adjust	*q* value	rank	leading_edge	core_enrichment
										5332/6256/488/3065/3265/8202/3845/207/5469/8841/9862/10025/4853/2626/9882/5566/9282/25942/2932/5594/4193/5567/7157
hsa05414	Dilated cardiomyopathy	95	0.500753	1.345549	0.013483	0.029058	0.01253	5331	tags = 38%, list = 16%, signal = 32%	3691/3673/27092/59285/3675/3655/3696/3690/3694/3678/7171/3688/3693/22801/3685/7137/7169/4000/59283/7139/60/71/284217/7040/786/1605/109/7170/3695/6444/3672/7168/5350/6443/93589/6442
hsa01200	Carbon metabolism	115	0.494124	1.338891	0.013566	0.029058	0.01253	9946	tags = 59%, list = 29%, signal = 42%	5214/80201/229/2597/3099/5315/5223/226/7167/5230/113675/2821/2023/2746/25796/3101/3098/5226/441531/2026/10993/39/2539/55753/5211/6888/4190/6120/9563/3419/4967/50/3417/6389/2806/1738/1373/2747/4191/8801/283871/2805/83440/1737/26007/1892/6390/230/729020/2098/29968/8803/132158/128/4199/7086/1431/5634/3420/3421/1743/51/5232/5091/6392/2645/5723/6472
hsa05226	Gastric cancer	149	0.474083	1.303501	0.015501	0.03299	0.014225	7814	tags = 47%, list = 23%, signal = 36%	7476/4233/4583/83998/80326/9965/2250/1015/7477/898/1026/4609/1647/3728/4088/89780/578/595/4616/1855/7040/999/25759/1495/1452/2535/7046/1045/6932/10297/2247/1499/5595/11211/5915/5604/2064/6464/1870/208/7483/7475/5605/2246/1017/5291/2885/5290/581/4893/7048/1869/8312/5293/7042/1857/399694/6256/5925/1030/

(Continued)

Table S2: *Continued*

ID	Description	setSize	enrichmentScore	NES	*p* value	*p*.adjust	*q* value	rank	leading_edge	core_enrichment
										2252/3265/8324/3845/1643/7472/8074/207/7043/6199
hsa00532	Glycosaminoglycan biosynthesis - chondroitin sulfate / dermatan sulfate	21	0.646335	1.453972	0.018314	0.038482	0.016593	10434	tags = 81%, list = 30%, signal = 56%	9469/29940/166012/55790/79586/50515/51363/113189/54480/22856/55454/337876/126792/10090/64132/64131/11285
hsa04664	Fc epsilon RI signaling pathway	67	0.524757	1.376521	0.018229	0.038482	0.016593	8119	tags = 51%, list = 24%, signal = 39%	283748/5880/240/5600/7410/2207/241/5879/6300/4067/5578/1437/8605/5595/5604/5606/208/7409/5605/10451/5291/123745/2885/3937/5290/4893/7124/5293/5603/3265/3845/207/1432/5609
hsa03040	Spliceosome	152	0.465374	1.279005	0.018869	0.039399	0.016989	13287	tags = 71%, list = 39%, signal = 44%	3310/3312/10189/5093/84844/51729/84321/101954273/9343/6628/9775/3303/6633/22916/83443/11338/6637/6626/8175/55660/22827/51639/153527/25804/9128/4809/10285/9416/51645/6632/9092/5356/11157/3304/6627/8896/26121/3306/10286/27258/27339/9785/22938/23658/8683/57461/1659/10523/11017/56259/3190/9716/6631/55110/9410/4686/10291/4670/10262/151903/6636/1665/56949/55696/10465/10569/3183/10084/10946/23450/3305/10992/4116/6634/51362/25949/57819/51340/84991/10713/23350/51690/6629/6426/8449/6635/23020/988/9939/6434/6428/6625/3178/51503/199746/11325/51691/10915/10929/24148/9879/6429/6427/3192/220988/6432/6430/10594

(Continued)

Table S2: *Continued*

ID	Description	setSize	enrichmentScore	NES	*p* value	*p*.adjust	*q* value	rank	leading_edge	core_enrichment
hsa05230	Central carbon metabolism in cancer	69	0.516372	1.355048	0.019807	0.0411	0.017722	7066	tags = 49%, list = 21%, signal = 39%	6513/4233/9123/5214/80201/3099/5315/4609/92483/3939/5223/3091/3101/3098/57103/441531/8140/5159/51548/5595/5604/2064/2539/208/5605/5291/5211/5290/4893/3417/5293/3945/3265/3845
hsa00010	Glycolysis / Gluconeogenesis	67	0.5198	1.363519	0.021345	0.044015	0.018979	7401	tags = 51%, list = 22%, signal = 40%	222/5214/55276/80201/229/2597/218/3099/5315/92483/3939/5223/226/7167/5230/2821/2023/5236/3101/3098/219/441531/2026/221/669/5211/9562/1738/3945/127/131/83440/1737/57818
hsa00030	Pentose phosphate pathway	30	0.601099	1.442439	0.022988	0.047112	0.020315	9025	tags = 67%, list = 26%, signal = 49%	5214/55276/229/226/51071/2821/5236/25796/64080/5226/2539/5211/6888/6120/9563/230/729020/132158/7086/5634
hsa04152	AMPK signaling pathway	121	0.486048	1.32038	0.023492	0.047848	0.020632	11493	tags = 55%, list = 34%, signal = 37%	3172/5522/5468/8900/5214/890/3667/23216/595/1978/84699/84335/51719/6319/10890/4218/79602/3952/208/2308/5209/5515/5518/5563/2997/5291/1938/5211/3480/9586/5520/5290/5526/5210/5293/10488/3156/5521/8408/6794/10645/5516/2998/57818/6720/207/6199/9230/5571/57521/5207/6885/51094/2309/90993/200186/5528/5529/1994/51552/6198/5862/81617/31/5562/92579/5296
hsa04150	mTOR signaling pathway	155	0.462209	1.270365	0.024605	0.04981	0.021478	11837	tags = 61%, list = 35%, signal = 40%	7476/54541/80326/7477/5582/7132/89780/3667/79899/1855/253314/1978/6446/2887/84335/51719/5578/2535/8140/5595/11211/6249/5604/9296/208/529/387/7483/7475/

(Continued)

Table S2: *Continued*

ID	Description	setSize	enrichmentScore	NES	*p* value	*p*.adjust	*q* value	rank	leading_edge	core_enrichment
										5605/5563/5291/3480/2885/5290/51382/4893/7124/5293/6396/1857/1977/27330/79109/6520/3265/8324/8408/3845/6794/55615/9894/526/7472/207/6199/64223/57521/8321/4041/1975/64121/10542/84219/6502/9550/5728/729438/2932/5594/8649/389541/51606/10670/55004/7480/6198/9681/9470/6195/9675/81029/81617/7474/528/28956/1147/5562/8323/523/5296/153129/7471/1856

Table S3: The 35 targeted miRNA of *GJB3* in the mirDIP database

Gene Symbol	Uniprot	MicroRNA	Integrated Score	Number of Sources	Score Class	Sources
GJB3	O75712	hsa-miR-4510	0.500054122	14	Very High	bitargeting_May_2021|DIANA|MBStar|MirAncesTar|miranda_May_2021|miRDB_v6|mirmap_May_2021|MiRNATIP|MirSNPInTarget|MirTar2|mirzag|PITA_May_2021|RNA22|rnahybrid_May_2021
GJB3	O75712	hsa-miR-2110	0.372904894	11	High	bitargeting_May_2021|DIANA|MirAncesTar|miranda_May_2021|mirCoX|miRDB_v6|MiRNATIP|MirTar2|mirzag|PITA_May_2021|RNA22
GJB3	O75712	hsa-miR-6127	0.350568369	10	High	bitargeting_May_2021|MBStar|MirAncesTar|miranda_May_2021|miRDB_v6|mirmap_May_2021|MiRNATIP|mirzag|PITA_May_2021|RNA22
GJB3	O75712	hsa-miR-6130	0.348206382	9	High	bitargeting_May_2021|MirAncesTar|miranda_May_2021|miRDB_v6|mirmap_May_2021|MiRNATIP|mirzag|PITA_May_2021|RNA22
GJB3	O75712	hsa-miR-6129	0.33400524	9	High	bitargeting_May_2021|MirAncesTar|miranda_May_2021|miRDB_v6|mirmap_May_2021|MiRNATIP|mirzag|PITA_May_2021|RNA22
GJB3	O75712	hsa-miR-6133	0.323949001	9	High	bitargeting_May_2021|MirAncesTar|miranda_May_2021|miRDB_v6|mirmap_May_2021|MiRNATIP|mirzag|PITA_May_2021|RNA22
GJB3	O75712	hsa-miR-3605-5p	0.308312803	8	High	bitargeting_May_2021|DIANA|MirAncesTar|miranda_May_2021|miRDB_v6|MiRNATIP|MirTar2|RNA22
GJB3	O75712	hsa-miR-885-3p	0.304405742	11	High	bitargeting_May_2021|DIANA|MirAncesTar|miranda_May_2021|mirbase|miRDB_v6|mirmap_May_2021|MiRNATIP|mirzag|PITA_May_2021|RNA22
GJB3	O75712	hsa-miR-4727-5p	0.289761296	8	High	bitargeting_May_2021|DIANA|MBStar|MirAncesTar|miranda_May_2021|miRDB_v6|MiRNATIP|mirzag
GJB3	O75712	hsa-miR-4722-3p	0.288631163	8	High	bitargeting_May_2021|MBStar|MirAncesTar|MiRNATIP|MirSNPInTarget|mirzag|PITA_May_2021|RNA22
GJB3	O75712	hsa-miR-4721	0.285687282	10	High	bitargeting_May_2021|DIANA|MirAncesTar|miranda_May_2021|miRDB_v6|mirmap_May_2021|MiRNATIP|mirzag|PITA_May_2021|RNA22
GJB3	O75712	hsa-miR-3663-3p	0.257018937	9	High	bitargeting_May_2021|DIANA|MirAncesTar|miranda_May_2021|mirmap_May_2021|MiRNATIP|mirzag|PITA_May_2021|RNA22
GJB3	O75712	hsa-miR-668-3p	0.252177223	7	High	BCmicrO|bitargeting_May_2021|MirAncesTar|miranda_May_2021|mirbase|mirzag|PITA_May_2021
GJB3	O75712	hsa-miR-941	0.246540685	8	High	

(Continued)

Table S3: *Continued*

Gene Symbol	Uniprot	MicroRNA	Integrated Score	Number of Sources	Score Class	Sources
						BCmicrO|bitargeting_May_2021|DIANA|MirAncesTar|miranda_May_2021|mirbase|mirmap_May_2021|MiRNATIP
GJB3	O75712	hsa-miR-6783-3p	0.227674678	7	High	bitargeting_May_2021|MirAncesTar|miranda_May_2021|miRDB_v6|MiRNATIP|mirzag|RNA22
GJB3	O75712	hsa-miR-3173-5p	0.225223676	6	High	bitargeting_May_2021|DIANA|MirAncesTar|miRDB_v6|MiRNATIP|MirSNPInTarget
GJB3	O75712	hsa-miR-6800-3p	0.223419609	7	Very High	bitargeting_May_2021|MirAncesTar|miranda_May_2021|MiRNATIP|mirzag|PITA_May_2021|RNA22
GJB3	O75712	hsa-miR-6796-5p	0.214841252	8	High	bitargeting_May_2021|MirAncesTar|miranda_May_2021|miRDB_v6|MiRNATIP|mirzag|PITA_May_2021|RNA22
GJB3	O75712	hsa-miR-4507	0.213428186	8	High	bitargeting_May_2021|DIANA|MBStar|MirAncesTar|MiRNATIP|mirzag|PITA_May_2021|RNA22
GJB3	O75712	hsa-miR-6511b-5p	0.205784794	7	High	MirAncesTar|miranda_May_2021|miRDB_v6|MiRNATIP|mirzag|PITA_May_2021|RNA22
GJB3	O75712	hsa-miR-6799-3p	0.201158917	6	High	bitargeting_May_2021|MirAncesTar|miranda_May_2021|miRDB_v6|MiRNATIP|PITA_May_2021
GJB3	O75712	hsa-miR-1911-5p	0.190825576	9	High	BCmicrO|bitargeting_May_2021|MBStar|MirAncesTar|miranda_May_2021|mirmap_May_2021|MiRNATIP|PITA_May_2021|RNA22
GJB3	O75712	hsa-miR-6793-5p	0.185148489	6	High	MirAncesTar|miranda_May_2021|miRDB_v6|MiRNATIP|mirzag|RNA22
GJB3	O75712	hsa-miR-134-3p	0.178967449	7	High	bitargeting_May_2021|MirAncesTar|miranda_May_2021|MiRNATIP|mirzag|PITA_May_2021|RNA22
GJB3	O75712	hsa-miR-6890-3p	0.177338017	7	High	bitargeting_May_2021|MirAncesTar|miranda_May_2021|mirmap_May_2021|MiRNATIP|mirzag|PITA_May_2021
GJB3	O75712	hsa-miR-6744-3p	0.173567771	6	High	bitargeting_May_2021|MirAncesTar|miranda_May_2021|MiRNATIP|PITA_May_2021|RNA22
GJB3	O75712	hsa-miR-6742-3p	0.172382613	6	High	bitargeting_May_2021|MirAncesTar|miranda_May_2021|MiRNATIP|mirzag|PITA_May_2021
GJB3	O75712	hsa-miR-6811-5p	0.169962222	6	High	bitargeting_May_2021|MirAncesTar|miranda_May_2021|miRDB_v6|MiRNATIP|RNA22
GJB3	O75712	hsa-miR-6821-3p	0.166691196	6	High	bitargeting_May_2021|MirAncesTar|miranda_May_2021|mirzag|PITA_May_2021|RNA22
GJB3	O75712	hsa-miR-6834-3p	0.162606963	5	High	bitargeting_May_2021|miranda_May_2021|miRDB_v6|MiRNATIP|PITA_May_2021
GJB3	O75712	hsa-miR-1296-3p	0.150431516	6	High	bitargeting_May_2021|MirAncesTar|miranda_May_2021|MiRNATIP|mirzag|RNA22
GJB3	O75712	hsa-miR-3940-3p	0.147507896	6	High	bitargeting_May_2021|DIANA|MirAncesTar|miranda_May_2021|mirmap_May_2021|MiRNATIP
GJB3	O75712	hsa-miR-6751-3p	0.145280116	5	High	MirAncesTar|miranda_May_2021|miRDB_v6|MiRNATIP|PITA_May_2021
GJB3	O75712	hsa-miR-6861-3p	0.144420757	5	High	bitargeting_May_2021|MirAncesTar|miranda_May_2021|mirzag|RNA22

Table S4: The correlation between *GJB3* and drugs

Symbol	Correlation	*p* value
VX.11e_2096	−0.448092	2.43×10^{-27}
Trametinib_1372	−0.428742	6.23×10^{-25}
Selumetinib_1736	−0.421334	1.08×10^{-20}
Luminespib_1559	−0.410783	1.38×10^{-18}
WIKI4_1940	−0.403027	4.34×10^{-15}
BMS.536924_1091	−0.372401	9.52×10^{-19}
ERK_6604_1714	−0.365364	4.68×10^{-18}
ERK_2440_1713	−0.352268	8.20×10^{-17}
IGF1R_3801_1738	−0.336939	1.98×10^{-15}
PD0325901_1060	−0.326528	1.56×10^{-14}
AZD6738_1917	−0.325595	1.87×10^{-14}
Dasatinib_1079	−0.322623	3.31×10^{-14}
Ulixertinib_1908	−0.31892	6.71×10^{-14}
MK.1775_1179	−0.280034	6.21×10^{-11}
Sapitinib_1549	−0.279713	6.55×10^{-11}
AZD2014_1441	−0.276505	1.10×10^{-10}
Foretinib_2040	−0.276182	1.16×10^{-10}
Taselisib_1561	−0.272869	1.96×10^{-10}
Pictilisib_1058	−0.271678	2.36×10^{-10}
Entospletinib_1630	−0.270706	2.75×10^{-10}
X5.Fluorouracil_1073	−0.265197	6.46×10^{-10}
AZD1332_1463	−0.261356	1.16×10^{-9}
AZD7762_1022	−0.260986	1.22×10^{-9}
Mirin_1048	−0.254187	3.35×10^{-9}
VE821_2111	−0.2497	6.42×10^{-9}
Docetaxel_1819	−0.246068	1.08×10^{-8}
Staurosporine_1034	−0.242507	1.77×10^{-8}
AZD8186_1918	−0.238046	3.27×10^{-8}
Buparlisib_1873	−0.225543	1.71×10^{-7}
Alpelisib_1560	−0.224878	1.87×10^{-7}
Alisertib_1051	−0.221425	2.90×10^{-7}
Bortezomib_1191	−0.219188	3.84×10^{-7}
Docetaxel_1007	−0.219047	3.90×10^{-7}
PLX.4720_1036	−0.211228	1.02×10^{-6}
Cediranib_1922	−0.203601	2.50×10^{-6}
KU.55933_1030	−0.203374	2.57×10^{-6}
AZD3759_1915	−0.200979	3.39×10^{-6}
YK.4.279_1239	−0.198883	4.30×10^{-6}
Lapatinib_1558	−0.198744	4.37×10^{-6}
GNE.317_1926	−0.195093	6.57×10^{-6}

Table S4: *Continued*

Symbol	Correlation	*p* value
Vinorelbine_2048	−0.19293	8.34×10^{-6}
BMS.345541_1249	−0.186269	1.71×10^{-5}
GSK2606414_1618	−0.184775	2.00×10^{-5}
Afatinib_1032	−0.183206	2.36×10^{-5}
Vincristine_1818	−0.181503	2.82×10^{-5}
MK.8776_2046	−0.178324	3.90×10^{-5}
BPD.00008900_1998	−0.172334	7.10×10^{-5}
AZ960_1250	−0.171617	7.62×10^{-5}
BDP.00009066_1866	−0.170066	8.87×10^{-5}
Pevonedistat_1529	−0.169854	9.05×10^{-5}
Gefitinib_1010	−0.165321	0.00014
Osimertinib_1919	−0.162261	0.000186
MG.132_1862	−0.162138	0.000188
Obatoclax.Mesylate_1068	−0.152063	0.000466
Acetalax_1804	−0.151833	0.000475
VSP34_8731_1734	−0.150992	0.000512
Ribociclib_1632	−0.147753	0.000676
Paclitaxel_1080	−0.146595	0.000745
PRT062607_1631	−0.146517	0.00075
PF.4708671_1129	−0.146412	0.000757
ZM447439_1050	−0.140669	0.001218
OTX015_1626	−0.139955	0.001291
Crizotinib_1083	−0.13817	0.00149
Vinblastine_1004	−0.137122	0.00162
XAV939_1268	−0.13602	0.001768
WEHI.539_1997	−0.131442	0.002523
NVP.ADW742_1932	−0.130463	0.002719
WZ4003_1614	−0.127968	0.003282
JAK_8517_1739	−0.126564	0.003643
Gemcitabine_1190	−0.126504	0.003659
VE.822_1613	−0.126482	0.003665
Savolitinib_1936	−0.120486	0.00566
Wee1.Inhibitor_1046	−0.11553	0.007996
Dabrafenib_1373	−0.115219	0.008168
Dactolisib_1057	−0.110898	0.010921
Palbociclib_1054	−0.110685	0.011076
I.BET.762_1624	−0.109801	0.011739
Fludarabine_1813	−0.104933	0.016061
Tozasertib_1096	−0.104052	0.016977

(Continued)

Table S4: *Continued*

Symbol	Correlation	*p* value
Ulixertinib_2047	−0.103856	0.017186
Podophyllotoxin.bromid-e_1825	−0.101099	0.020389
Eg5_9814_1712	−0.096087	0.027553
Camptothecin_1003	−0.094676	0.029926
Telomerase.Inhibitor.I-X_1930	−0.090647	0.037681
AZD5363_1916	−0.089711	0.039711
Dactinomycin_1811	−0.088218	0.043136
LJI308_2107	0.085905	0.048935
Nutlin.3a...._1047	0.088989	0.041338
Pyridostatin_2044	0.08908	0.041129
Nelarabine_1814	0.09079	0.03738
ML323_1629	0.091217	0.03649
Entinostat_1593	0.096294	0.027219
KRAS..G12C..Inhibito-r.12_1855	0.098238	0.024249
Zoledronate_1802	0.099541	0.02242
GSK1904529A_1093	0.100914	0.020621
AZD4547_1786	0.102272	0.018968
Carmustine_1807	0.106935	0.014139
Oxaliplatin_1806	0.107129	0.013964
UMI.77_1939	0.110944	0.010888
Daporinad_1248	0.114213	0.008747
JAK1_8709_1718	0.116518	0.007471
Wnt.C59_1622	0.119468	0.006083
Fulvestrant_1816	0.119543	0.006051
MIRA.1_1931	0.120931	0.005484
Olaparib_1017	0.122127	0.005035
LCL161_1557	0.125563	0.003922
AGI.5198_1913	0.128694	0.003108
PRIMA.1MET_1131	0.129801	0.002859

Table S4: *Continued*

Symbol	Correlation	*p* value
EPZ5676_1563	0.130038	0.002808
Nilotinib_1013	0.131514	0.00251
IRAK4_4710_1716	0.133863	0.002093
Elephantin_1835	0.137264	0.001602
Gallibiscoquinazole_1830	0.139496	0.001339
Tamoxifen_1199	0.146159	0.000773
AT13148_2170	0.150142	0.000551
Sepantronium.bromid-e_1941	0.151832	0.000475
Cyclophosphamide_1512	0.152805	0.000437
Venetoclax_1909	0.162482	0.000182
Sorafenib_1085	0.168536	0.000103
Niraparib_1177	0.170607	8.41×10^{-5}
PCI.34051_1621	0.180953	2.98×10^{-5}
AGI.6780_1634	0.185676	1.82×10^{-5}
ABT737_1910	0.18923	1.25×10^{-5}
GSK2578215A_1927	0.190699	1.06×10^{-5}
Fulvestrant_1200	0.190724	1.06×10^{-5}
Sabutoclax_1849	0.203251	2.61×10^{-6}
BI.2536_1086	0.211987	9.29×10^{-7}
P22077_1933	0.213026	8.19×10^{-7}
Vorinostat_1012	0.227995	1.25×10^{-7}
IAP_5620_1428	0.231128	8.28×10^{-8}
AZD1208_1449	0.236617	3.97×10^{-8}
Sinularin_1838	0.245307	1.20×10^{-8}
BIBR.1532_2043	0.248687	7.42×10^{-9}
Dihydrorotenone_1827	0.259866	1.45×10^{-9}
GSK591_2110	0.262839	9.26×10^{-10}
AZD5991_1720	0.331286	6.13×10^{-15}
SB505124_1194	0.358308	2.23×10^{-17}
TAF1_5496_1732	0.36205	9.79×10^{-18}
Doramapimod_1042	0.403114	5.68×10^{-22}
